# Supplementary material for: Motivational Interviewing Training: A Case-Based Curriculum for Preclinical Medical Students
Source: MedEdPORTAL. 2021 Feb 12;17:11104. doi: 10.15766/mep_2374-8265.11104 (PMC7880250; doi:10.15766/mep_2374-8265.11104)
Supplement: Supplementary file 1 — Presurvey.docxMI Presentation.pptxMI Demonstration Script.docxTransparent Outline for MI Activity.docxMICA Evaluation Tool.docPractice Cases.docxMI Summary Sheet.docxEvaluated Cases.docxOARS Tracking Sheet.docChange Talk Tracking Sheet.docMI Evaluated Session Sample Schedule.xlsxActing Patient Experience Scale.docxPostsurvey.docxFacilitator Guide.docx [file mep_2374-8265.11104-s001.zip › A. Presurvey.docx]

**MI Activity Presurvey**

Create a unique ID that you will remember next session. Please avoid any identifying information, such as your name or date of birth.  We recommend you use the name of your favorite pet and your best friend's birthday (e.g., Spot112295): ___________________

1. Which of the following is a basic principle of Motivational Interviewing?

1. Providing information about possible consequences of the patient’s actions in order to frighten them into positive behavioral change
2. Vetoing the patient’s control of behavioral change and making decisions
3. Decreasing the patient’s confidence in their ability to make behavioral changes without help
4. Pushing back against the patient’s resistance until they are ready to change
5. **Developing discrepancy between the patient’s goals and their current behavior in order to stimulate motivation to change**

2. The health care provider’s role in Motivational Interviewing is best described as which of the following?

- 1. **Ask the patient questions to elicit their reasons to change and ideas about how to change**
  2. Inform the patient of the best course of action
  3. Refrain from discussing the health behavior change with the patient
  4. Make sure the patient knows how dangerous their current behavior is
  5. Bring in others to confront the patient with the need to change

3. A patient brings in his son, a 4-year-old male, for follow-up after being hospitalized for asthma. This is his third asthma exacerbation in three months. He has completed a course of oral steroids and is current taking medications as prescribed for his asthma. Your last visit note indicates that his father is a smoker, and you can smell cigarette smoke on him as he enters the room. Which of the following responses by you, the physician, would be most appropriate?

1. “Don’t you care about your son?”
2. “Your smoking is making your son’s asthma worse”
3. **“I’m concerned about your son’s asthma control. Would it be OK if we discussed your smoking?”**
4. “I suggest you quit smoking”
5. “Why can’t you quit smoking?”

4. Advice can be offered to a patient with in a Motivational Interviewing framework under which of the following conditions?

1. When the physician knows what the patient needs to do to fix improve their health
2. When the patient could die if the physician does not tell the patient what to do
3. **When the patient asks for advice and the physician follows up by assessing motivation after the advice is given**
4. When the patient is resisting all the physician’s efforts to elicit change talk
5. When the patient is in the Precontemplation stage of change

5. List 3 core skills used in MI:

a.

b.

c.

6. OARS stands for:

O:

A:

R:

S:

**For the questions below, please use the following rating scale:**

1= Strongly Disagree 2= Disagree 3= Neutral 4=Agree 5=Strongly Agree

| **Question** | **Rating (1-5)** |
| --- | --- |
| 7. I feel confident in my ability to talk with patients about behavior change, such as losing weight |  |
| 8. I feel confident in my ability to use OARS (open-ended questions, affirmations, reflections, summaries) with a patient |  |
| 9. I feel confident in my ability to resist the righting reflex with patients |  |
| 10. I feel confident in my ability to roll with patient resistance when discussing behavior change |  |
| 11. I feel confident in my ability to develop discrepancy between a patient’s health goals and their behavior |  |
| 12. I feel confident in my ability to elicit change talk from a patient |  |
| 13. As a health professional, I feel it is my responsibility to address my patients’ lifestyle behaviors, such as diet, exercise, and adherence |  |
| 14. Motivational Interviewing is an effective way to help patients engage in healthier lifestyle behaviors |  |
| 15. Health professionals can counsel patients on lifestyle changes during a 15-20 minute medical visit |  |
