# Supplementary material for: Motivational Interviewing Training: A Case-Based Curriculum for Preclinical Medical Students
Source: MedEdPORTAL. 2021 Feb 12;17:11104. doi: 10.15766/mep_2374-8265.11104 (PMC7880250; doi:10.15766/mep_2374-8265.11104)
Supplement: Supplementary file 1 — Presurvey.docxMI Presentation.pptxMI Demonstration Script.docxTransparent Outline for MI Activity.docxMICA Evaluation Tool.docPractice Cases.docxMI Summary Sheet.docxEvaluated Cases.docxOARS Tracking Sheet.docChange Talk Tracking Sheet.docMI Evaluated Session Sample Schedule.xlsxActing Patient Experience Scale.docxPostsurvey.docxFacilitator Guide.docx [file mep_2374-8265.11104-s001.zip › B. MI Presentation.pptx]

## Slide 1
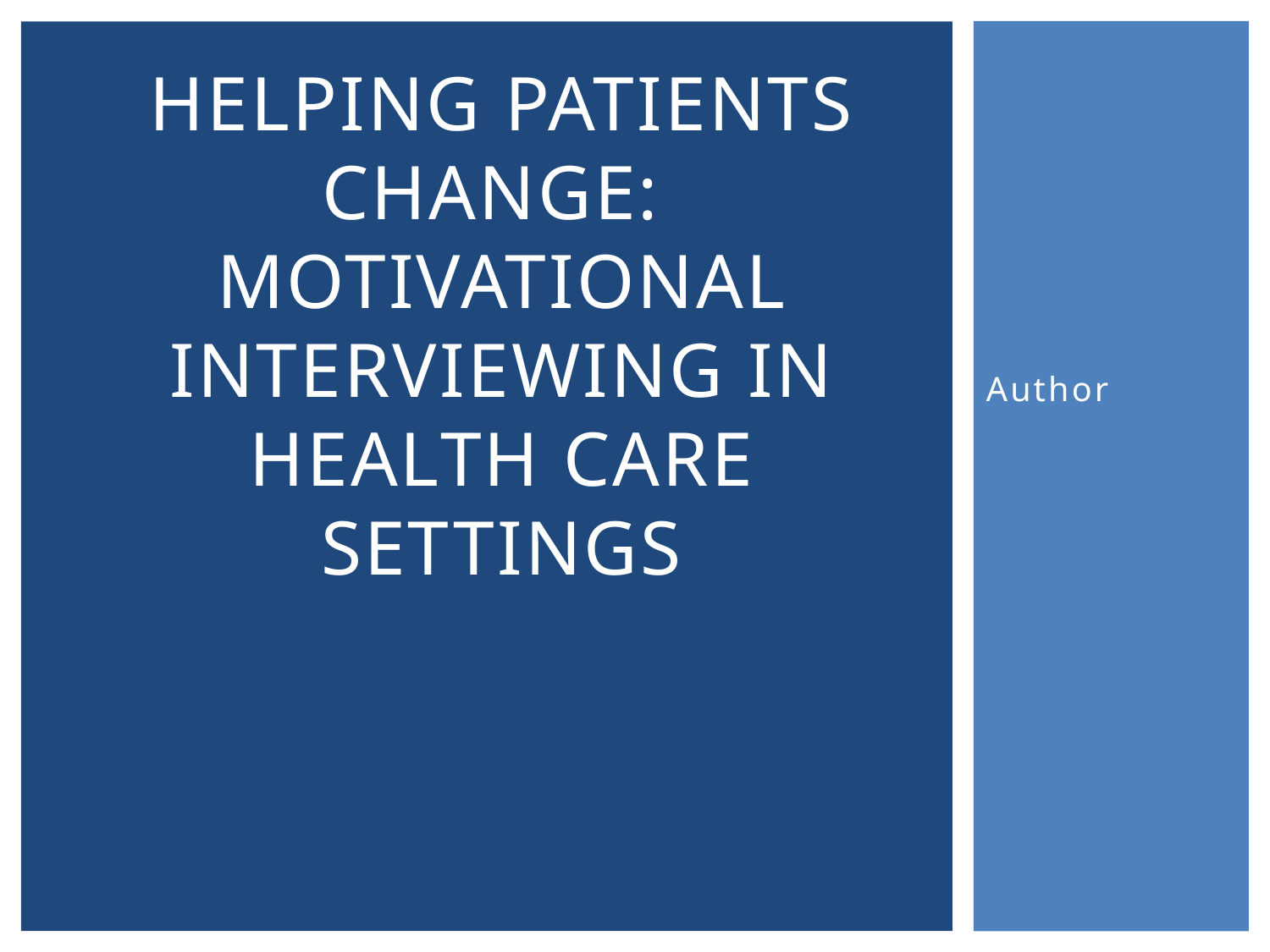

# Helping Patients Change: Motivational Interviewing in Health Care Settings
Author

## Slide 2
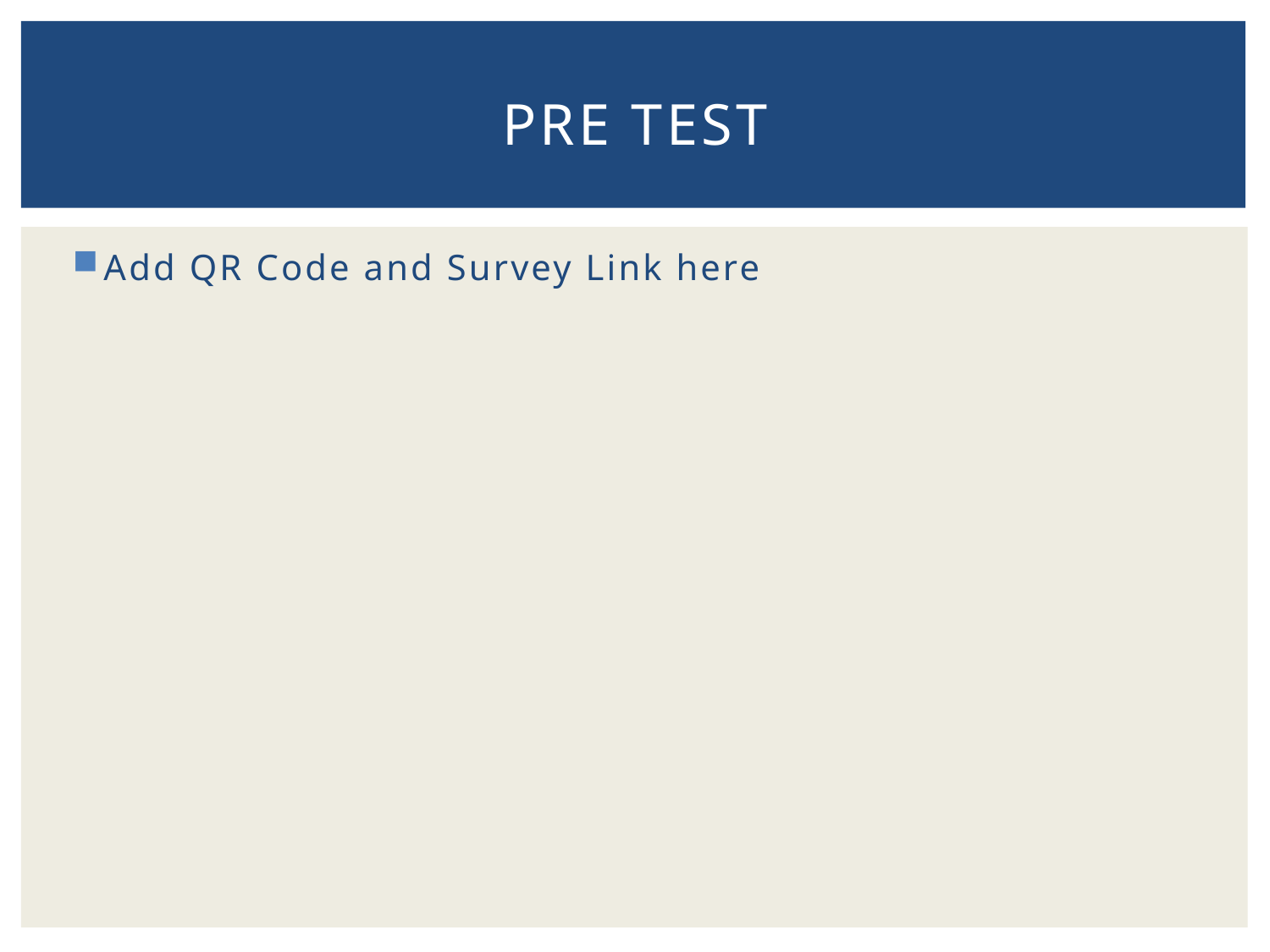

# Pre Test
Add QR Code and Survey Link here

## Slide 3
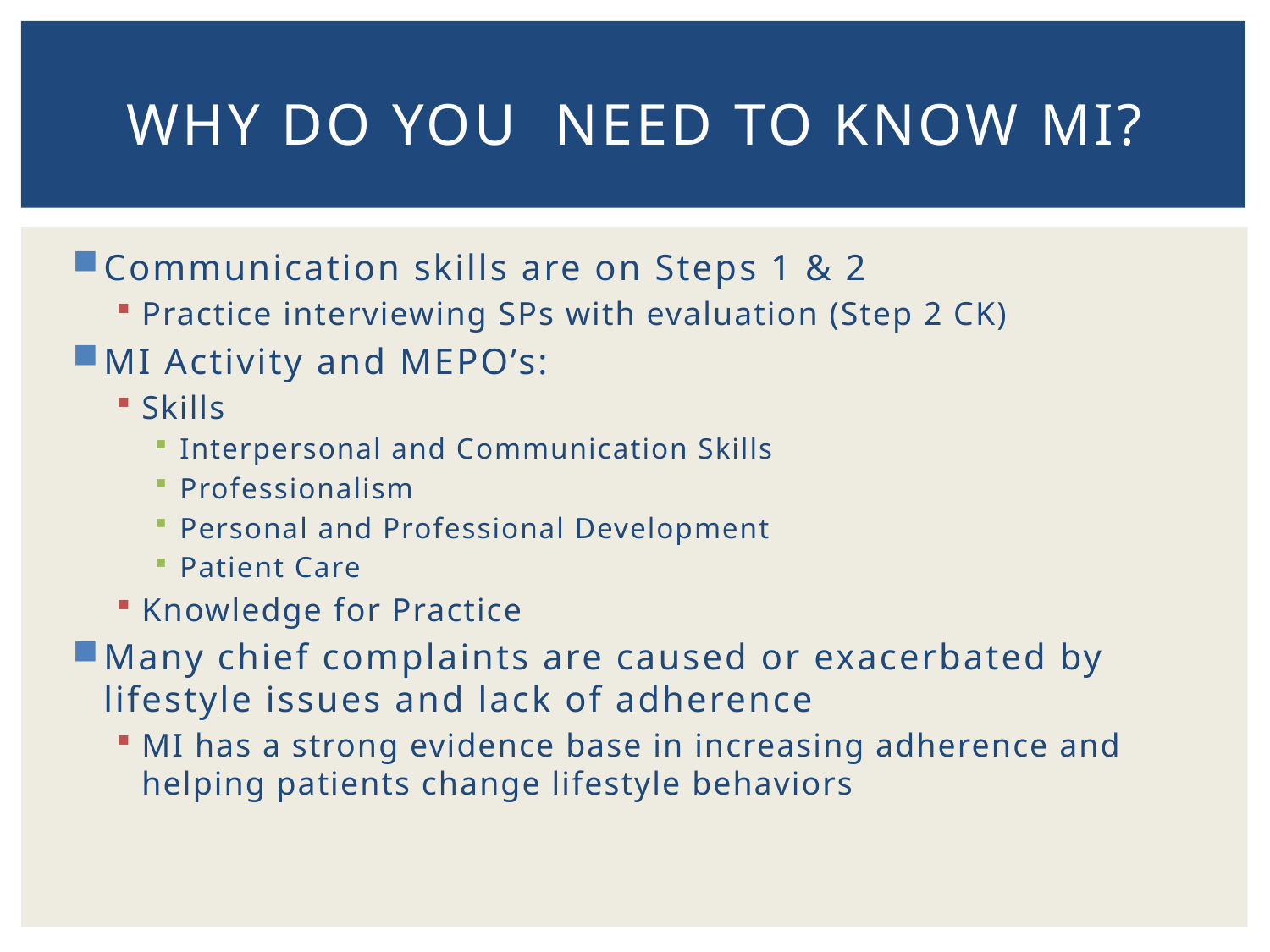

# Why do you need to know mi?
Communication skills are on Steps 1 & 2
Practice interviewing SPs with evaluation (Step 2 CK)
MI Activity and MEPO’s:
Skills
Interpersonal and Communication Skills
Professionalism
Personal and Professional Development
Patient Care
Knowledge for Practice
Many chief complaints are caused or exacerbated by lifestyle issues and lack of adherence
MI has a strong evidence base in increasing adherence and helping patients change lifestyle behaviors

## Slide 4
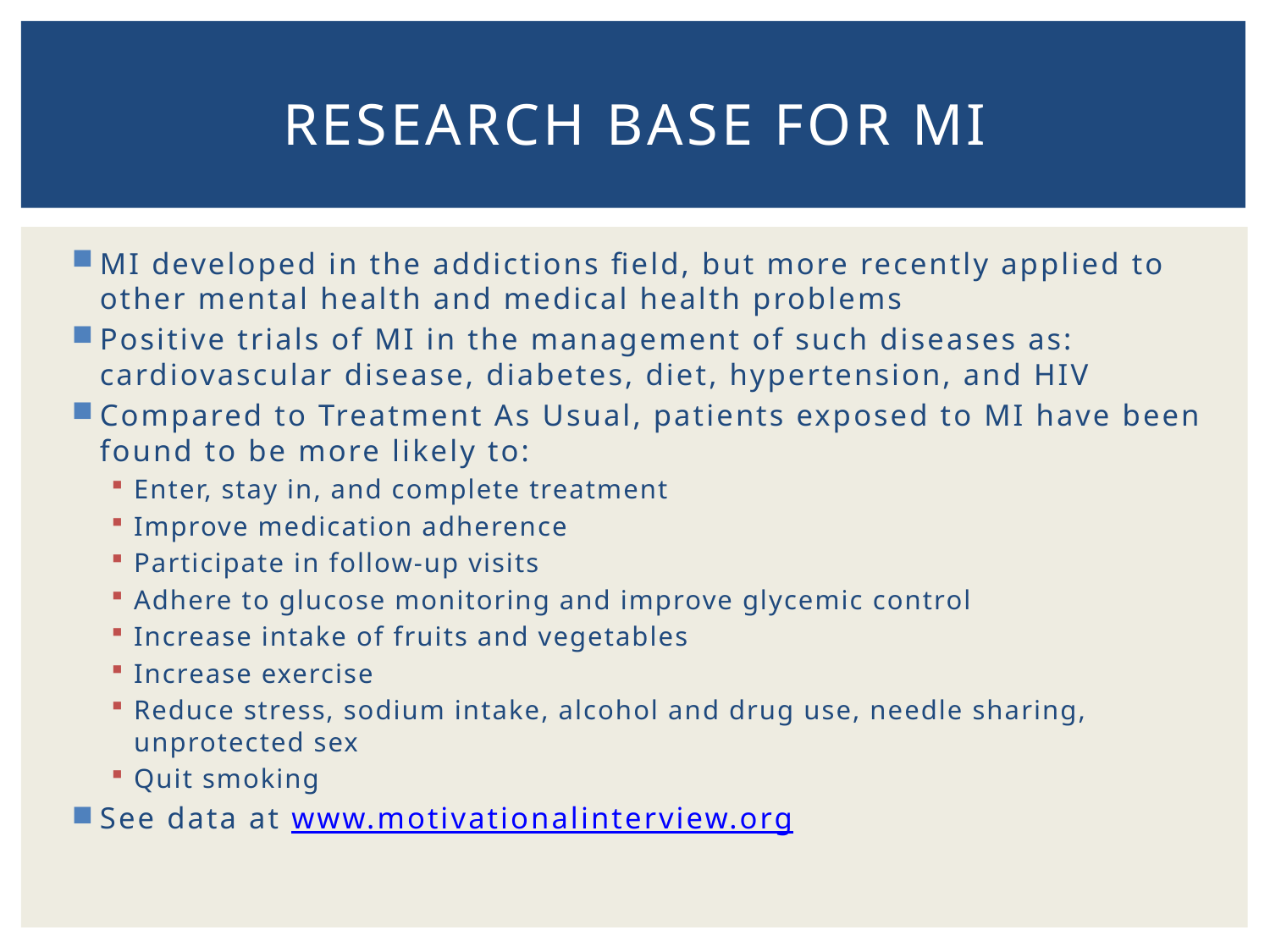

# Research base for MI
MI developed in the addictions field, but more recently applied to other mental health and medical health problems
Positive trials of MI in the management of such diseases as: cardiovascular disease, diabetes, diet, hypertension, and HIV
Compared to Treatment As Usual, patients exposed to MI have been found to be more likely to:
Enter, stay in, and complete treatment
Improve medication adherence
Participate in follow-up visits
Adhere to glucose monitoring and improve glycemic control
Increase intake of fruits and vegetables
Increase exercise
Reduce stress, sodium intake, alcohol and drug use, needle sharing, unprotected sex
Quit smoking
See data at www.motivationalinterview.org

## Slide 5
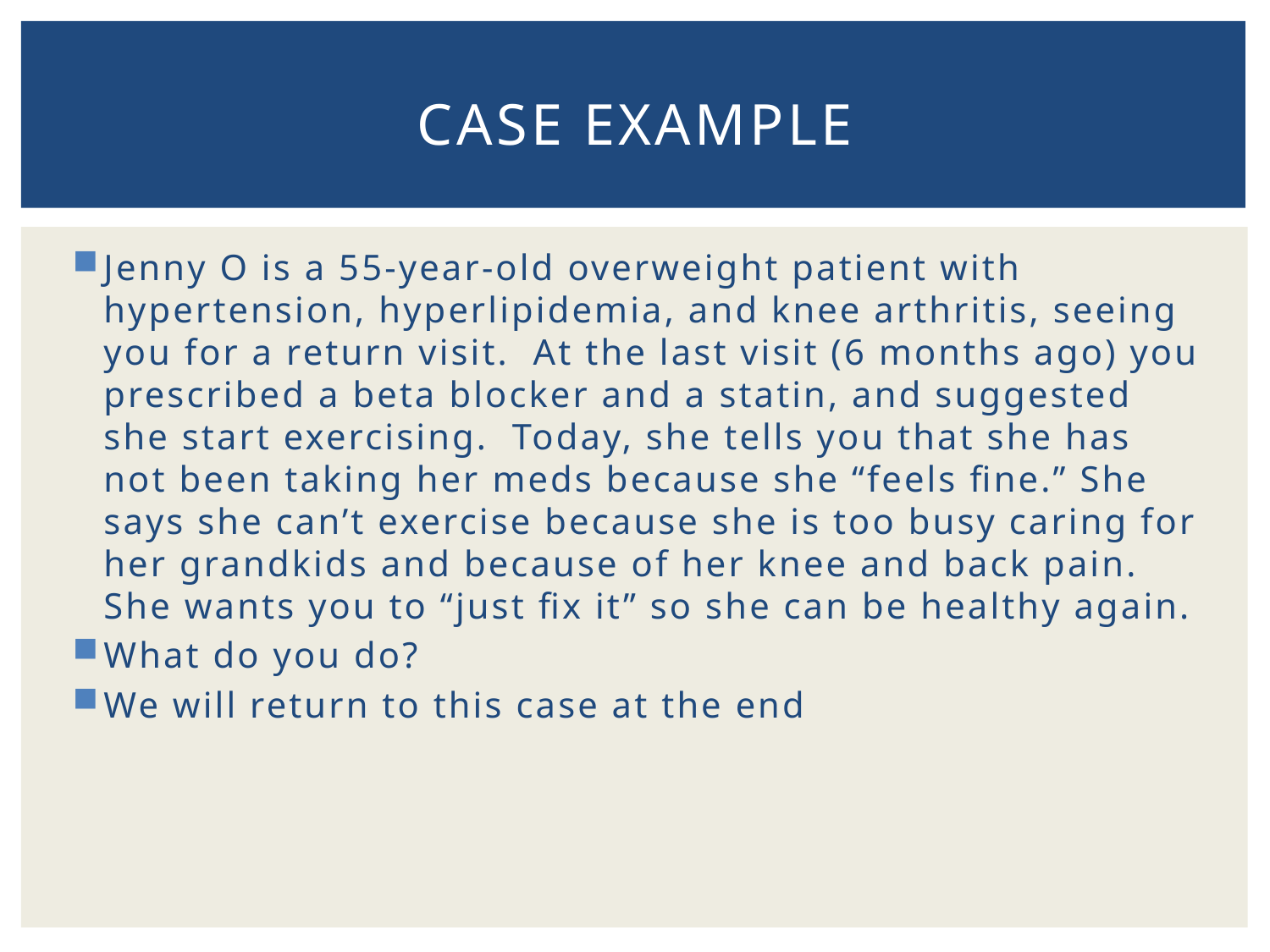

# Case example
Jenny O is a 55-year-old overweight patient with hypertension, hyperlipidemia, and knee arthritis, seeing you for a return visit. At the last visit (6 months ago) you prescribed a beta blocker and a statin, and suggested she start exercising. Today, she tells you that she has not been taking her meds because she “feels fine.” She says she can’t exercise because she is too busy caring for her grandkids and because of her knee and back pain. She wants you to “just fix it” so she can be healthy again.
What do you do?
We will return to this case at the end

## Slide 6
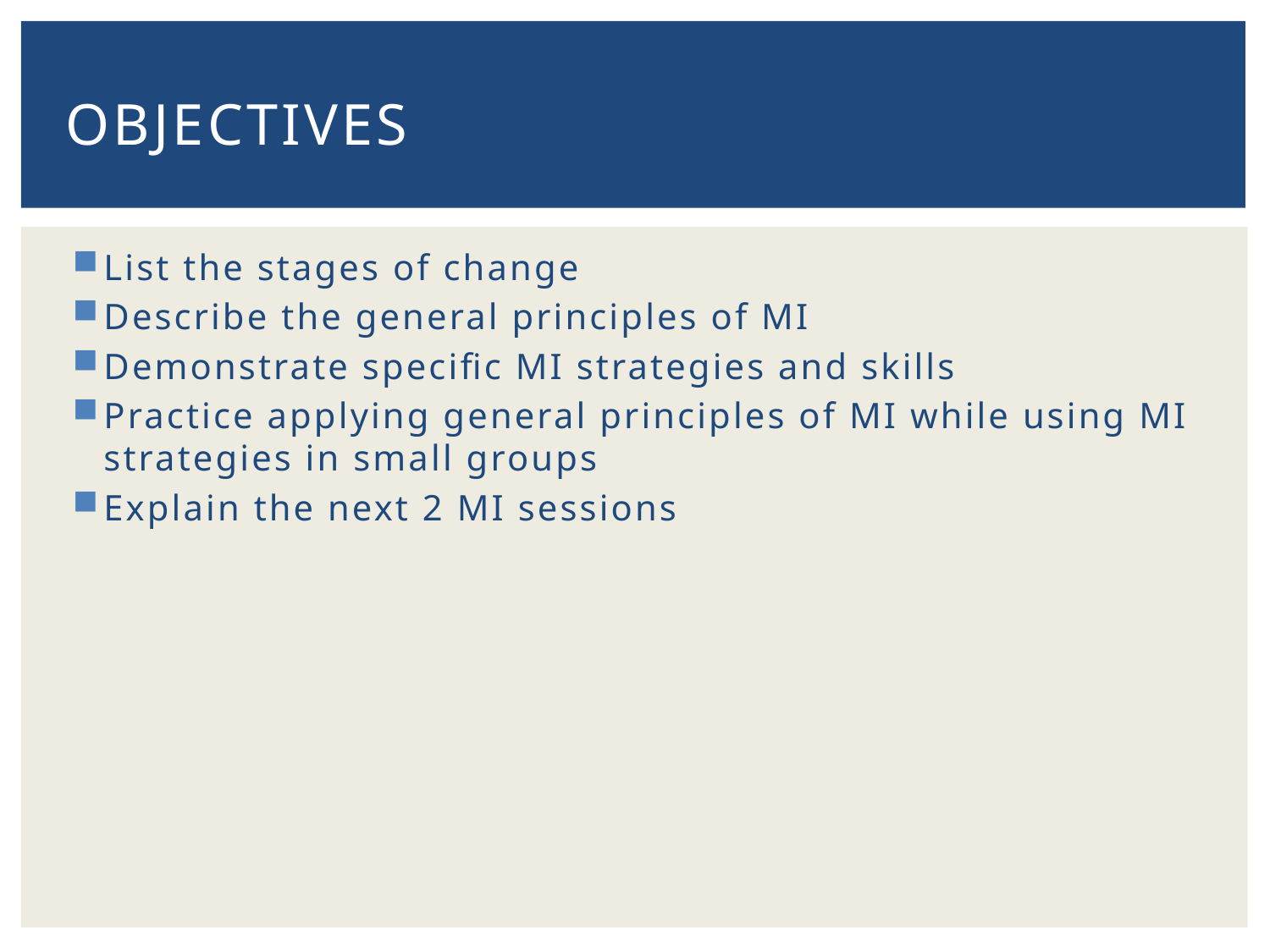

# Objectives
List the stages of change
Describe the general principles of MI
Demonstrate specific MI strategies and skills
Practice applying general principles of MI while using MI strategies in small groups
Explain the next 2 MI sessions

## Slide 7
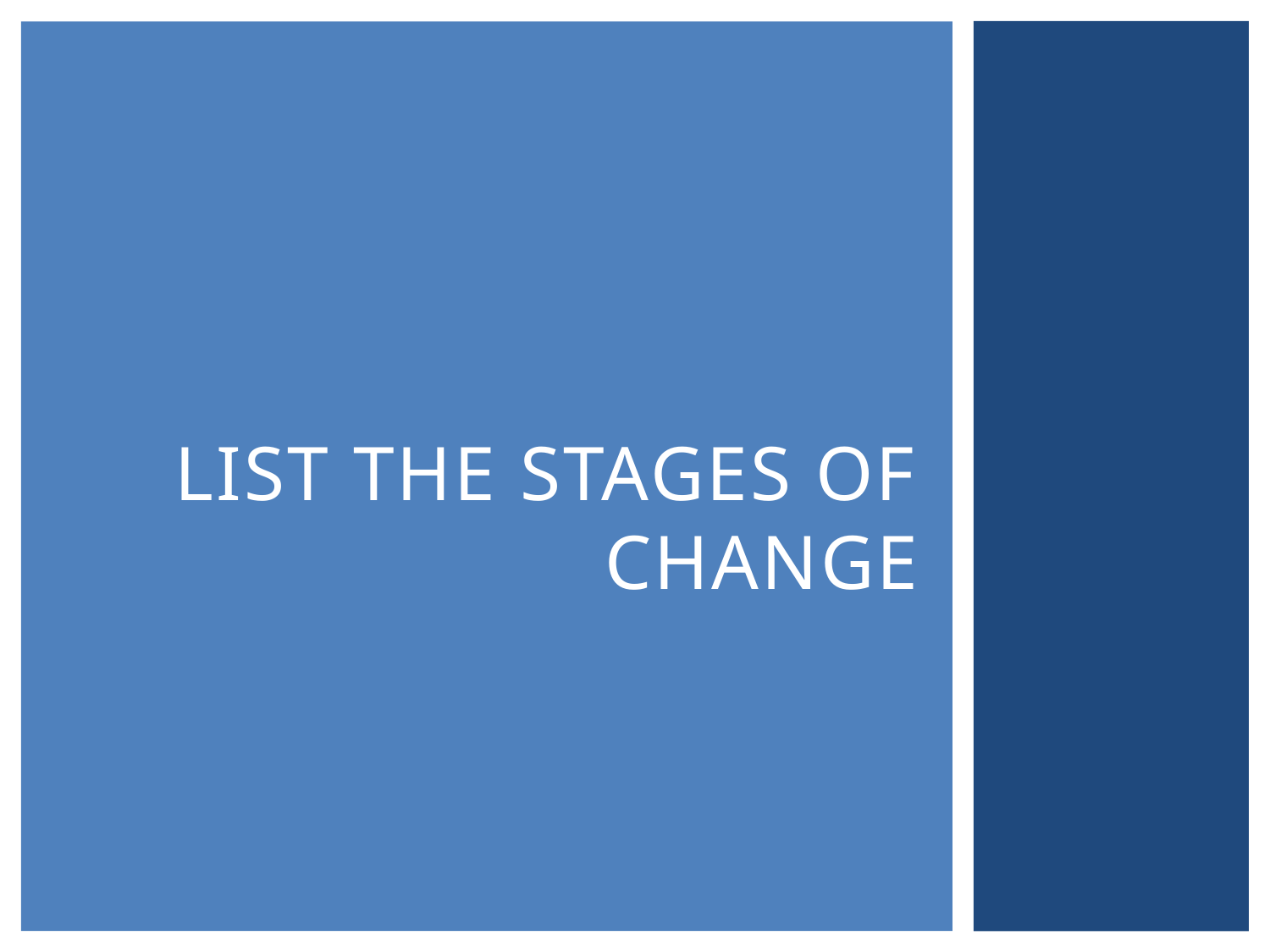

# List the Stages of Change

## Slide 8
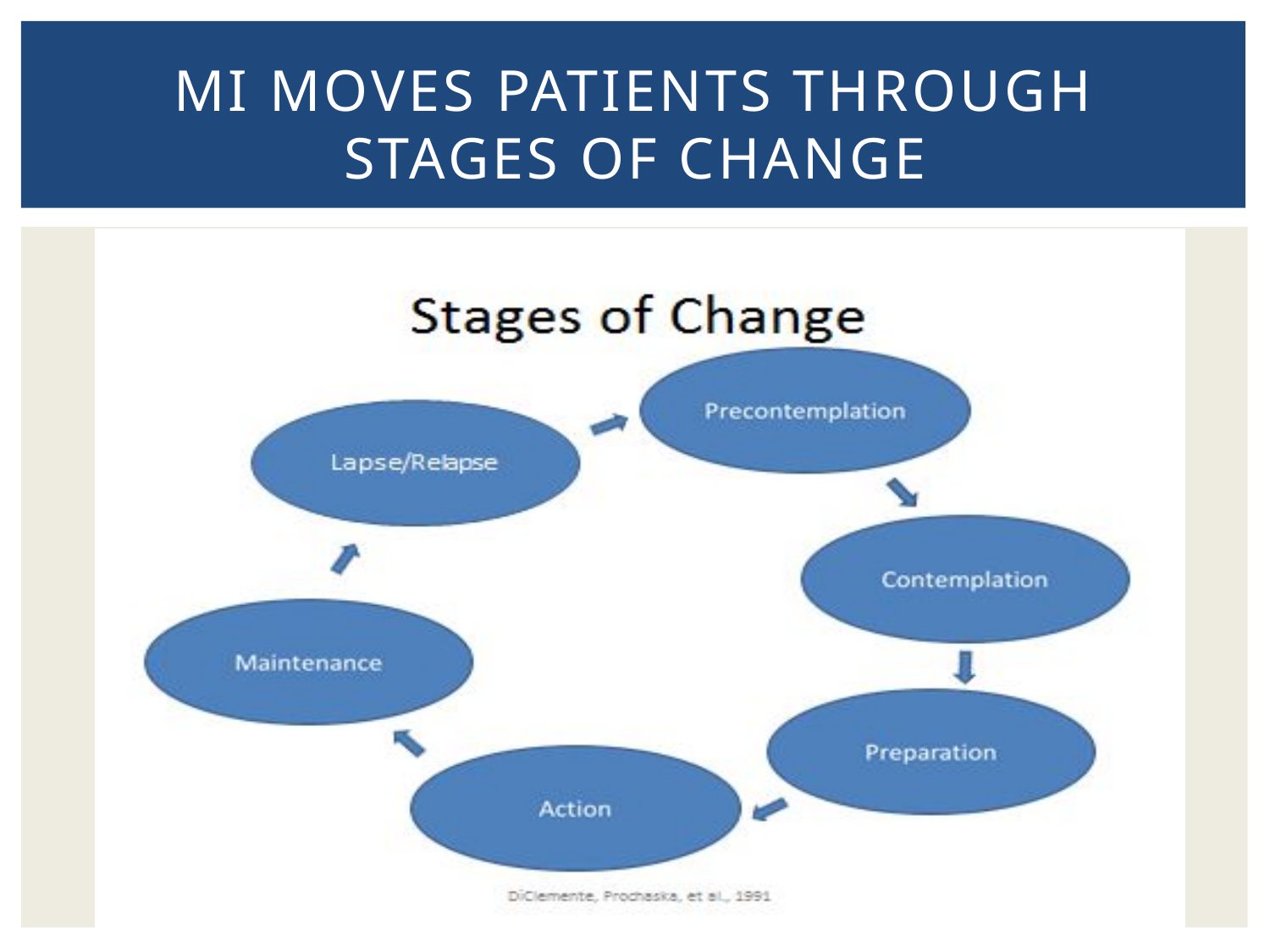

# MI moves patients through stages of change

## Slide 9
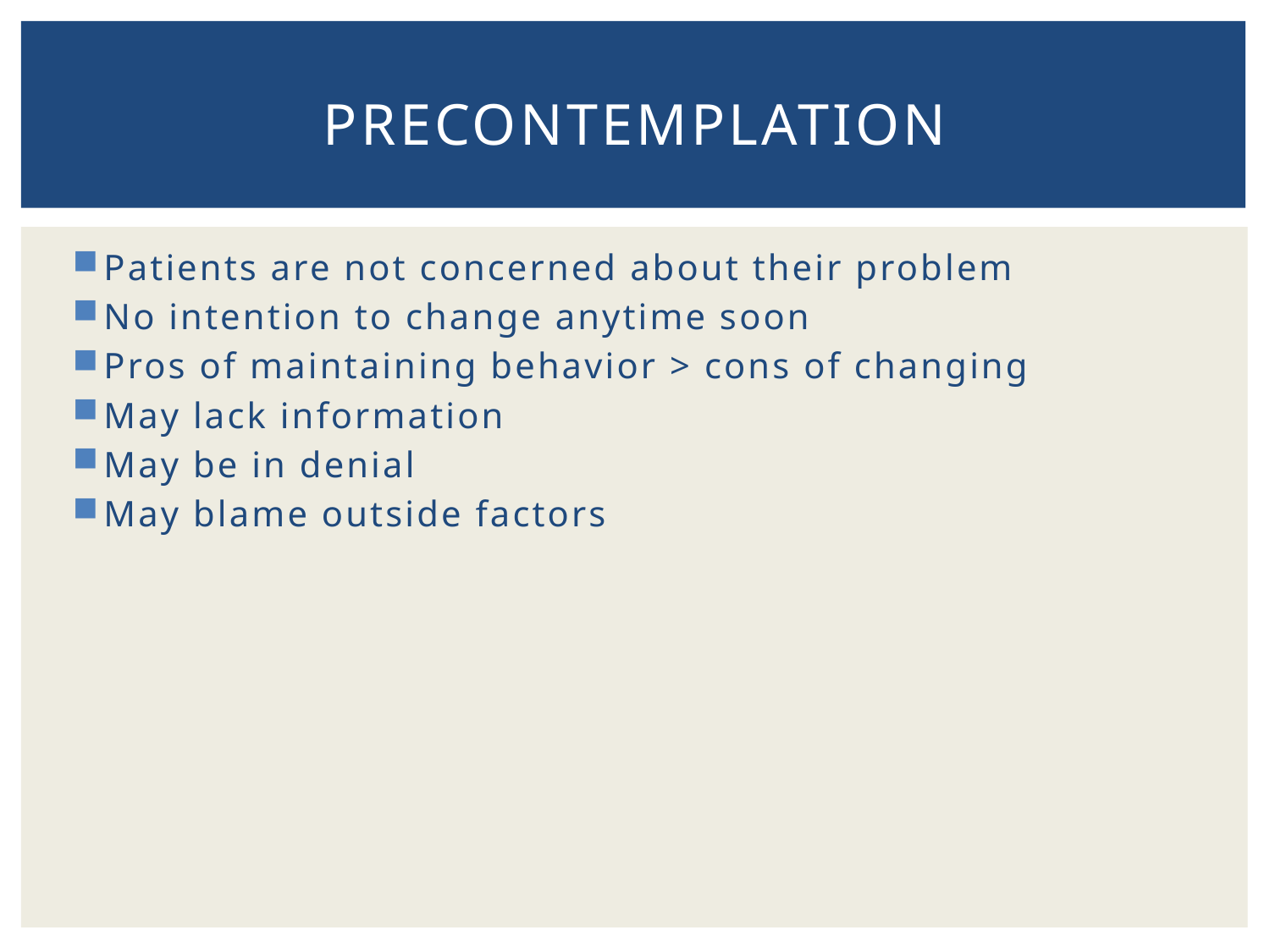

# precontemplation
Patients are not concerned about their problem
No intention to change anytime soon
Pros of maintaining behavior > cons of changing
May lack information
May be in denial
May blame outside factors

## Slide 10
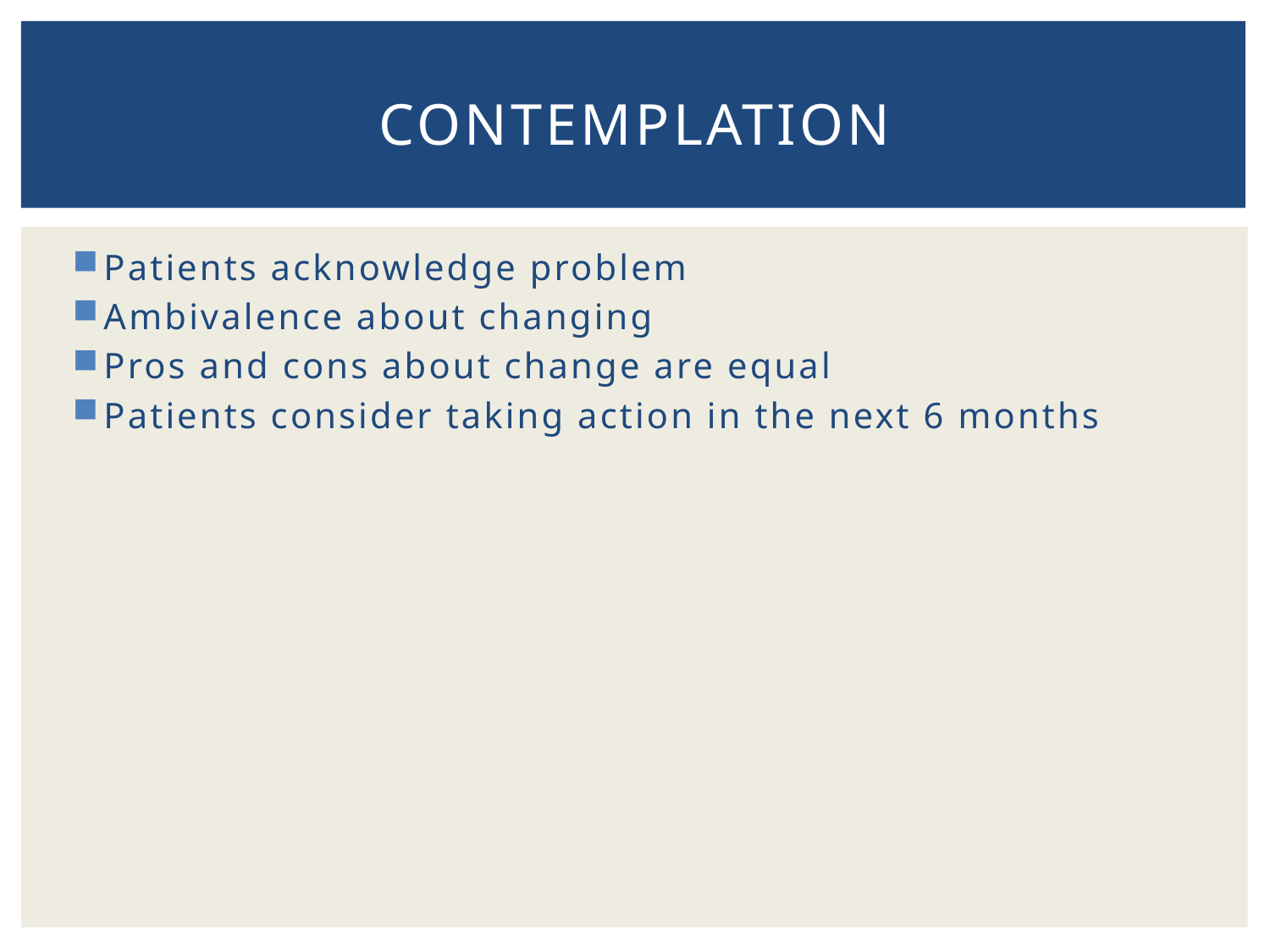

# Contemplation
Patients acknowledge problem
Ambivalence about changing
Pros and cons about change are equal
Patients consider taking action in the next 6 months

## Slide 11
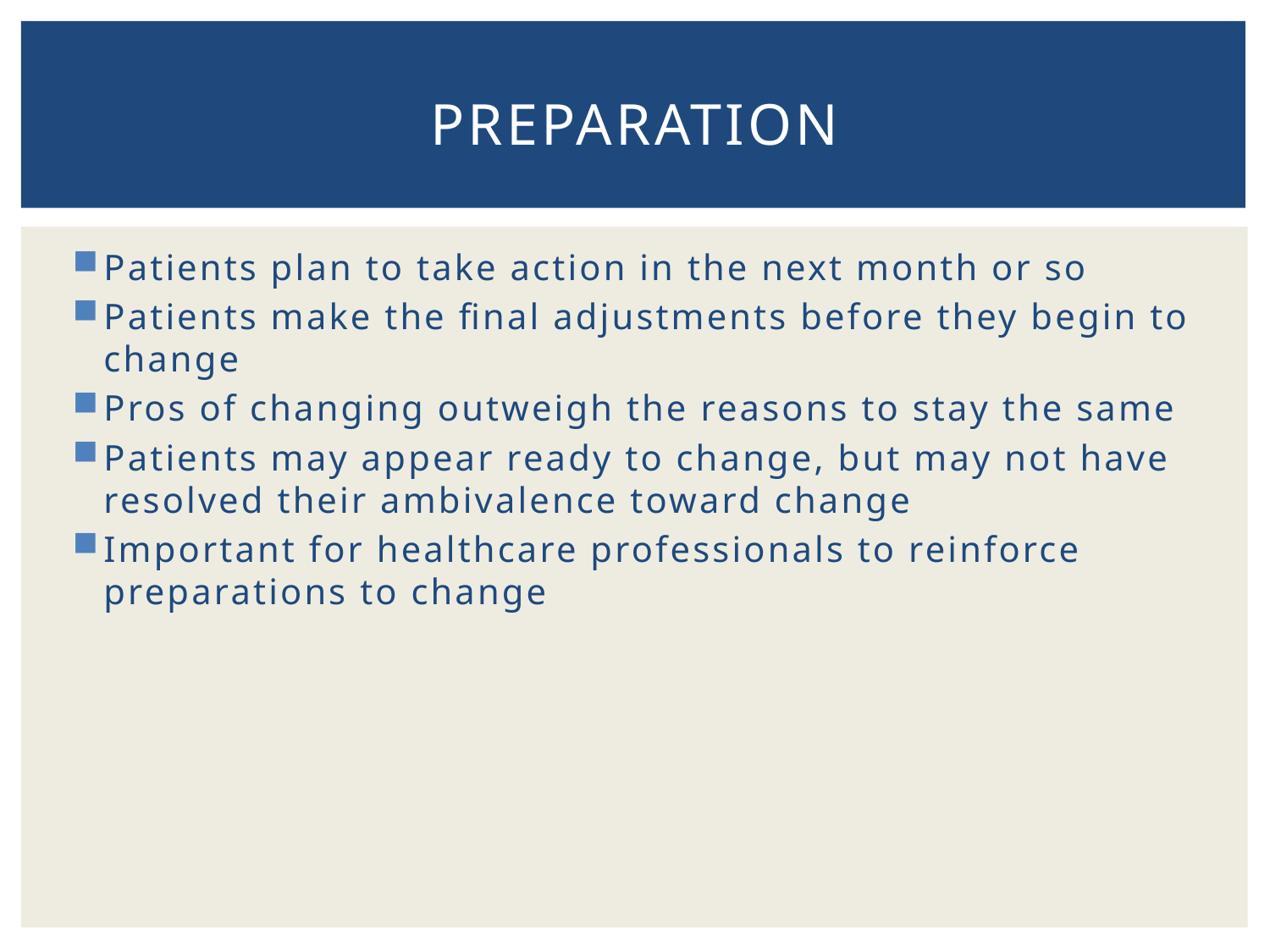

# preparation
Patients plan to take action in the next month or so
Patients make the final adjustments before they begin to change
Pros of changing outweigh the reasons to stay the same
Patients may appear ready to change, but may not have resolved their ambivalence toward change
Important for healthcare professionals to reinforce preparations to change

## Slide 12
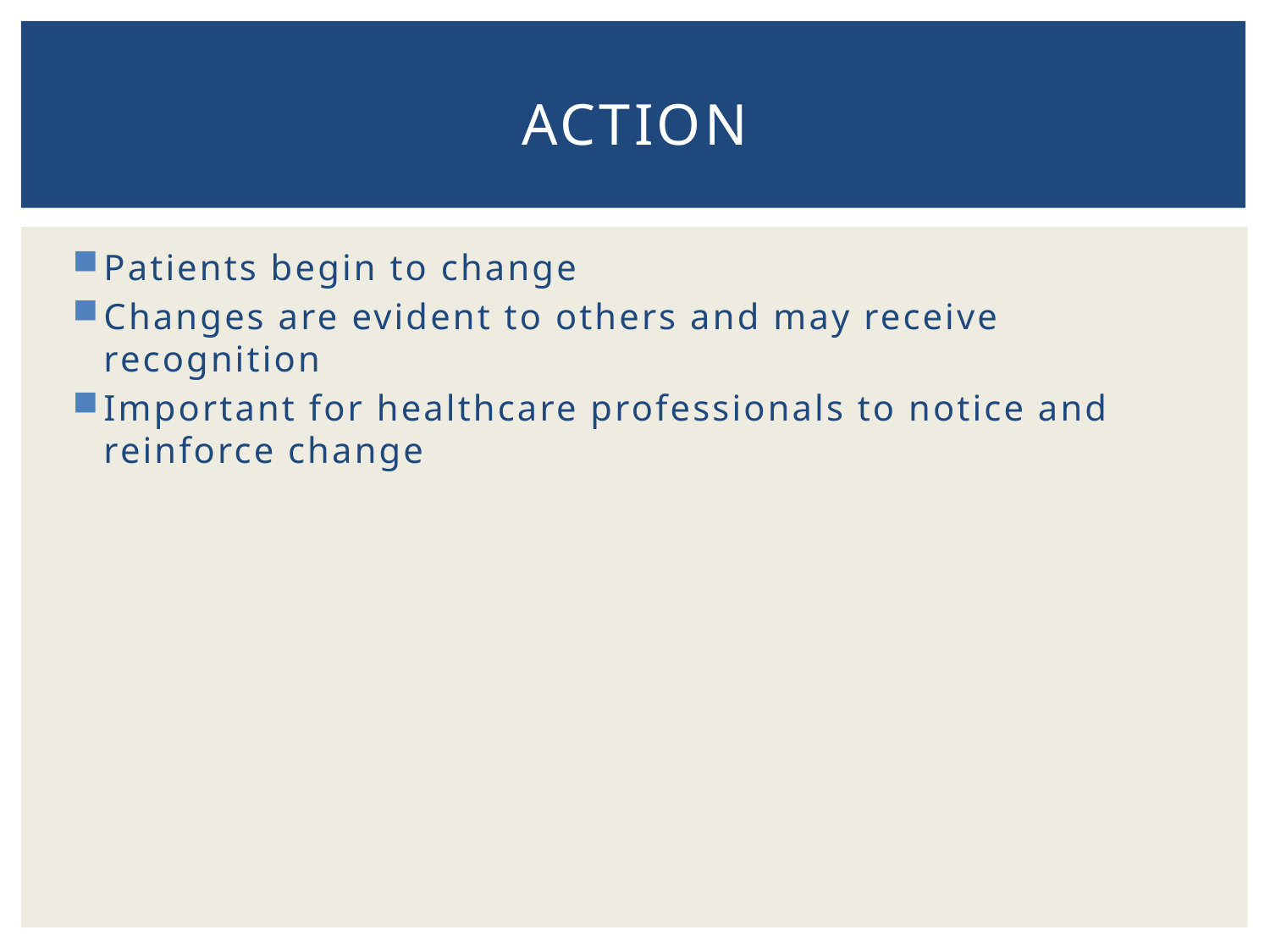

# action
Patients begin to change
Changes are evident to others and may receive recognition
Important for healthcare professionals to notice and reinforce change

## Slide 13
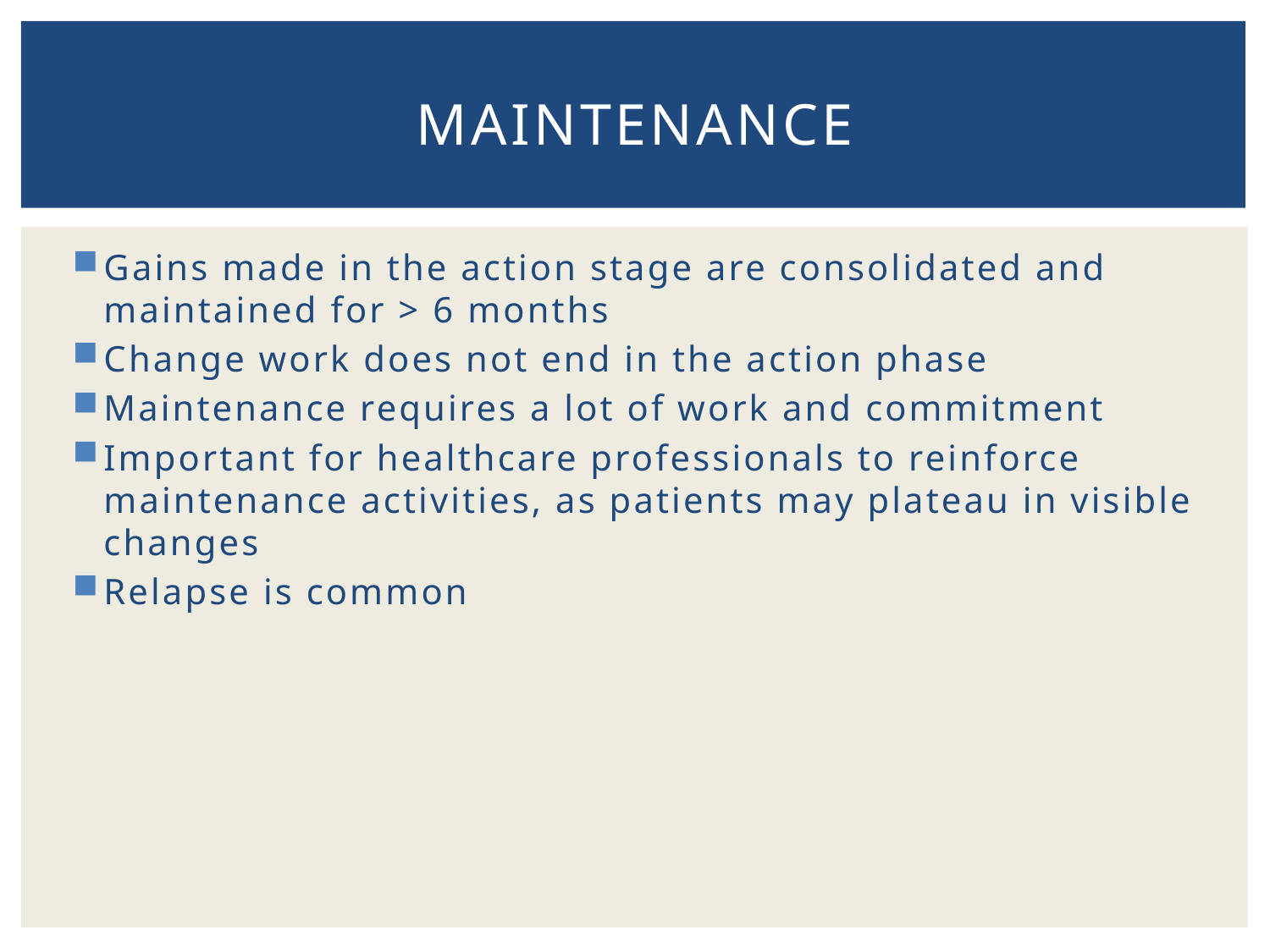

# maintenance
Gains made in the action stage are consolidated and maintained for > 6 months
Change work does not end in the action phase
Maintenance requires a lot of work and commitment
Important for healthcare professionals to reinforce maintenance activities, as patients may plateau in visible changes
Relapse is common

## Slide 14
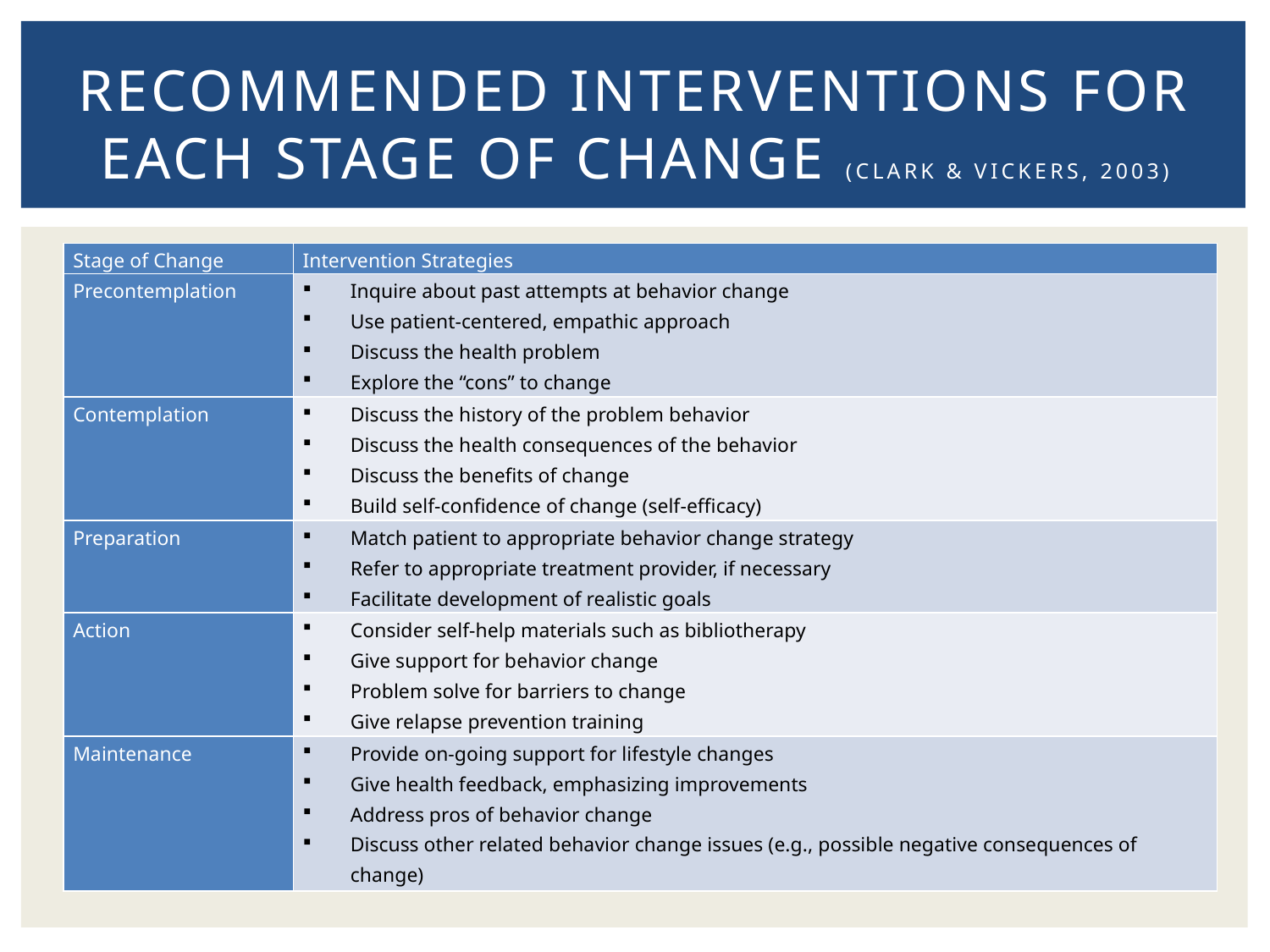

# Recommended interventions for each stage of change (Clark & Vickers, 2003)
| Stage of Change | Intervention Strategies |
| --- | --- |
| Precontemplation | Inquire about past attempts at behavior change Use patient-centered, empathic approach Discuss the health problem Explore the “cons” to change |
| Contemplation | Discuss the history of the problem behavior Discuss the health consequences of the behavior Discuss the benefits of change Build self-confidence of change (self-efficacy) |
| Preparation | Match patient to appropriate behavior change strategy Refer to appropriate treatment provider, if necessary Facilitate development of realistic goals |
| Action | Consider self-help materials such as bibliotherapy Give support for behavior change Problem solve for barriers to change Give relapse prevention training |
| Maintenance | Provide on-going support for lifestyle changes Give health feedback, emphasizing improvements Address pros of behavior change Discuss other related behavior change issues (e.g., possible negative consequences of change) |

## Slide 15
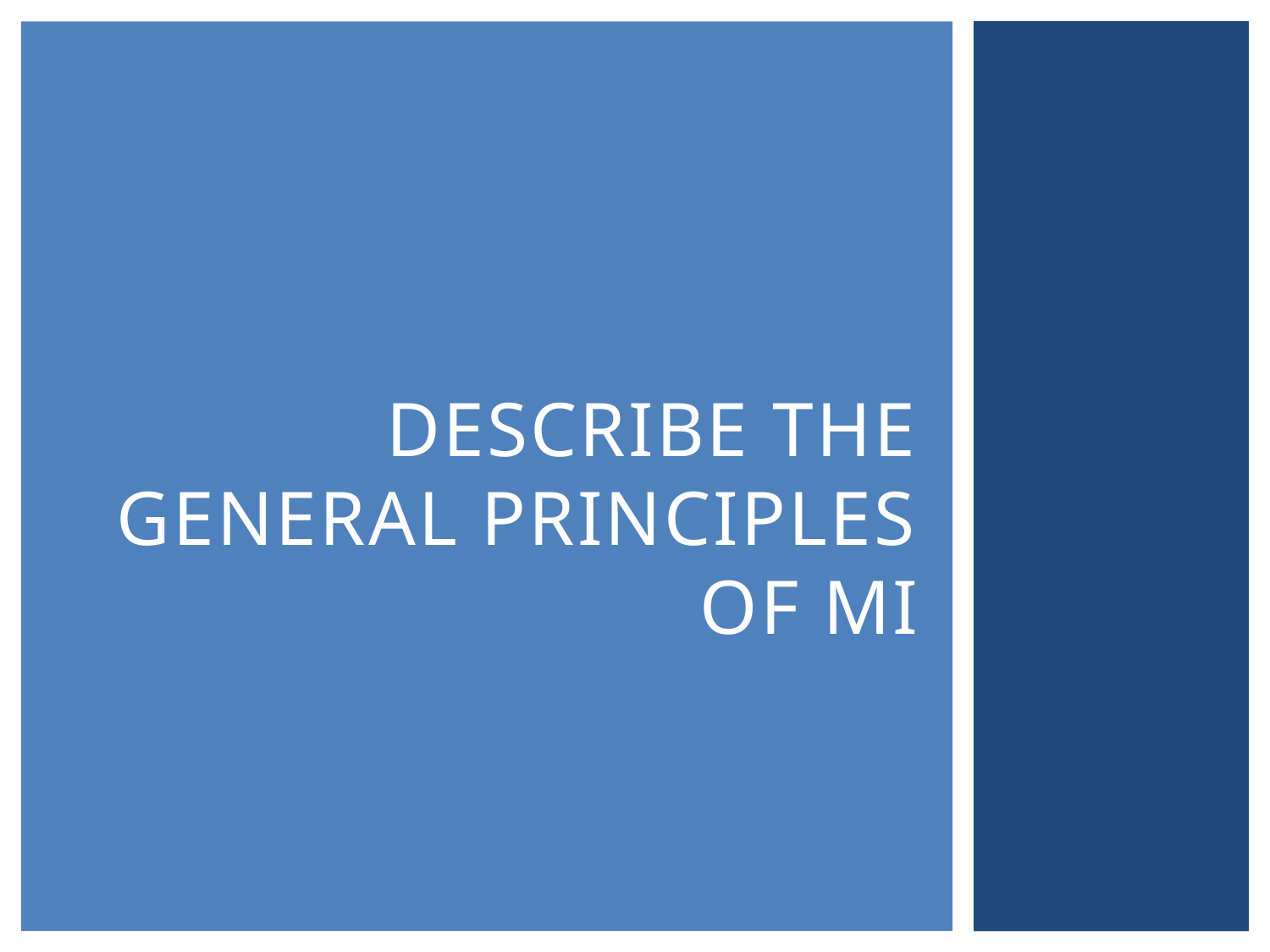

# Describe the General Principles of MI

## Slide 16
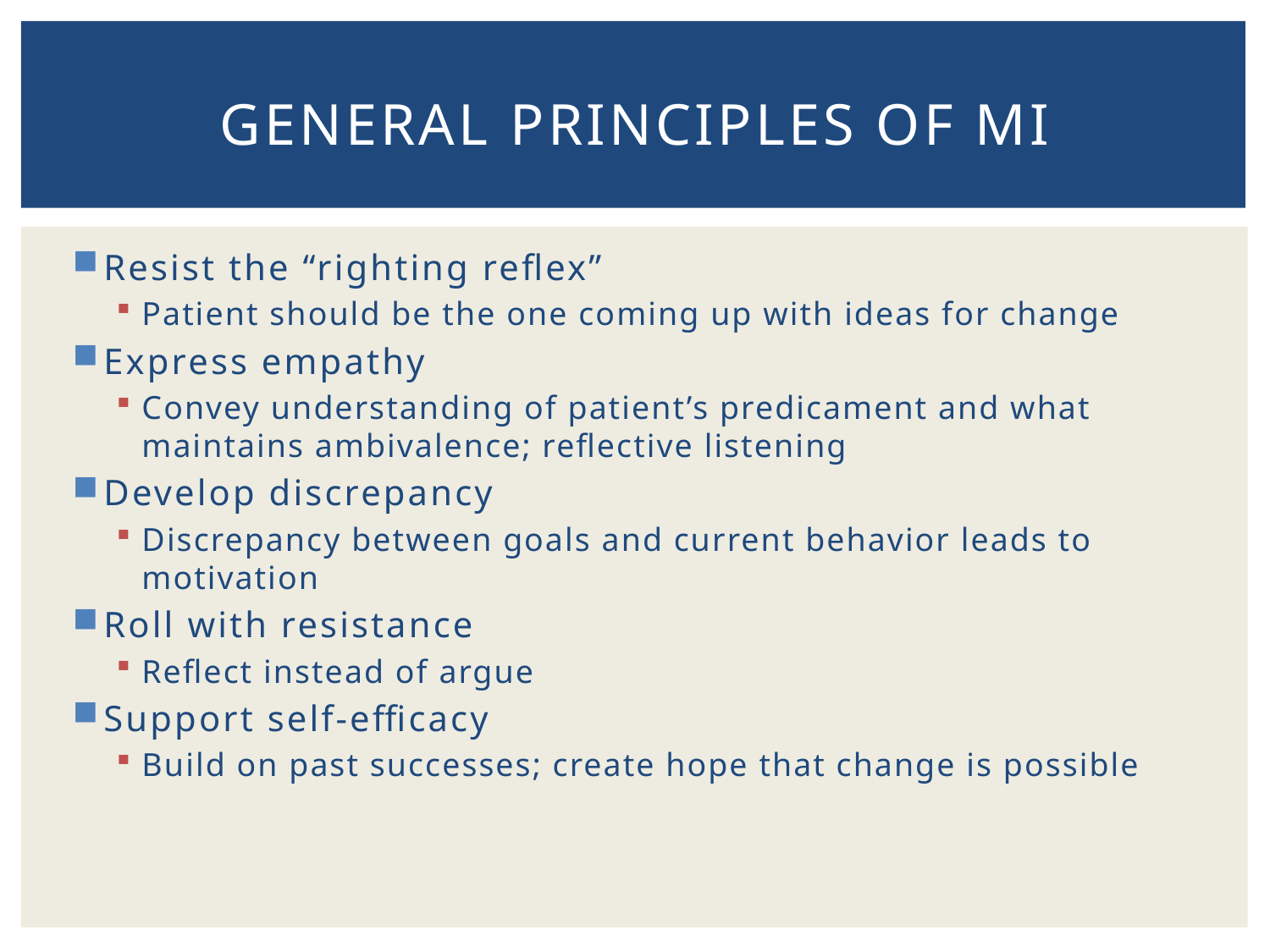

# General principles of MI
Resist the “righting reflex”
Patient should be the one coming up with ideas for change
Express empathy
Convey understanding of patient’s predicament and what maintains ambivalence; reflective listening
Develop discrepancy
Discrepancy between goals and current behavior leads to motivation
Roll with resistance
Reflect instead of argue
Support self-efficacy
Build on past successes; create hope that change is possible

## Slide 17
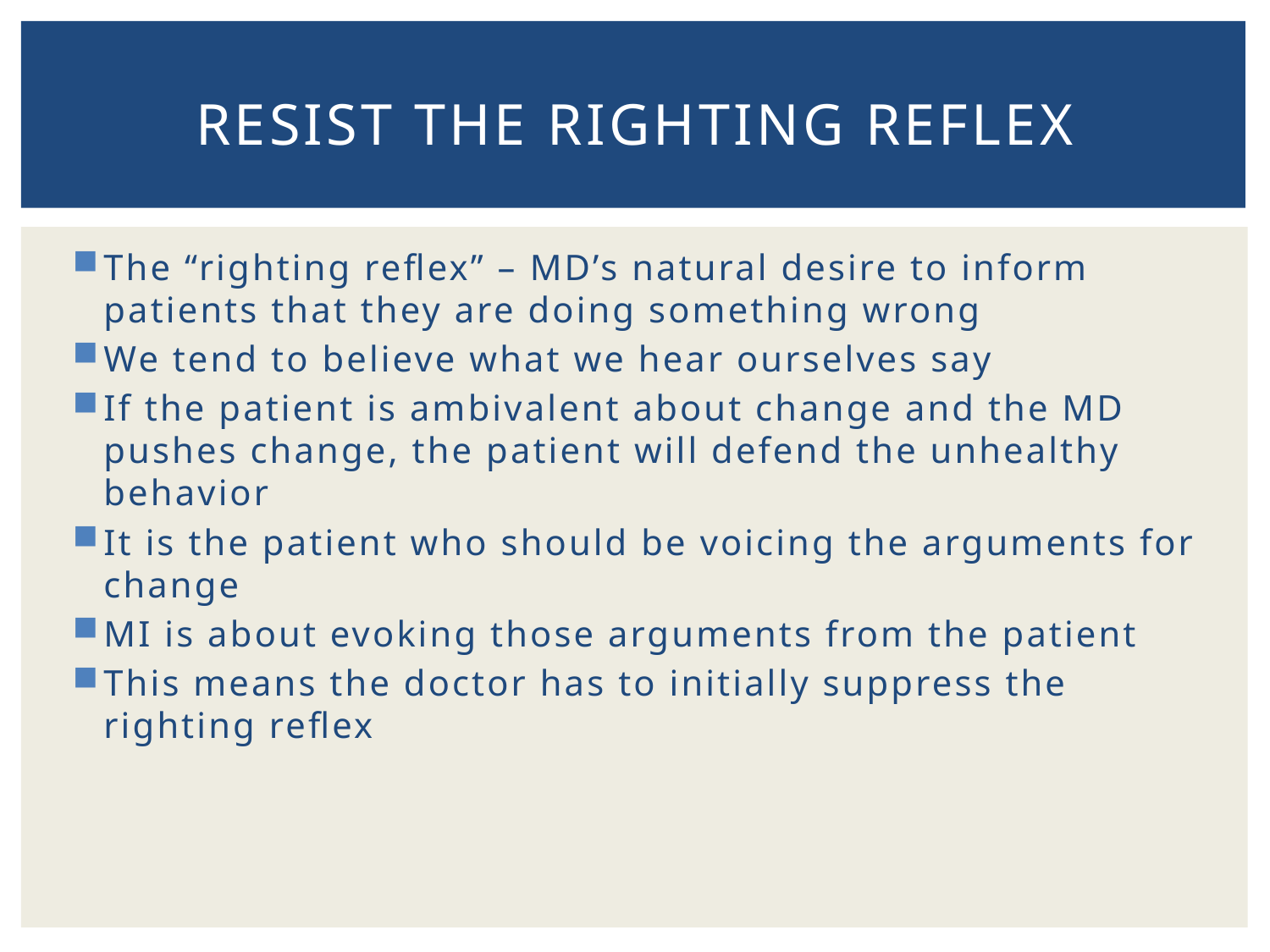

# Resist the Righting Reflex
The “righting reflex” – MD’s natural desire to inform patients that they are doing something wrong
We tend to believe what we hear ourselves say
If the patient is ambivalent about change and the MD pushes change, the patient will defend the unhealthy behavior
It is the patient who should be voicing the arguments for change
MI is about evoking those arguments from the patient
This means the doctor has to initially suppress the righting reflex

## Slide 18
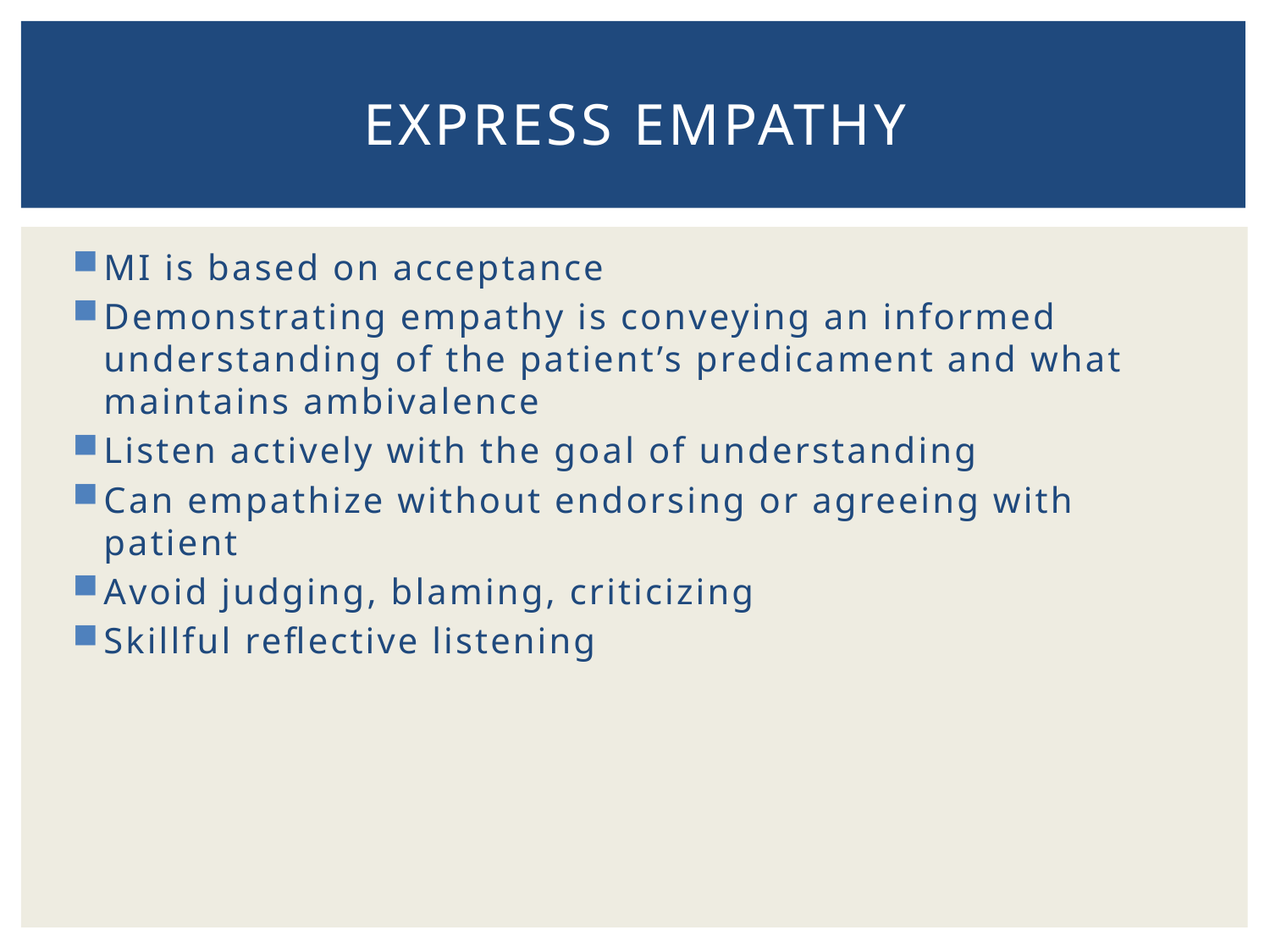

# Express empathy
MI is based on acceptance
Demonstrating empathy is conveying an informed understanding of the patient’s predicament and what maintains ambivalence
Listen actively with the goal of understanding
Can empathize without endorsing or agreeing with patient
Avoid judging, blaming, criticizing
Skillful reflective listening

## Slide 19
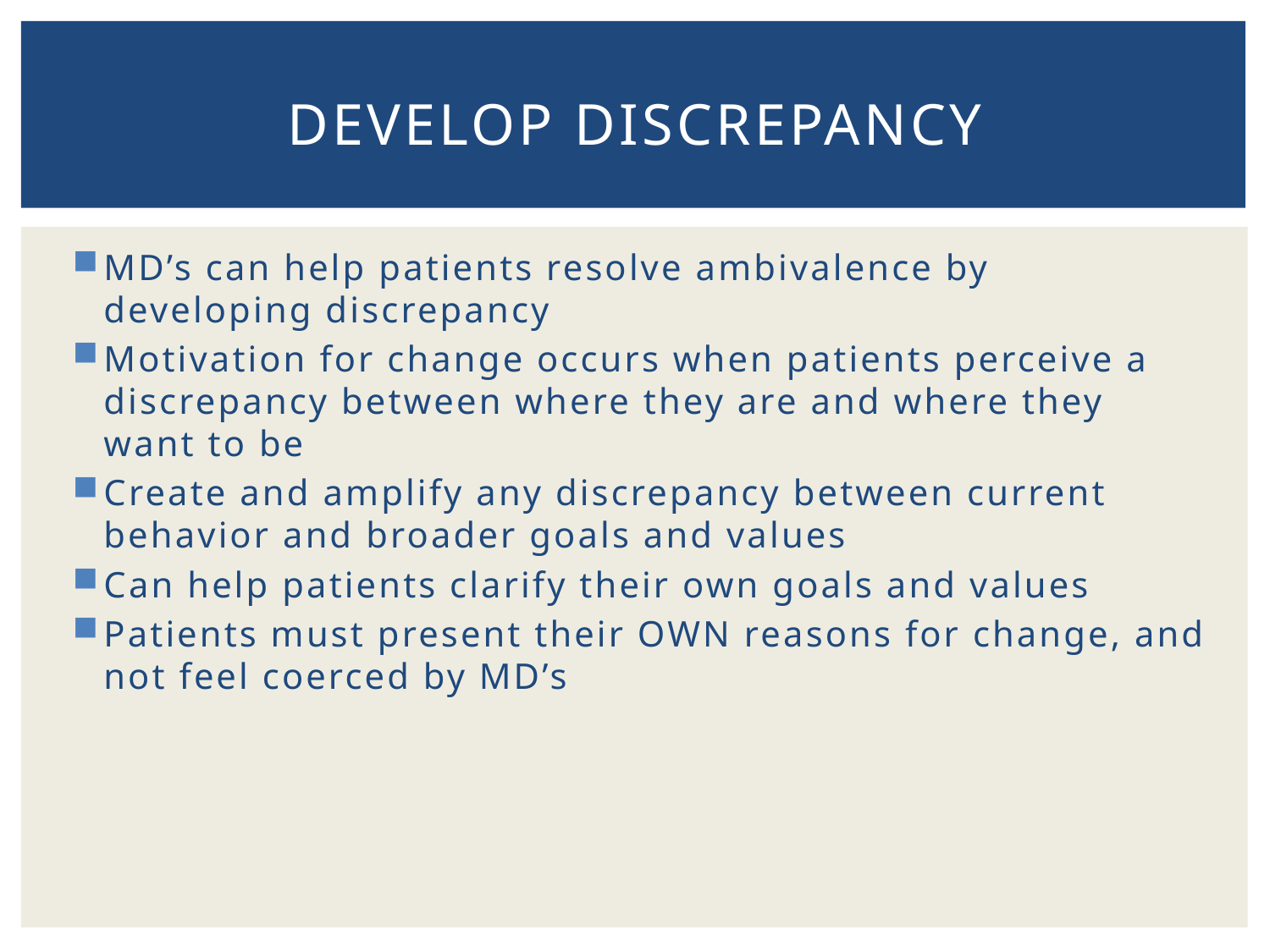

# Develop discrepancy
MD’s can help patients resolve ambivalence by developing discrepancy
Motivation for change occurs when patients perceive a discrepancy between where they are and where they want to be
Create and amplify any discrepancy between current behavior and broader goals and values
Can help patients clarify their own goals and values
Patients must present their OWN reasons for change, and not feel coerced by MD’s

## Slide 20
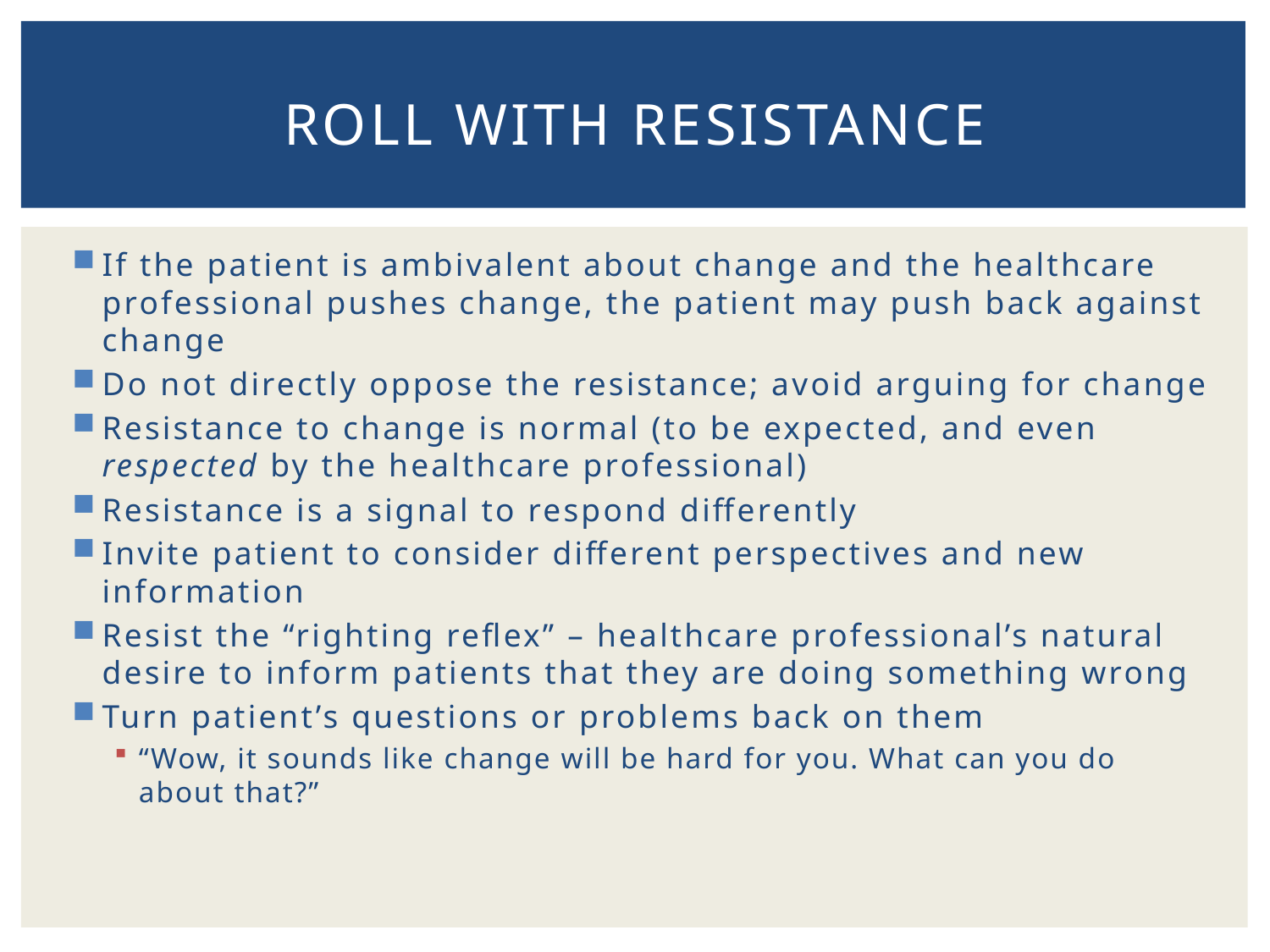

# Roll with resistance
If the patient is ambivalent about change and the healthcare professional pushes change, the patient may push back against change
Do not directly oppose the resistance; avoid arguing for change
Resistance to change is normal (to be expected, and even respected by the healthcare professional)
Resistance is a signal to respond differently
Invite patient to consider different perspectives and new information
Resist the “righting reflex” – healthcare professional’s natural desire to inform patients that they are doing something wrong
Turn patient’s questions or problems back on them
“Wow, it sounds like change will be hard for you. What can you do about that?”

## Slide 21
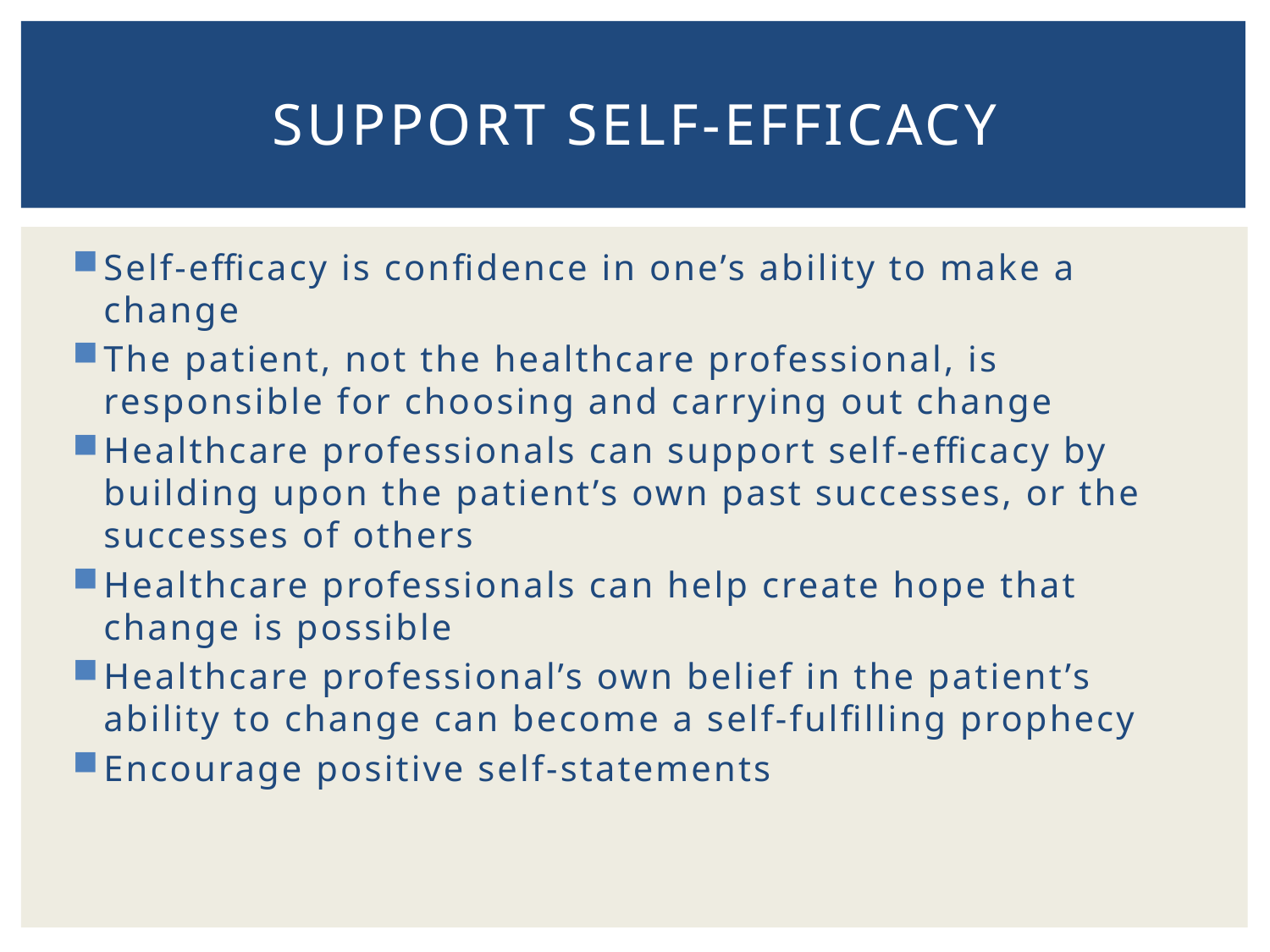

# Support self-efficacy
Self-efficacy is confidence in one’s ability to make a change
The patient, not the healthcare professional, is responsible for choosing and carrying out change
Healthcare professionals can support self-efficacy by building upon the patient’s own past successes, or the successes of others
Healthcare professionals can help create hope that change is possible
Healthcare professional’s own belief in the patient’s ability to change can become a self-fulfilling prophecy
Encourage positive self-statements

## Slide 22
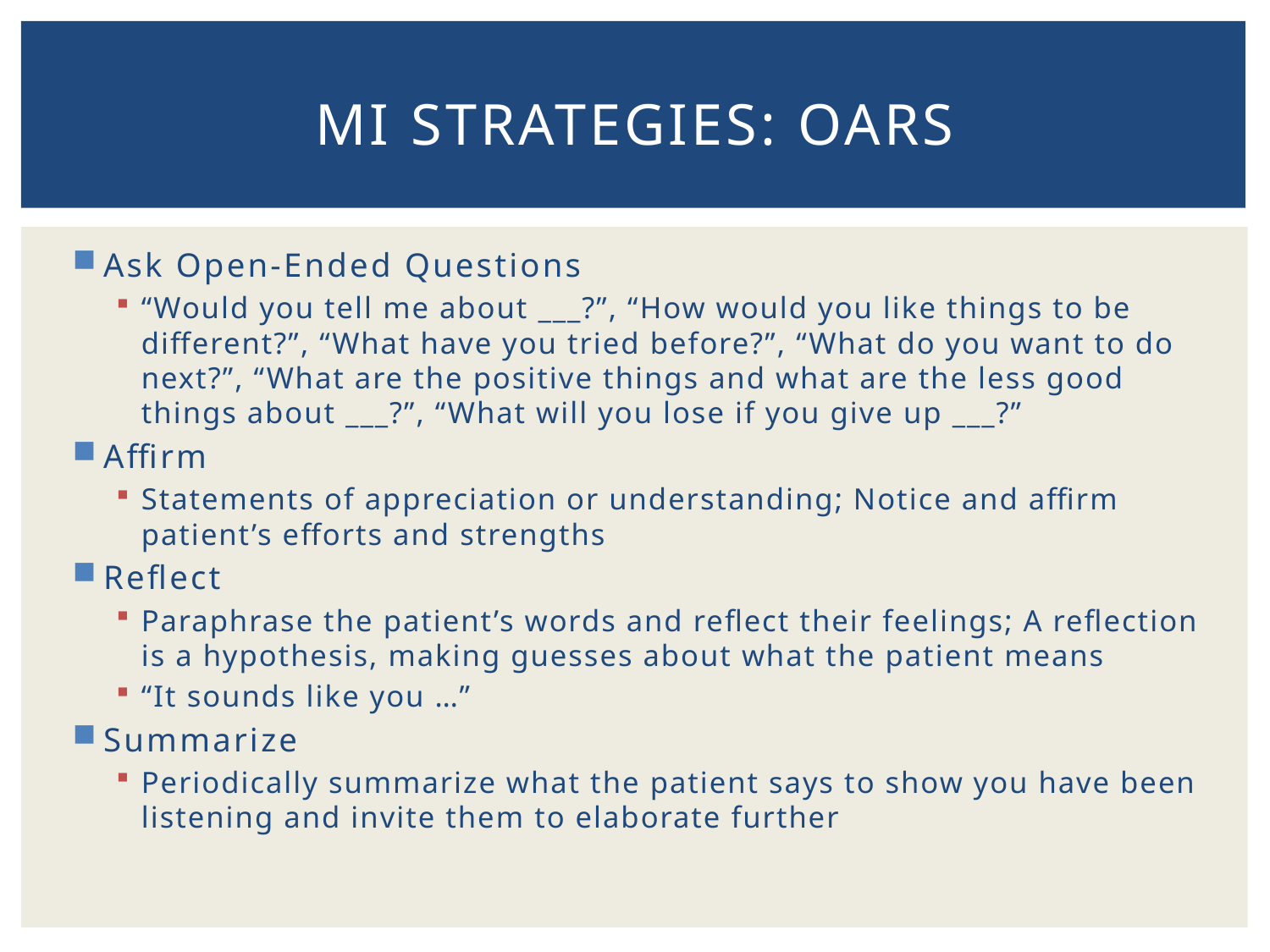

# MI Strategies: OARS
Ask Open-Ended Questions
“Would you tell me about ___?”, “How would you like things to be different?”, “What have you tried before?”, “What do you want to do next?”, “What are the positive things and what are the less good things about ___?”, “What will you lose if you give up ___?”
Affirm
Statements of appreciation or understanding; Notice and affirm patient’s efforts and strengths
Reflect
Paraphrase the patient’s words and reflect their feelings; A reflection is a hypothesis, making guesses about what the patient means
“It sounds like you …”
Summarize
Periodically summarize what the patient says to show you have been listening and invite them to elaborate further

## Slide 23
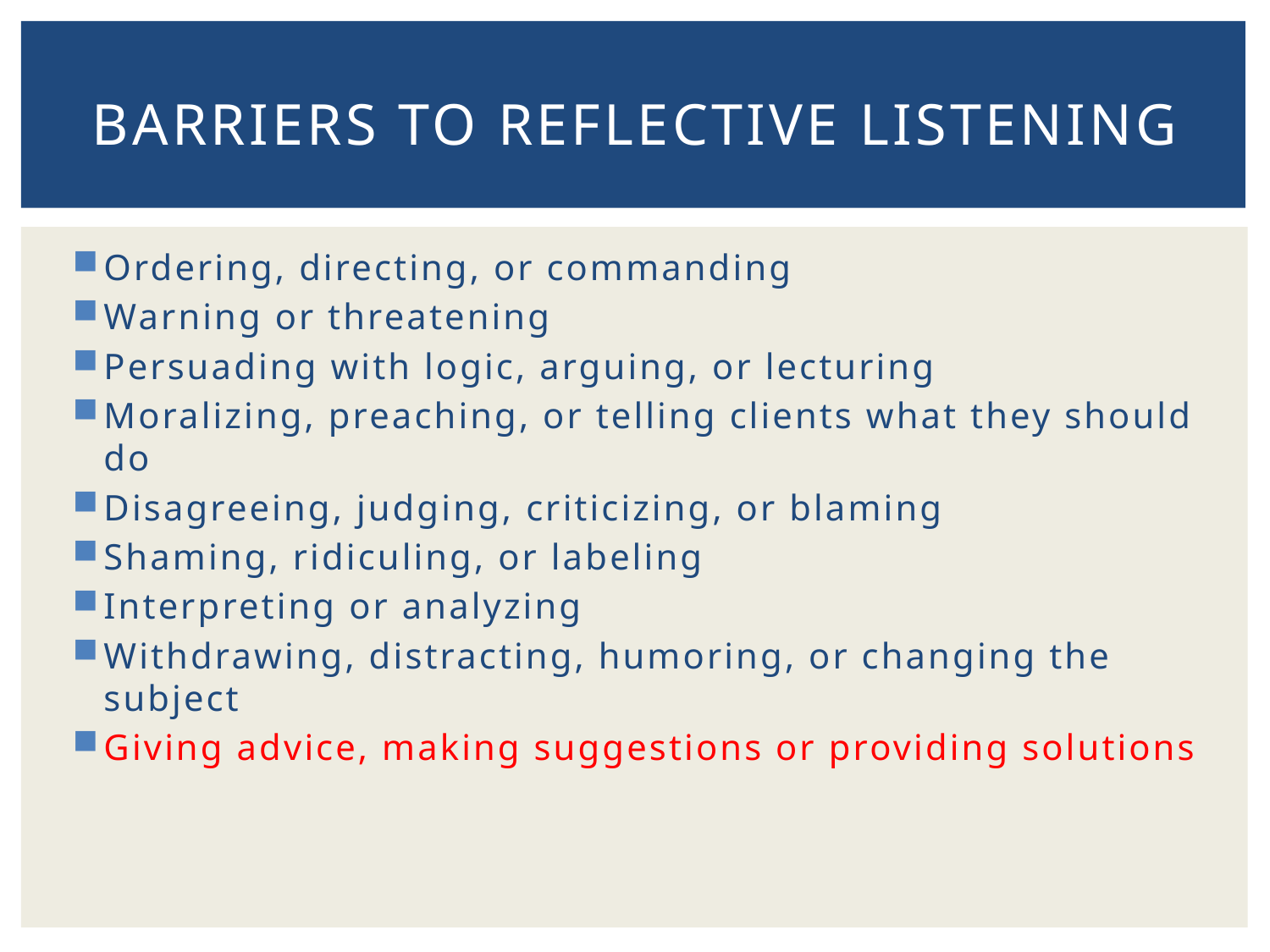

# Barriers to reflective listening
Ordering, directing, or commanding
Warning or threatening
Persuading with logic, arguing, or lecturing
Moralizing, preaching, or telling clients what they should do
Disagreeing, judging, criticizing, or blaming
Shaming, ridiculing, or labeling
Interpreting or analyzing
Withdrawing, distracting, humoring, or changing the subject
Giving advice, making suggestions or providing solutions

## Slide 24
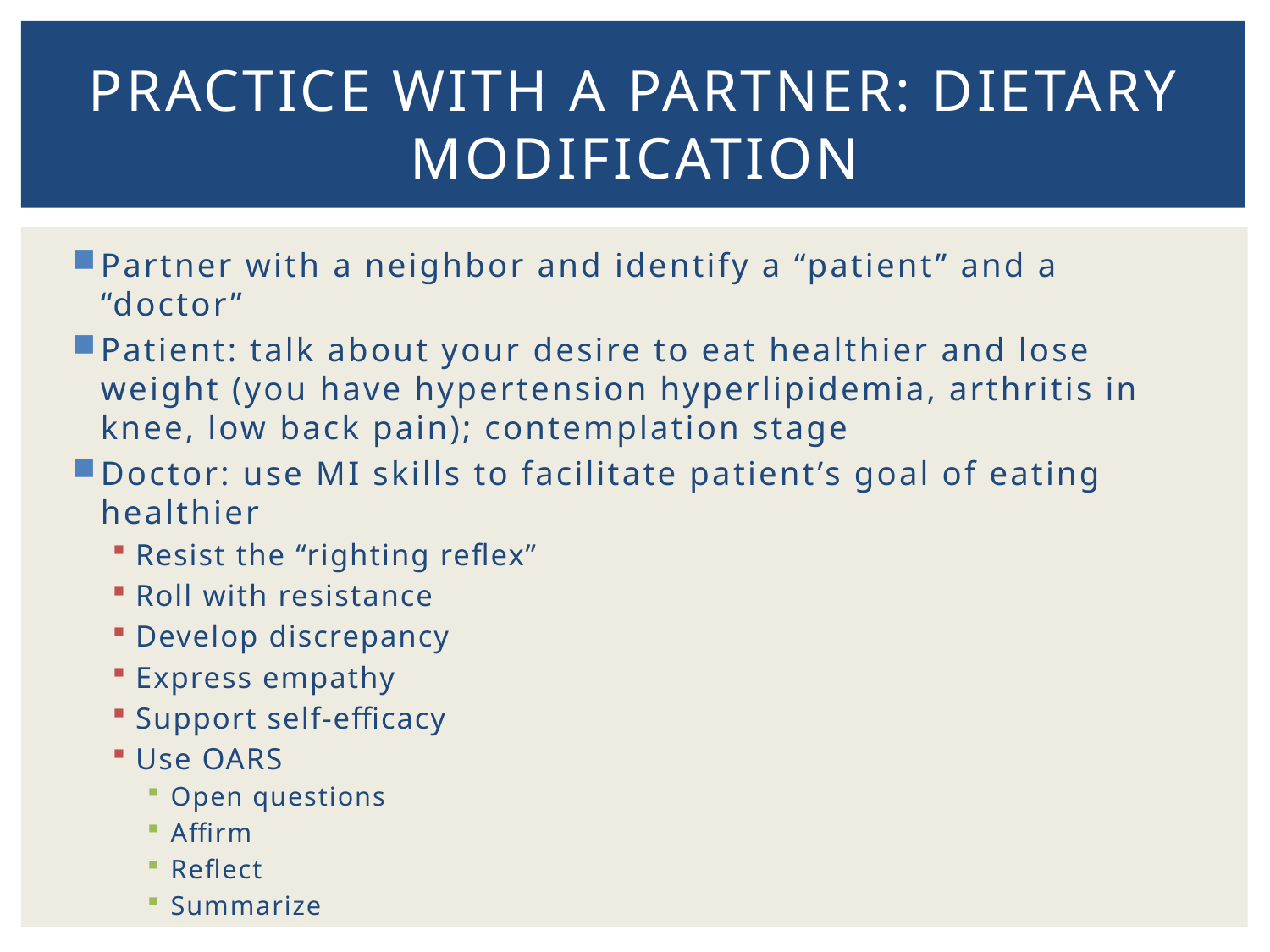

# Practice with a partner: Dietary Modification
Partner with a neighbor and identify a “patient” and a “doctor”
Patient: talk about your desire to eat healthier and lose weight (you have hypertension hyperlipidemia, arthritis in knee, low back pain); contemplation stage
Doctor: use MI skills to facilitate patient’s goal of eating healthier
Resist the “righting reflex”
Roll with resistance
Develop discrepancy
Express empathy
Support self-efficacy
Use OARS
Open questions
Affirm
Reflect
Summarize

## Slide 25
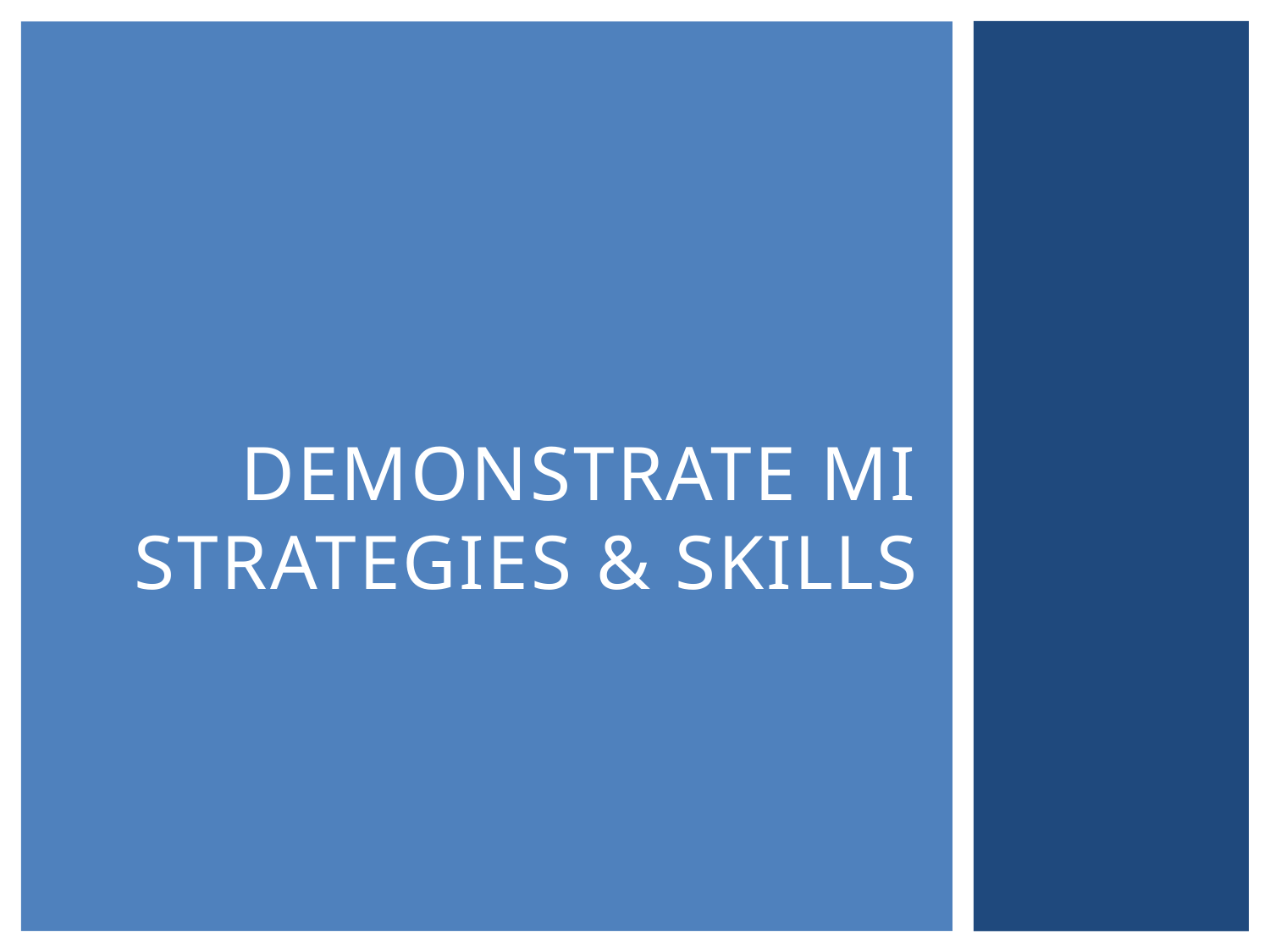

# Demonstrate MI strategies & skills

## Slide 26
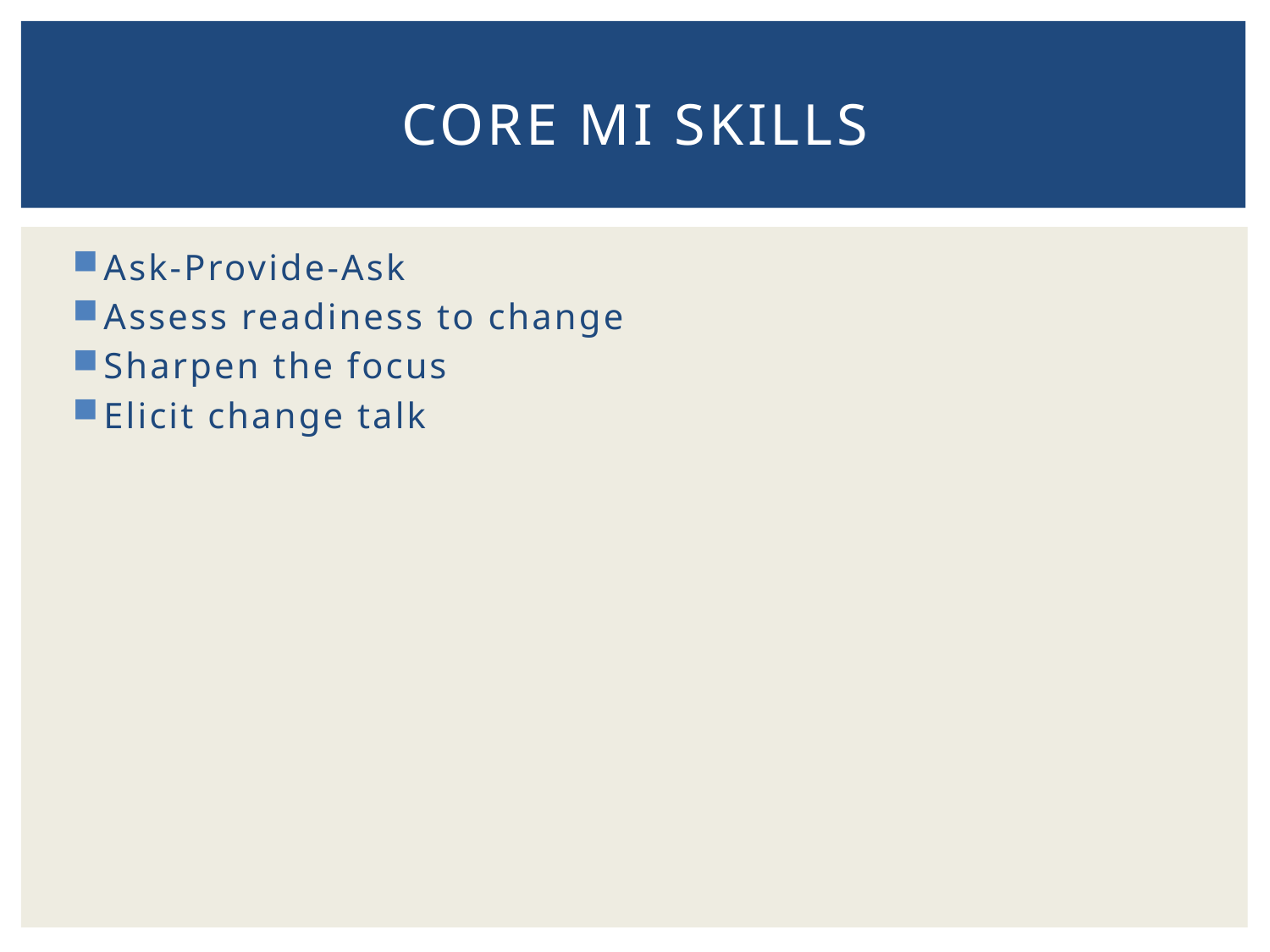

# Core MI skills
Ask-Provide-Ask
Assess readiness to change
Sharpen the focus
Elicit change talk

## Slide 27
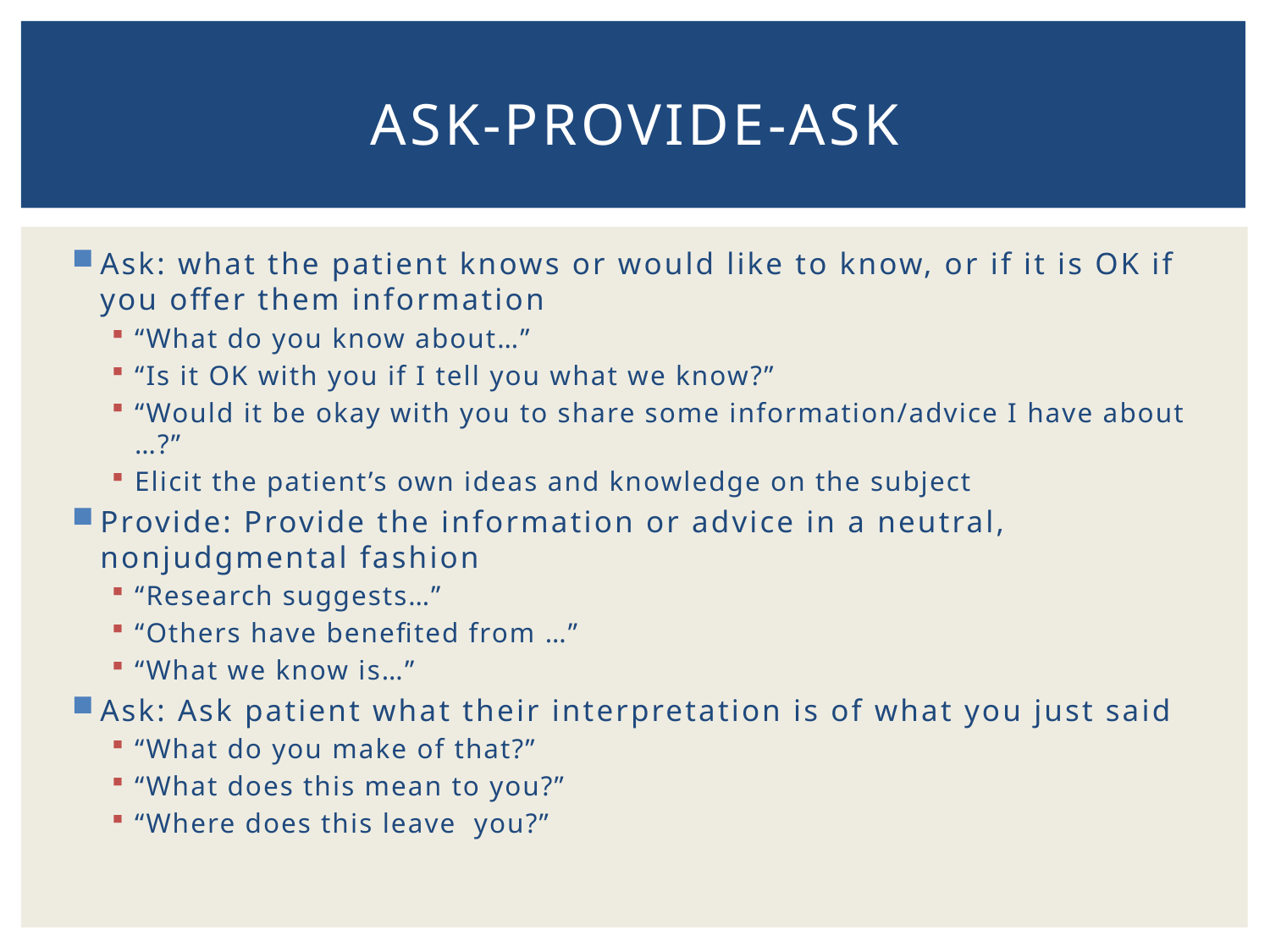

# Ask-provide-ask
Ask: what the patient knows or would like to know, or if it is OK if you offer them information
“What do you know about…”
“Is it OK with you if I tell you what we know?”
“Would it be okay with you to share some information/advice I have about …?”
Elicit the patient’s own ideas and knowledge on the subject
Provide: Provide the information or advice in a neutral, nonjudgmental fashion
“Research suggests…”
“Others have benefited from …”
“What we know is…”
Ask: Ask patient what their interpretation is of what you just said
“What do you make of that?”
“What does this mean to you?”
“Where does this leave you?”

## Slide 28
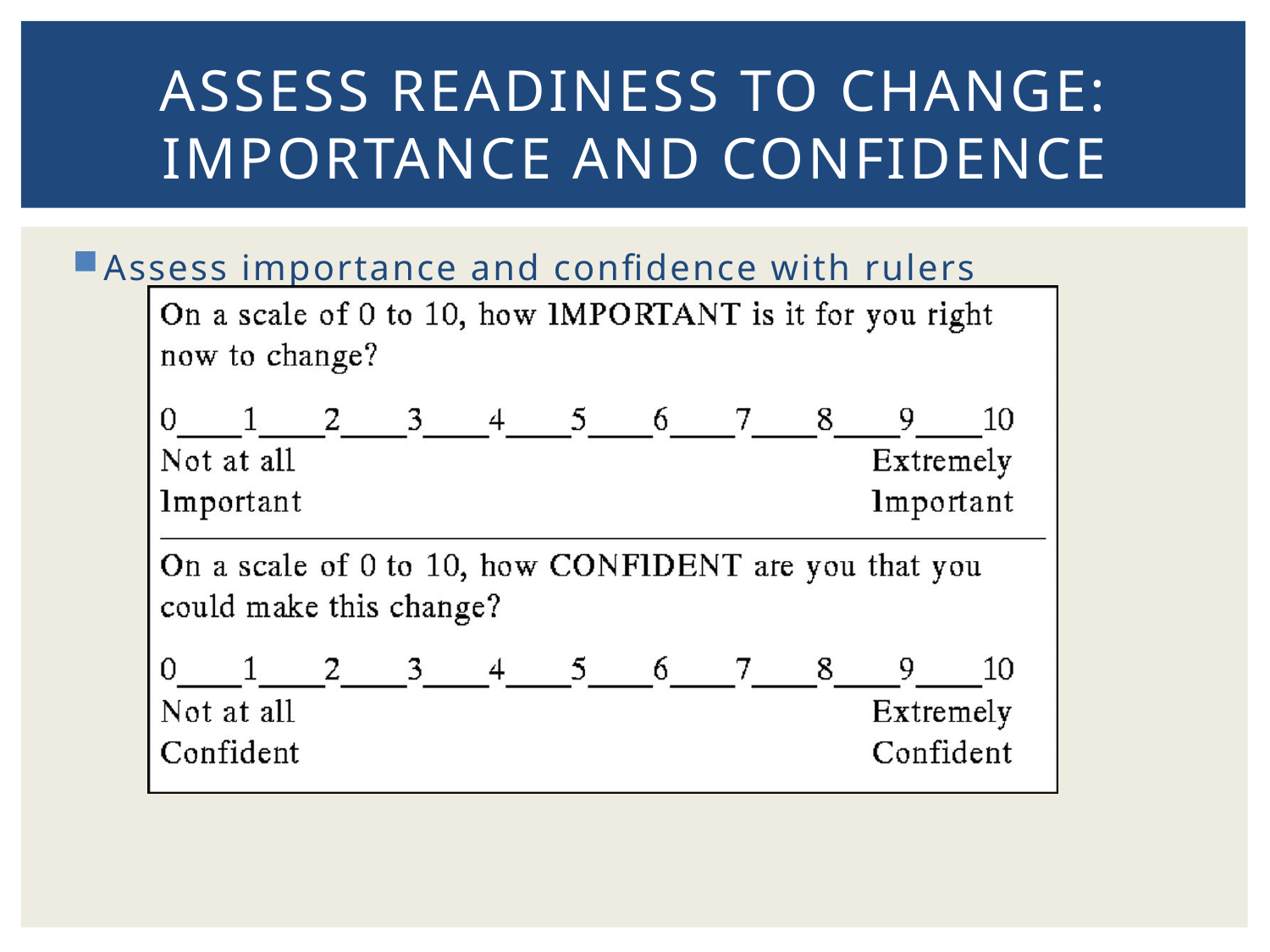

# Assess Readiness to Change: Importance and Confidence
Assess importance and confidence with rulers

## Slide 29
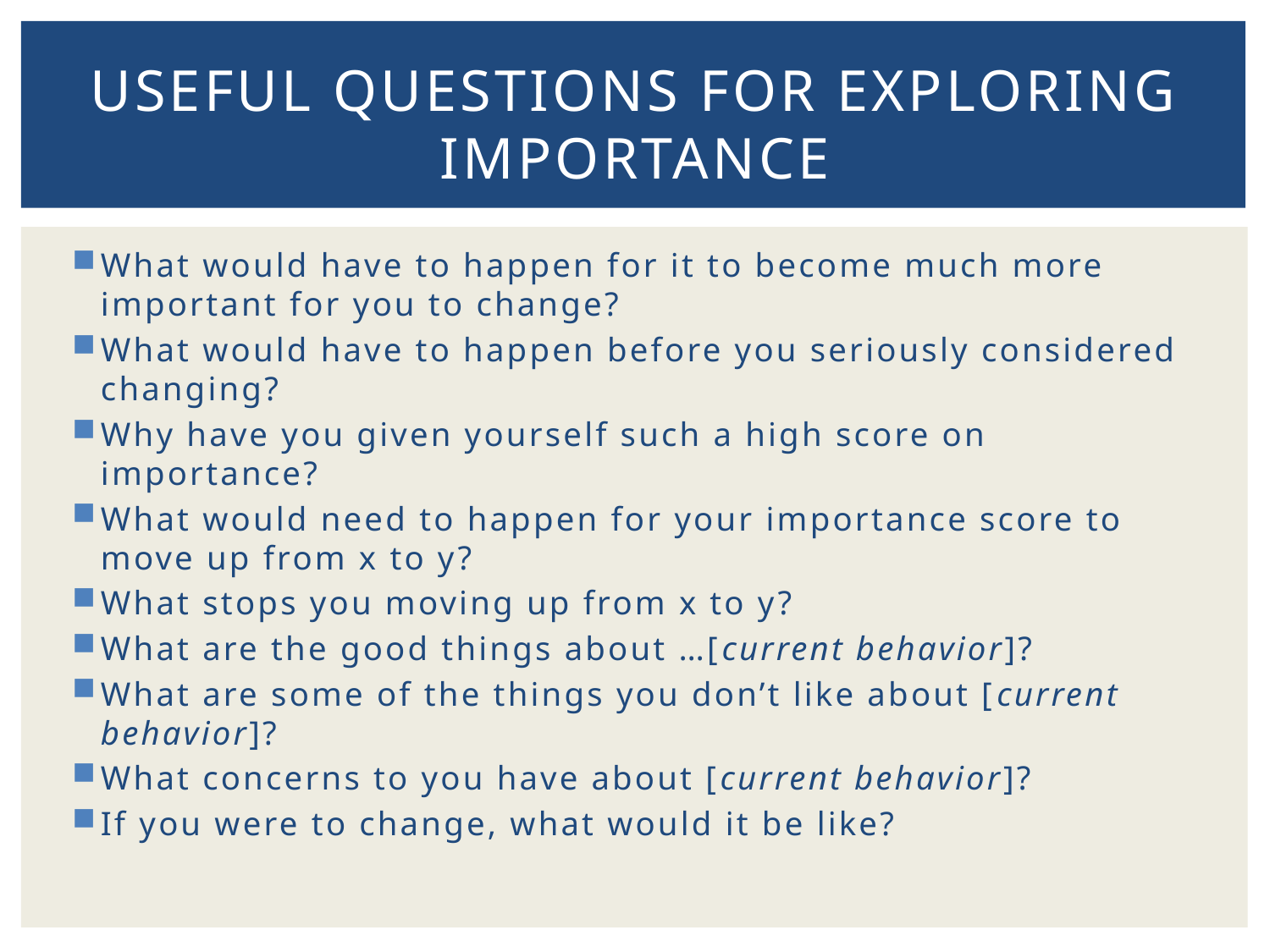

# Useful Questions for exploring importance
What would have to happen for it to become much more important for you to change?
What would have to happen before you seriously considered changing?
Why have you given yourself such a high score on importance?
What would need to happen for your importance score to move up from x to y?
What stops you moving up from x to y?
What are the good things about …[current behavior]?
What are some of the things you don’t like about [current behavior]?
What concerns to you have about [current behavior]?
If you were to change, what would it be like?

## Slide 30
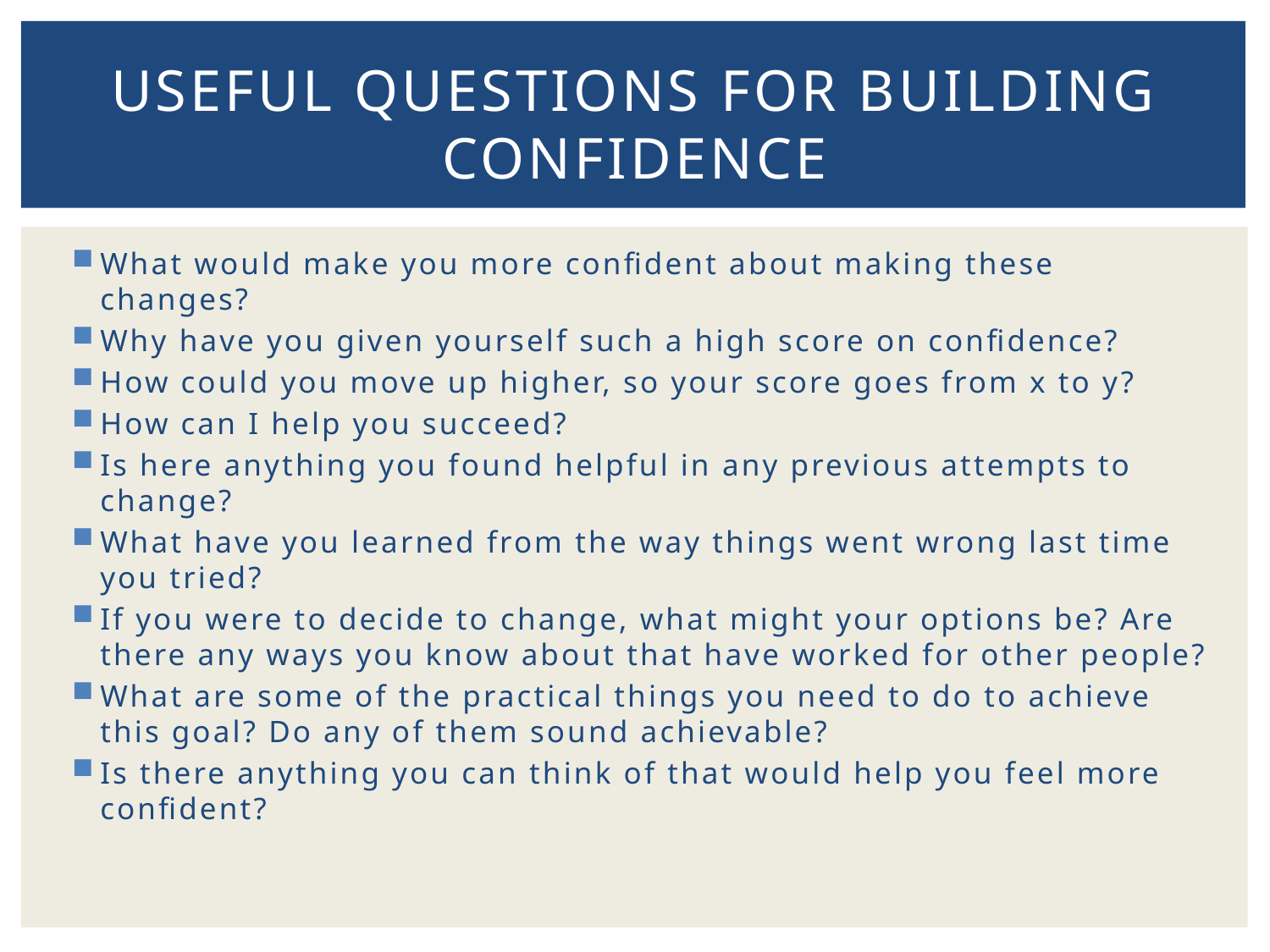

# Useful questions for building confidence
What would make you more confident about making these changes?
Why have you given yourself such a high score on confidence?
How could you move up higher, so your score goes from x to y?
How can I help you succeed?
Is here anything you found helpful in any previous attempts to change?
What have you learned from the way things went wrong last time you tried?
If you were to decide to change, what might your options be? Are there any ways you know about that have worked for other people?
What are some of the practical things you need to do to achieve this goal? Do any of them sound achievable?
Is there anything you can think of that would help you feel more confident?

## Slide 31
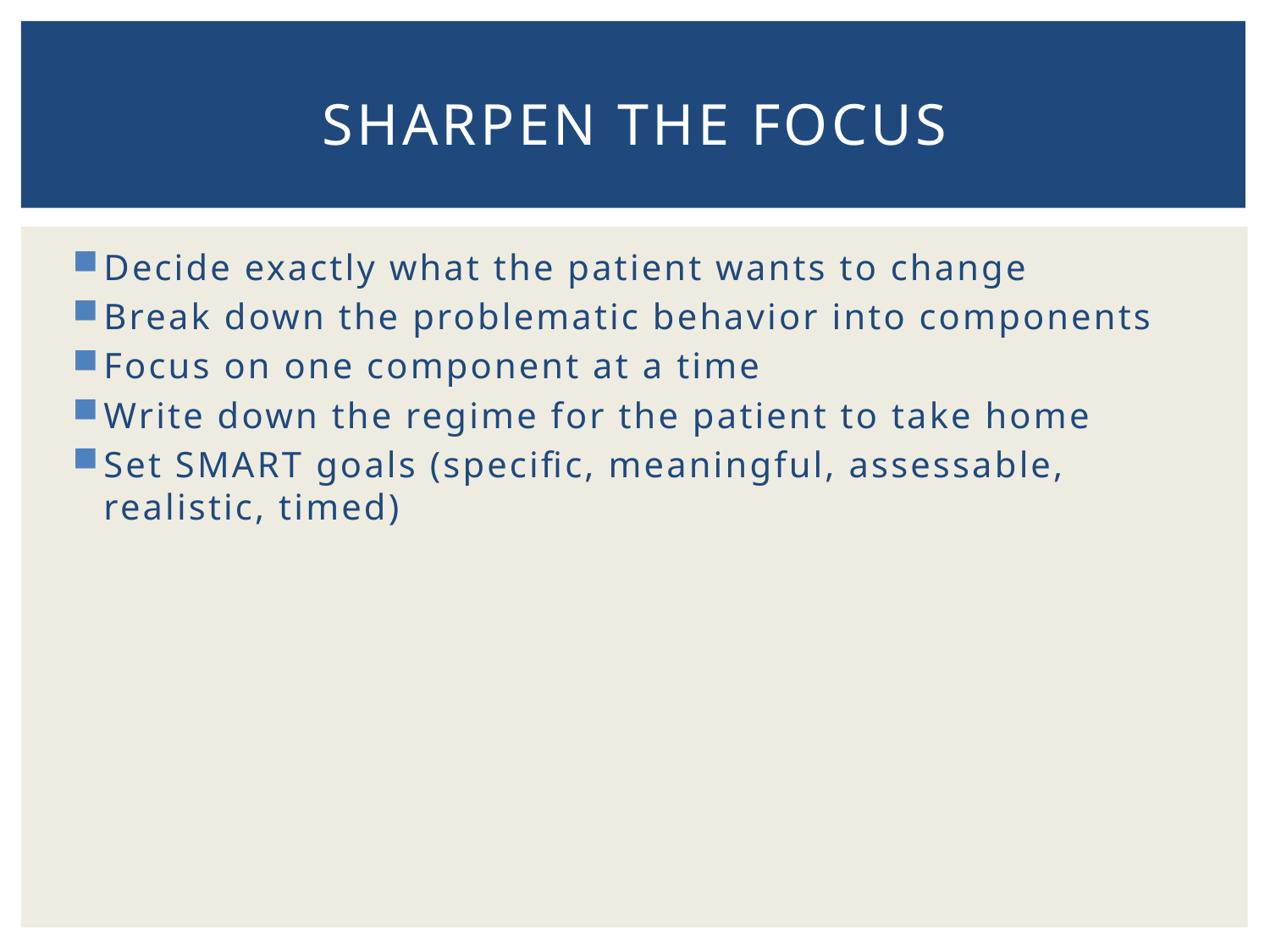

# Sharpen the focus
Decide exactly what the patient wants to change
Break down the problematic behavior into components
Focus on one component at a time
Write down the regime for the patient to take home
Set SMART goals (specific, meaningful, assessable, realistic, timed)

## Slide 32
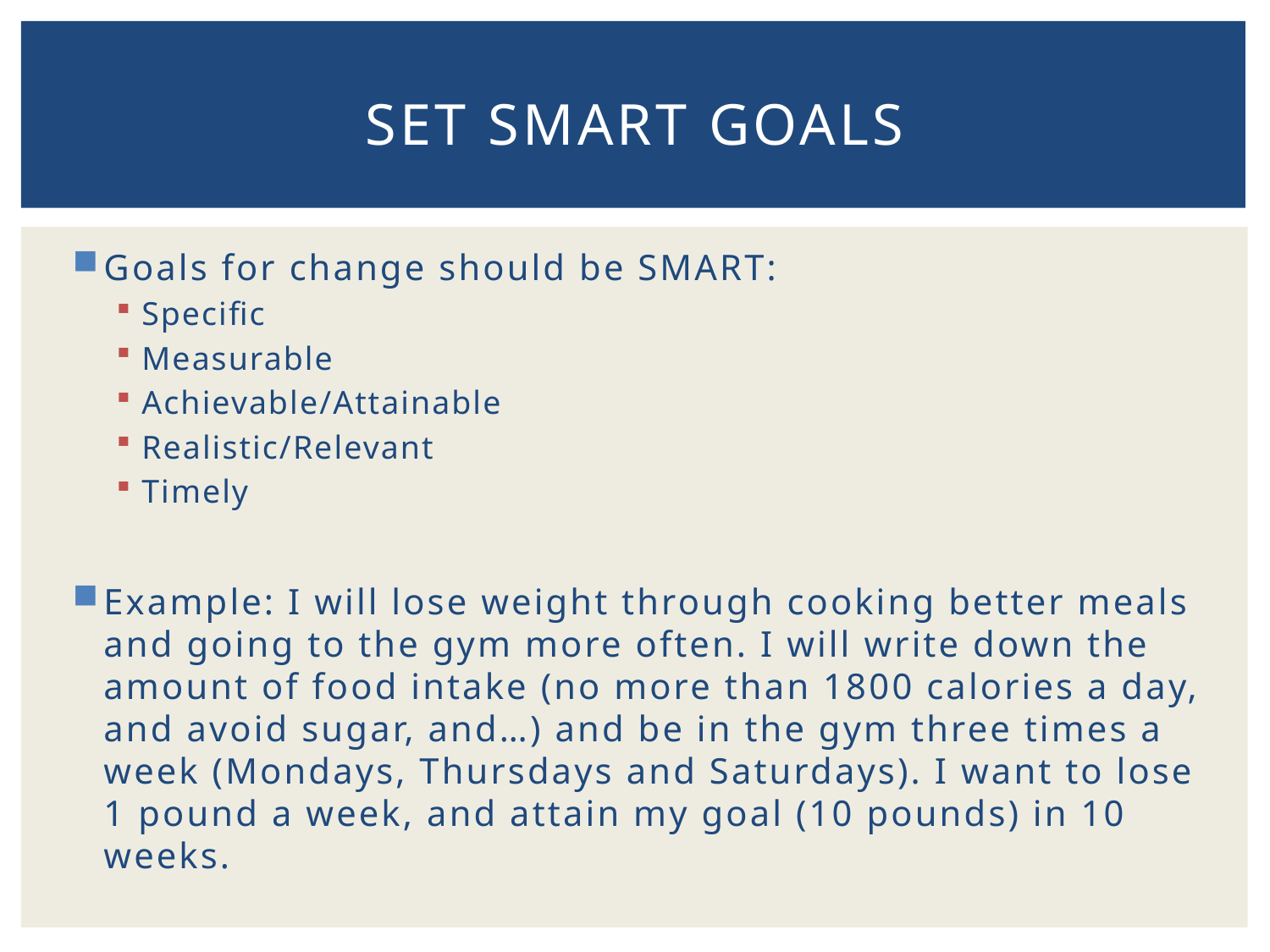

# Set SMART Goals
Goals for change should be SMART:
Specific
Measurable
Achievable/Attainable
Realistic/Relevant
Timely
Example: I will lose weight through cooking better meals and going to the gym more often. I will write down the amount of food intake (no more than 1800 calories a day, and avoid sugar, and…) and be in the gym three times a week (Mondays, Thursdays and Saturdays). I want to lose 1 pound a week, and attain my goal (10 pounds) in 10 weeks.

## Slide 33
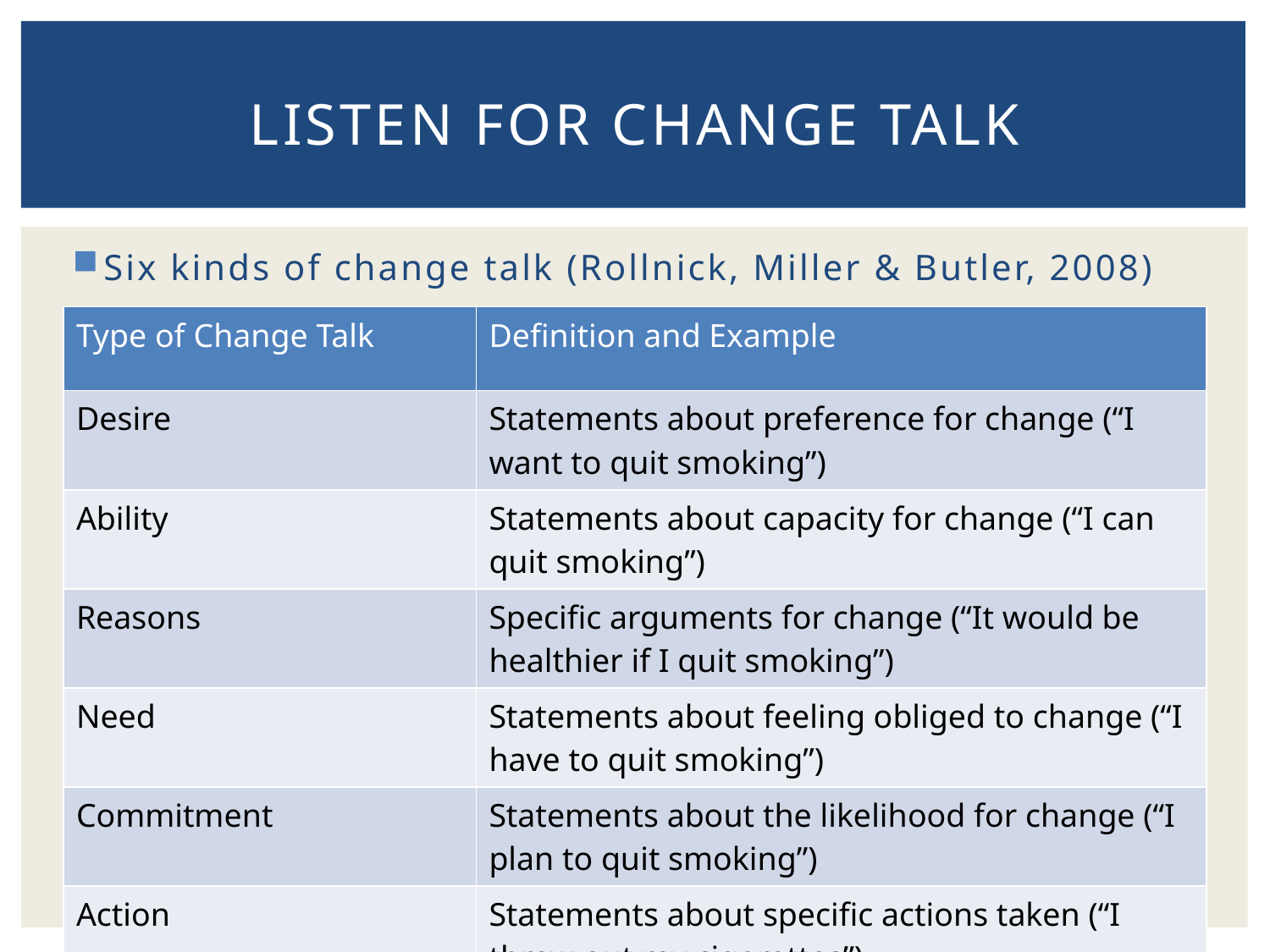

# Listen for change talk
Six kinds of change talk (Rollnick, Miller & Butler, 2008)
| Type of Change Talk | Definition and Example |
| --- | --- |
| Desire | Statements about preference for change (“I want to quit smoking”) |
| Ability | Statements about capacity for change (“I can quit smoking”) |
| Reasons | Specific arguments for change (“It would be healthier if I quit smoking”) |
| Need | Statements about feeling obliged to change (“I have to quit smoking”) |
| Commitment | Statements about the likelihood for change (“I plan to quit smoking”) |
| Action | Statements about specific actions taken (“I threw out my cigarettes”) |

## Slide 34
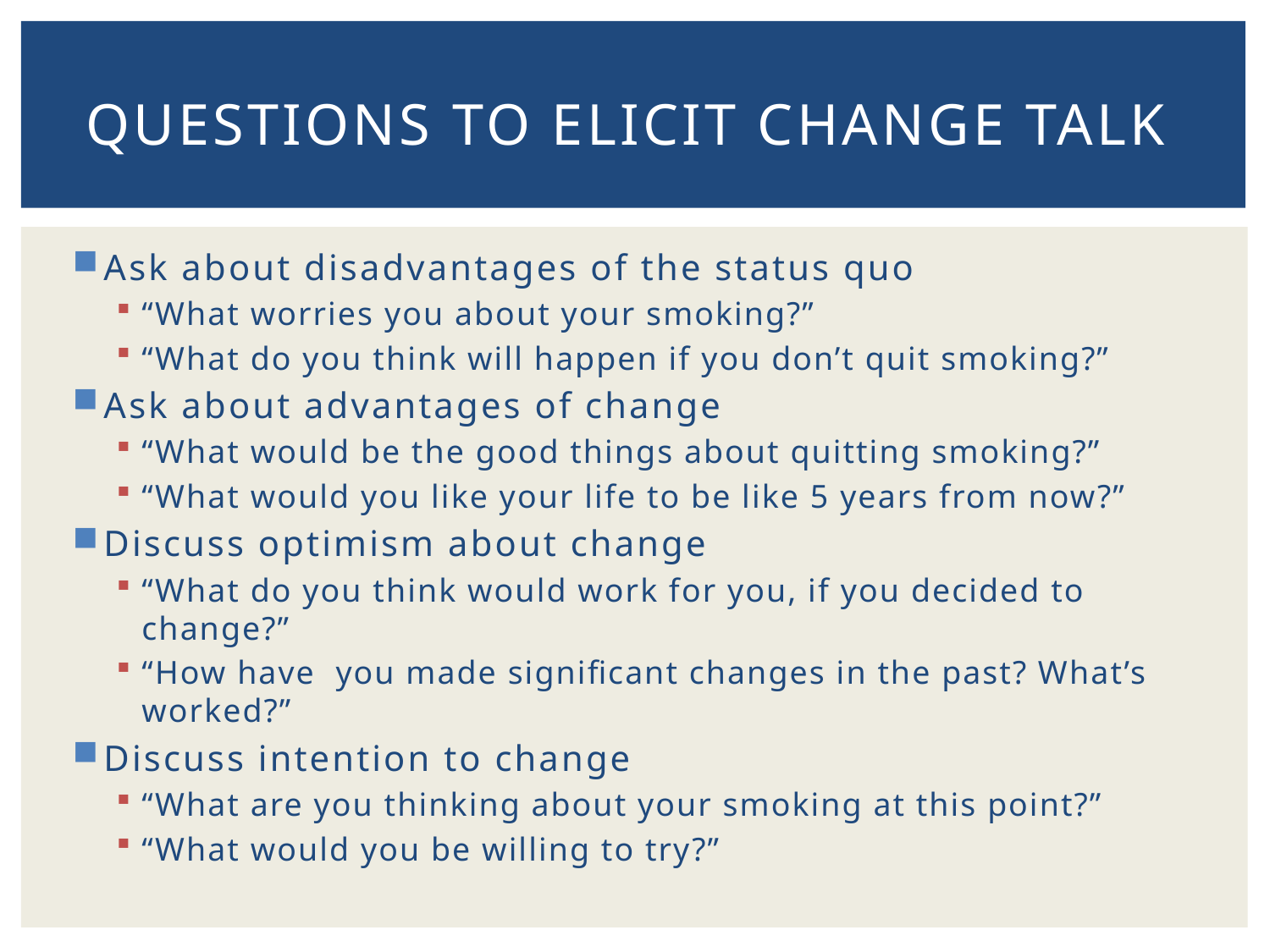

# Questions to elicit change talk
Ask about disadvantages of the status quo
“What worries you about your smoking?”
“What do you think will happen if you don’t quit smoking?”
Ask about advantages of change
“What would be the good things about quitting smoking?”
“What would you like your life to be like 5 years from now?”
Discuss optimism about change
“What do you think would work for you, if you decided to change?”
“How have you made significant changes in the past? What’s worked?”
Discuss intention to change
“What are you thinking about your smoking at this point?”
“What would you be willing to try?”

## Slide 35
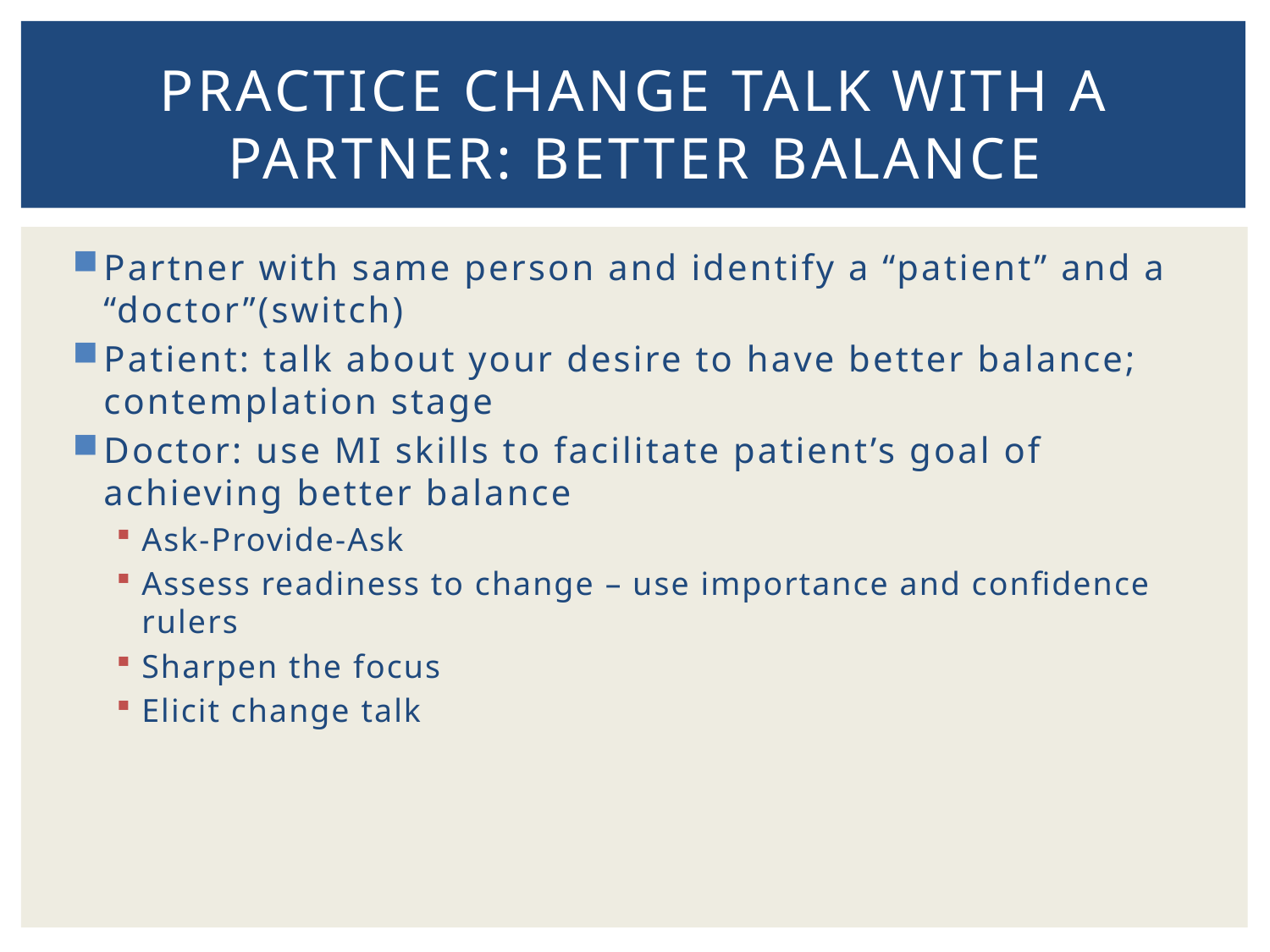

# Practice Change Talk with a partner: Better Balance
Partner with same person and identify a “patient” and a “doctor”(switch)
Patient: talk about your desire to have better balance; contemplation stage
Doctor: use MI skills to facilitate patient’s goal of achieving better balance
Ask-Provide-Ask
Assess readiness to change – use importance and confidence rulers
Sharpen the focus
Elicit change talk

## Slide 36
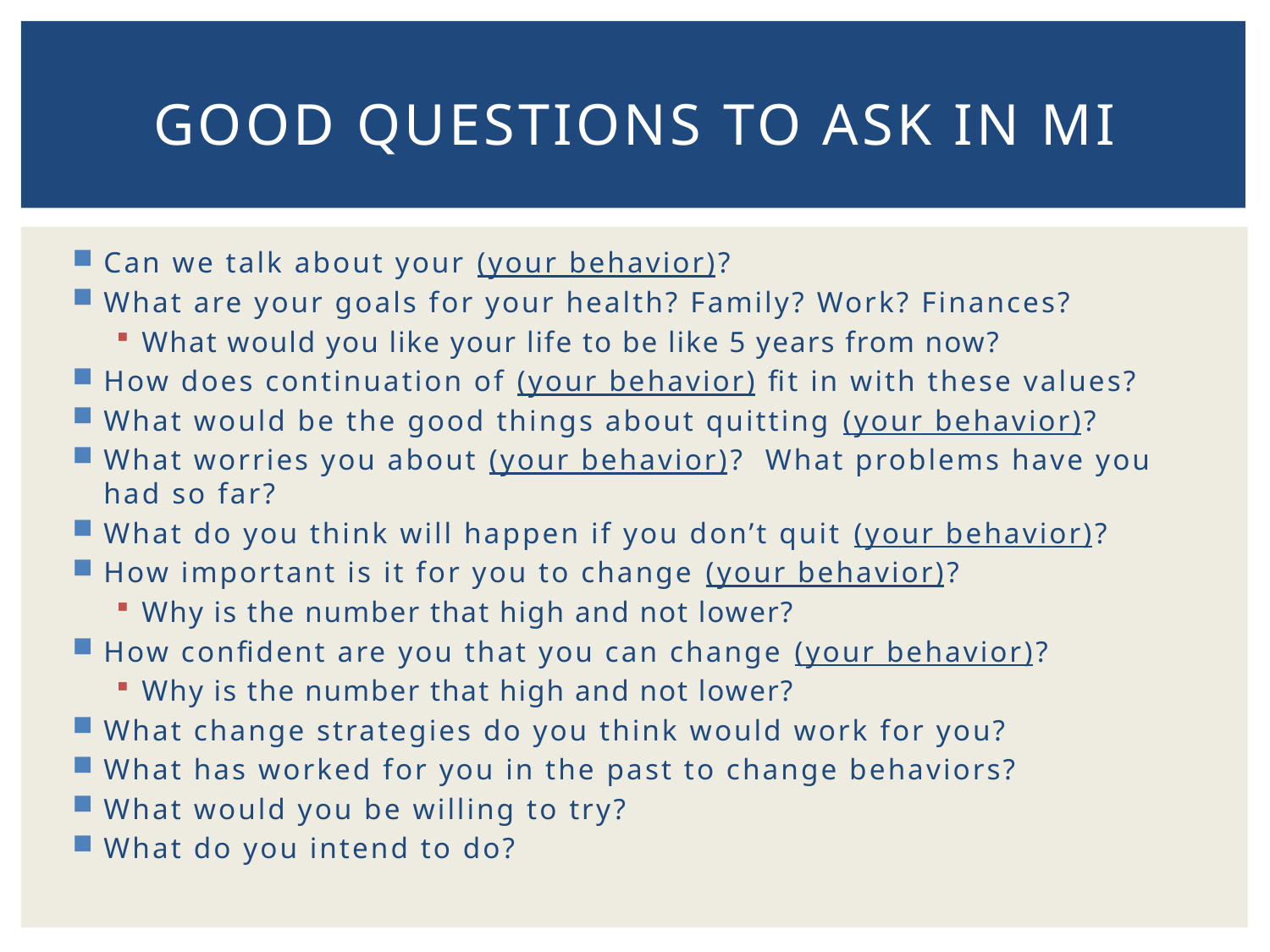

# Good questions to ask in mi
Can we talk about your (your behavior)?
What are your goals for your health? Family? Work? Finances?
What would you like your life to be like 5 years from now?
How does continuation of (your behavior) fit in with these values?
What would be the good things about quitting (your behavior)?
What worries you about (your behavior)? What problems have you had so far?
What do you think will happen if you don’t quit (your behavior)?
How important is it for you to change (your behavior)?
Why is the number that high and not lower?
How confident are you that you can change (your behavior)?
Why is the number that high and not lower?
What change strategies do you think would work for you?
What has worked for you in the past to change behaviors?
What would you be willing to try?
What do you intend to do?

## Slide 37
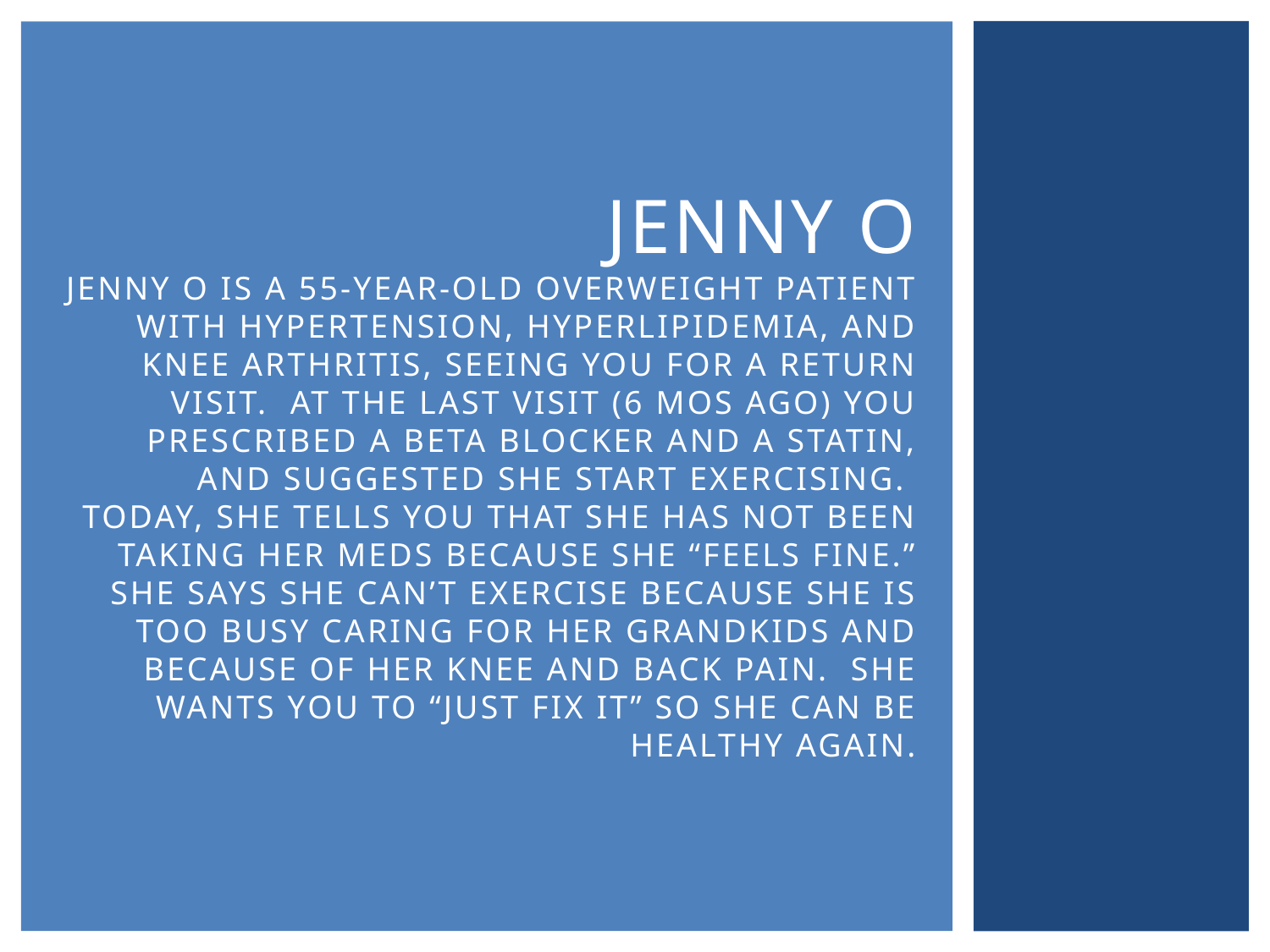

# Jenny o Jenny O is a 55-year-old overweight patient with hypertension, hyperlipidemia, and knee arthritis, seeing you for a return visit. At the last visit (6 mos ago) you prescribed a beta blocker and a statin, and suggested she start exercising. Today, she tells you that she has not been taking her meds because she “feels fine.” She says she can’t exercise because she is too busy caring for her grandkids and because of her knee and back pain. She wants you to “just fix it” so she can be healthy again.

## Slide 38
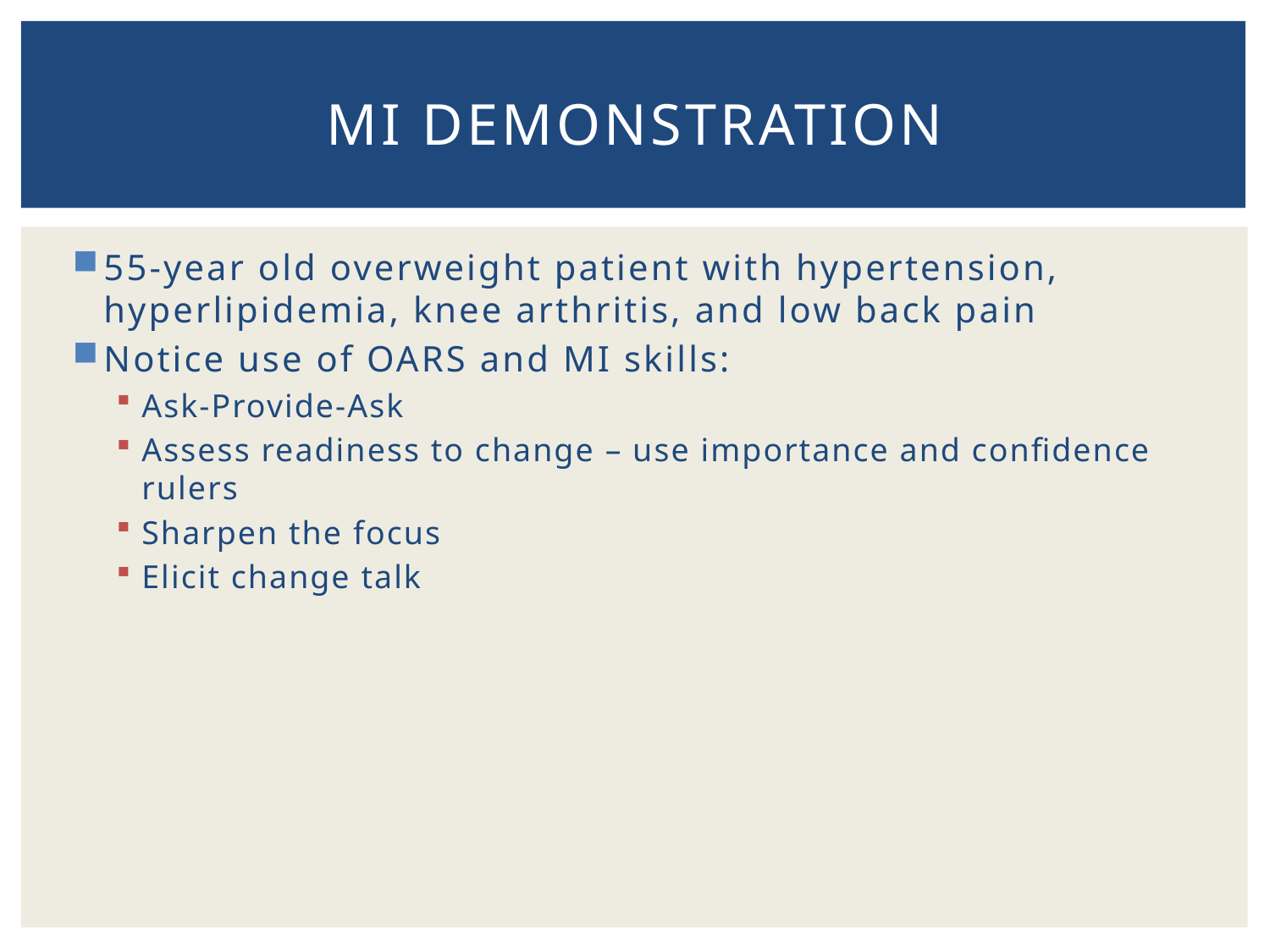

# MI demonstration
55-year old overweight patient with hypertension, hyperlipidemia, knee arthritis, and low back pain
Notice use of OARS and MI skills:
Ask-Provide-Ask
Assess readiness to change – use importance and confidence rulers
Sharpen the focus
Elicit change talk

## Slide 39
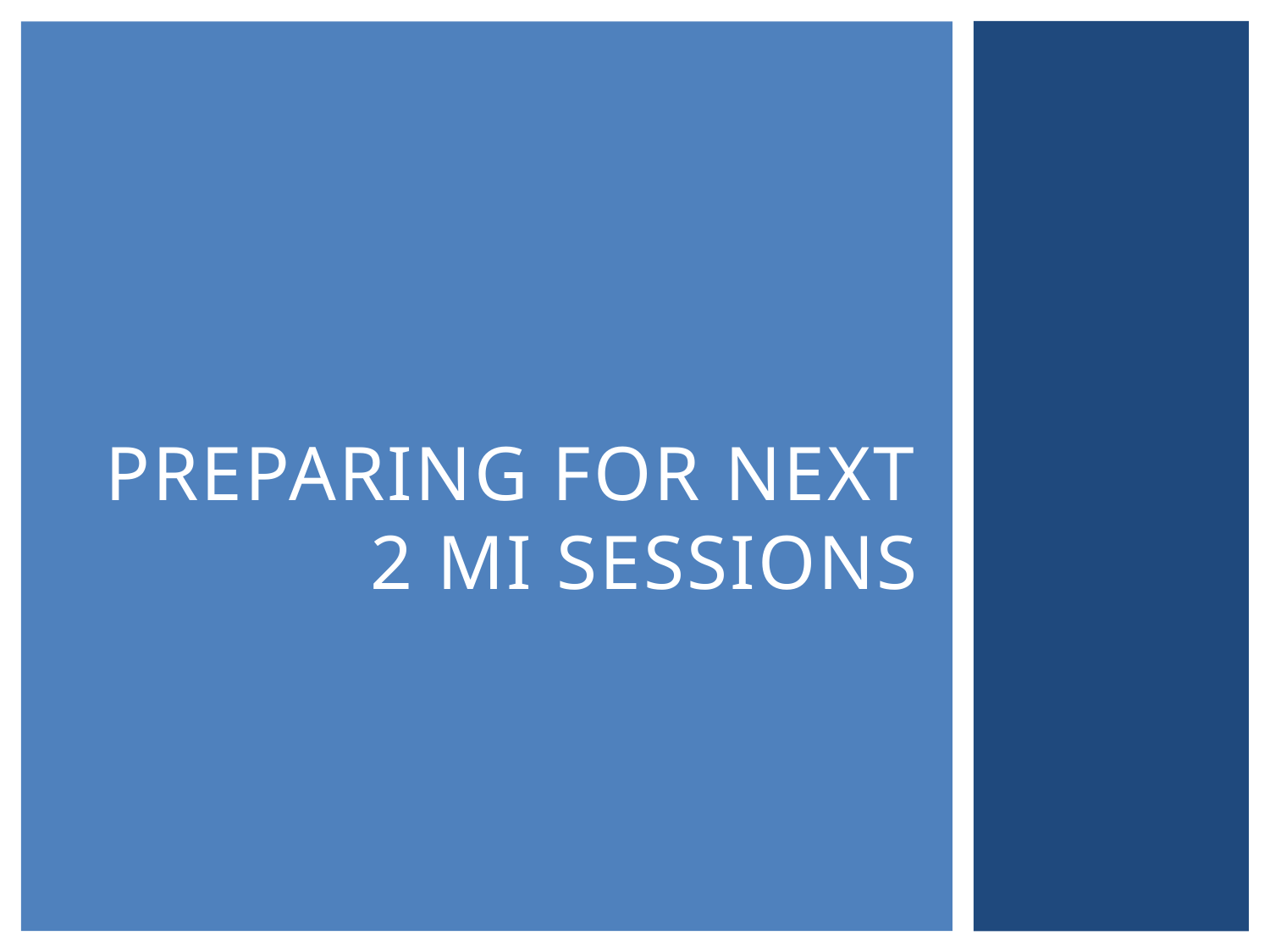

# Preparing for next 2 MI sessions

## Slide 40
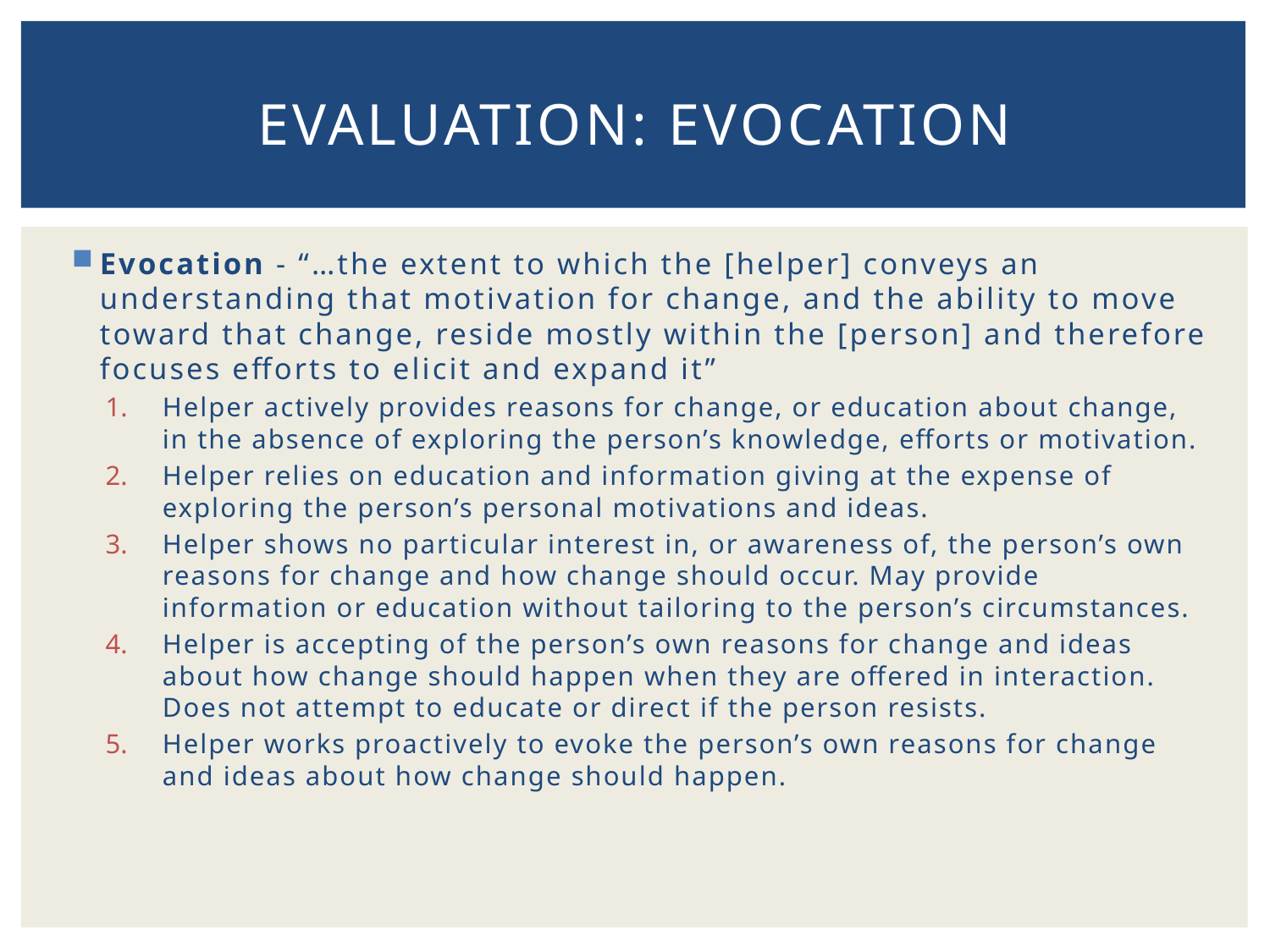

# Evaluation: Evocation
Evocation - “…the extent to which the [helper] conveys an understanding that motivation for change, and the ability to move toward that change, reside mostly within the [person] and therefore focuses efforts to elicit and expand it”
Helper actively provides reasons for change, or education about change, in the absence of exploring the person’s knowledge, efforts or motivation.
Helper relies on education and information giving at the expense of exploring the person’s personal motivations and ideas.
Helper shows no particular interest in, or awareness of, the person’s own reasons for change and how change should occur. May provide information or education without tailoring to the person’s circumstances.
Helper is accepting of the person’s own reasons for change and ideas about how change should happen when they are offered in interaction. Does not attempt to educate or direct if the person resists.
Helper works proactively to evoke the person’s own reasons for change and ideas about how change should happen.

## Slide 41
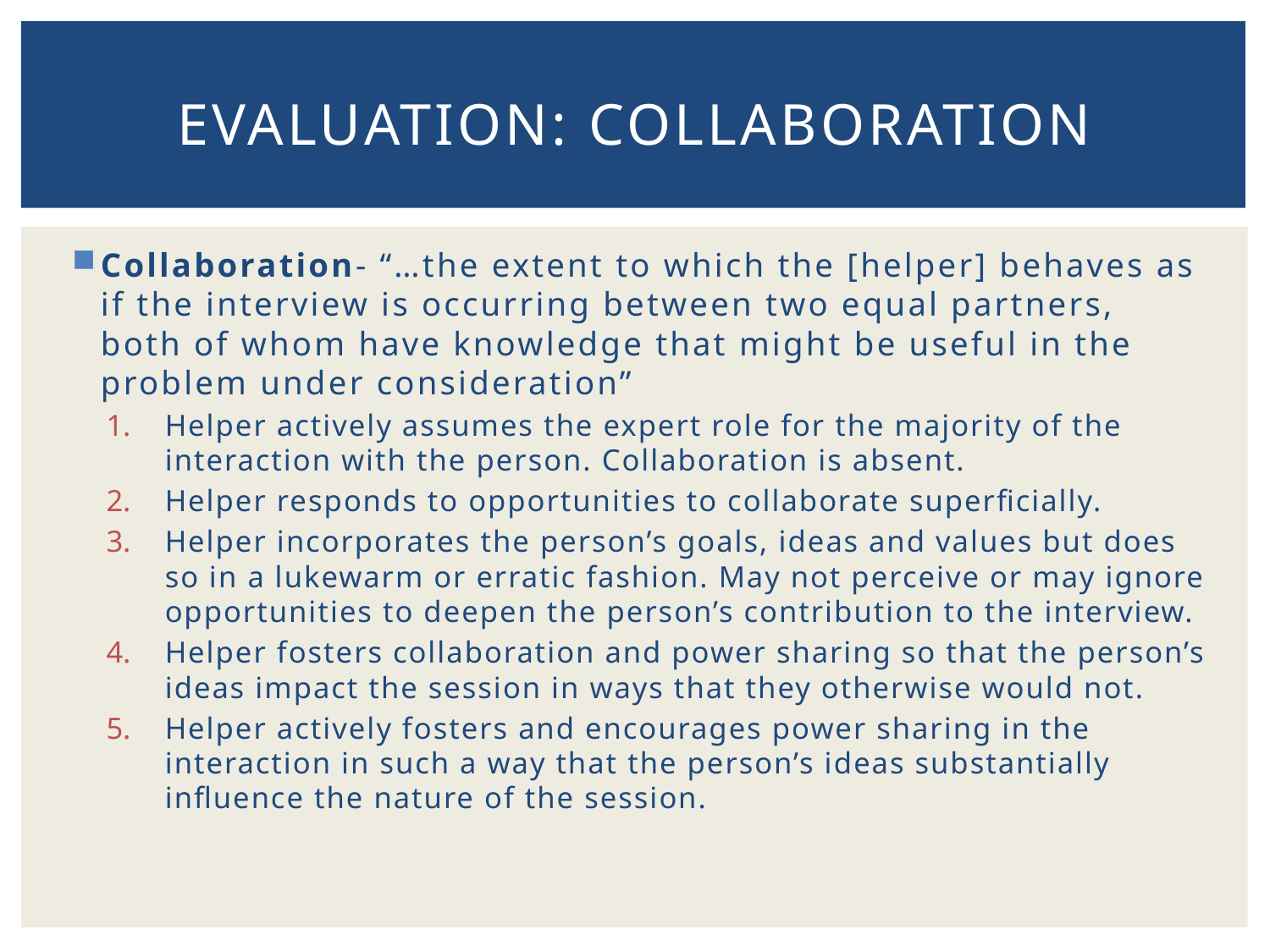

# Evaluation: collaboration
Collaboration- “…the extent to which the [helper] behaves as if the interview is occurring between two equal partners, both of whom have knowledge that might be useful in the problem under consideration”
Helper actively assumes the expert role for the majority of the interaction with the person. Collaboration is absent.
Helper responds to opportunities to collaborate superficially.
Helper incorporates the person’s goals, ideas and values but does so in a lukewarm or erratic fashion. May not perceive or may ignore opportunities to deepen the person’s contribution to the interview.
Helper fosters collaboration and power sharing so that the person’s ideas impact the session in ways that they otherwise would not.
Helper actively fosters and encourages power sharing in the interaction in such a way that the person’s ideas substantially influence the nature of the session.

## Slide 42
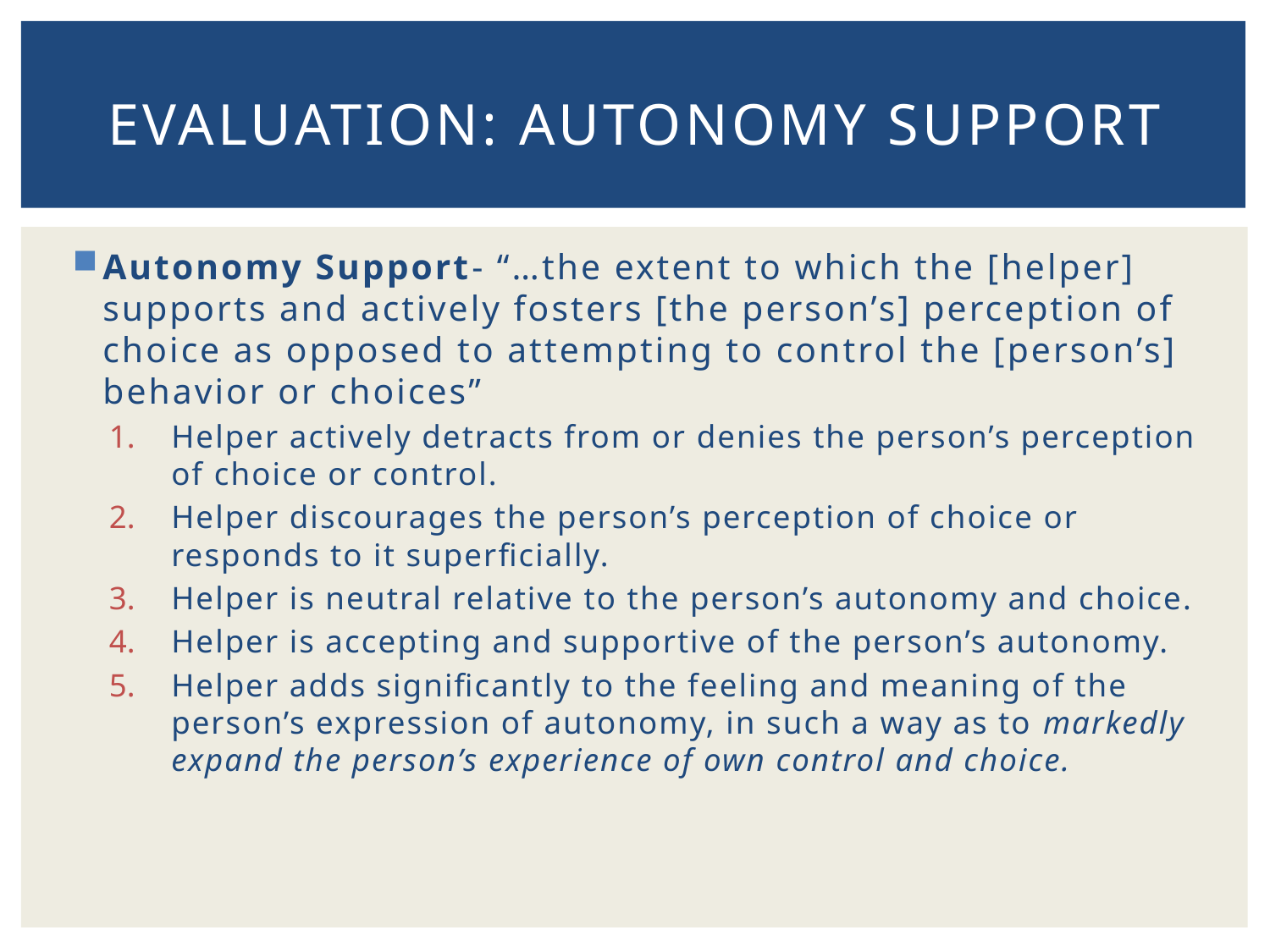

# Evaluation: autonomy support
Autonomy Support- “…the extent to which the [helper] supports and actively fosters [the person’s] perception of choice as opposed to attempting to control the [person’s] behavior or choices”
Helper actively detracts from or denies the person’s perception of choice or control.
Helper discourages the person’s perception of choice or responds to it superficially.
Helper is neutral relative to the person’s autonomy and choice.
Helper is accepting and supportive of the person’s autonomy.
Helper adds significantly to the feeling and meaning of the person’s expression of autonomy, in such a way as to markedly expand the person’s experience of own control and choice.

## Slide 43
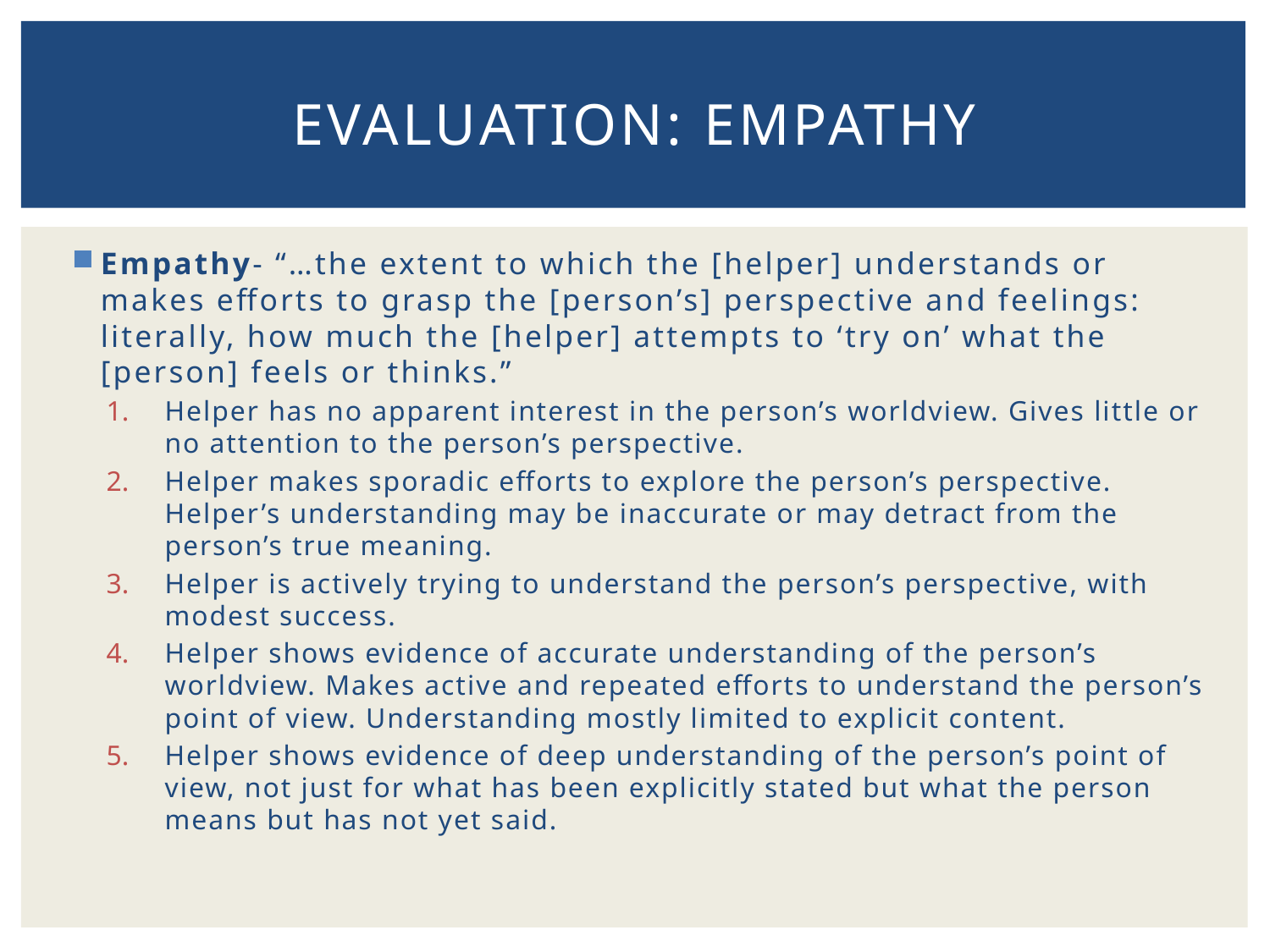

# Evaluation: empathy
Empathy- “…the extent to which the [helper] understands or makes efforts to grasp the [person’s] perspective and feelings: literally, how much the [helper] attempts to ‘try on’ what the [person] feels or thinks.”
Helper has no apparent interest in the person’s worldview. Gives little or no attention to the person’s perspective.
Helper makes sporadic efforts to explore the person’s perspective. Helper’s understanding may be inaccurate or may detract from the person’s true meaning.
Helper is actively trying to understand the person’s perspective, with modest success.
Helper shows evidence of accurate understanding of the person’s worldview. Makes active and repeated efforts to understand the person’s point of view. Understanding mostly limited to explicit content.
Helper shows evidence of deep understanding of the person’s point of view, not just for what has been explicitly stated but what the person means but has not yet said.

## Slide 44
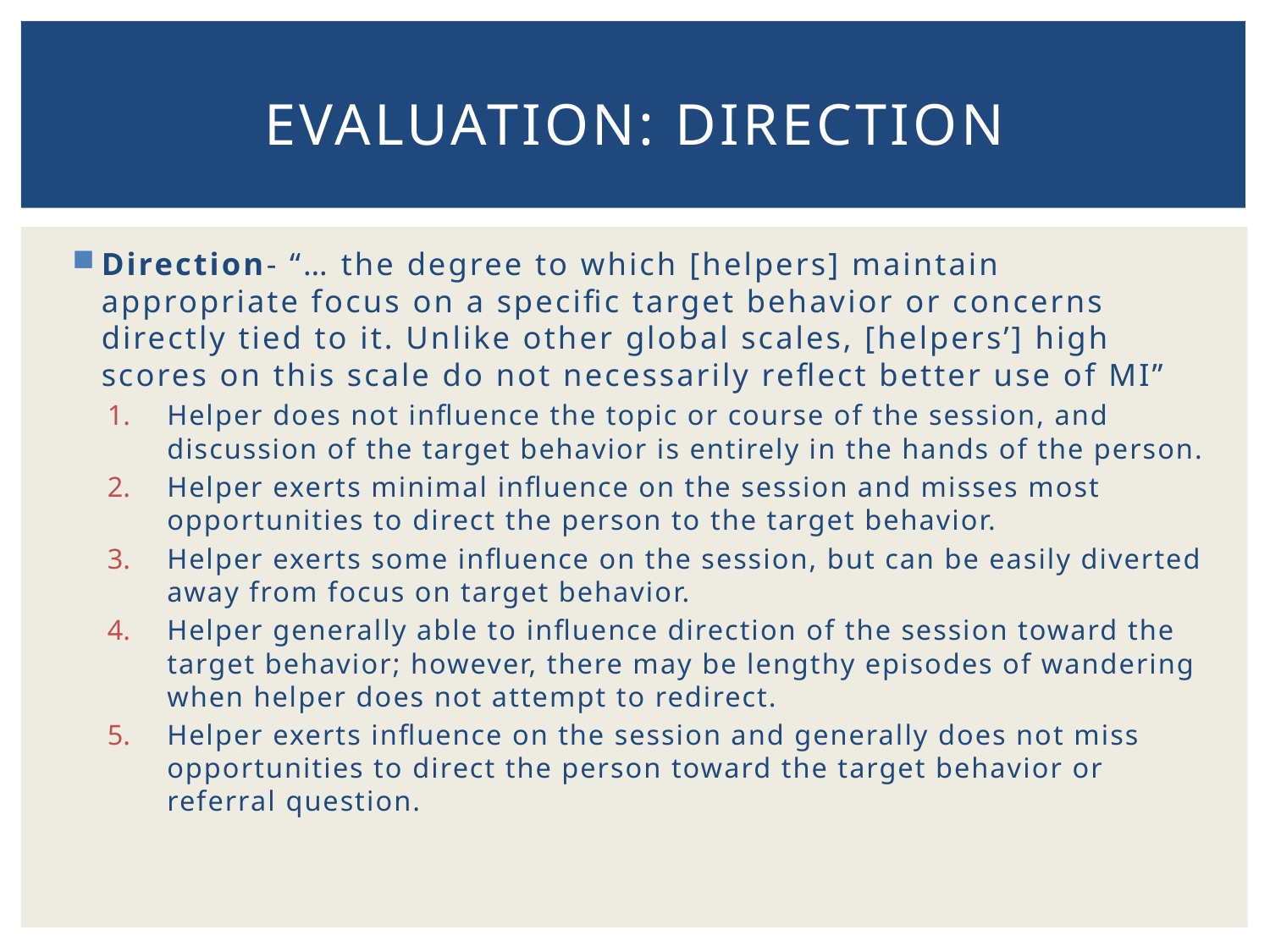

# Evaluation: direction
Direction- “… the degree to which [helpers] maintain appropriate focus on a specific target behavior or concerns directly tied to it. Unlike other global scales, [helpers’] high scores on this scale do not necessarily reflect better use of MI”
Helper does not influence the topic or course of the session, and discussion of the target behavior is entirely in the hands of the person.
Helper exerts minimal influence on the session and misses most opportunities to direct the person to the target behavior.
Helper exerts some influence on the session, but can be easily diverted away from focus on target behavior.
Helper generally able to influence direction of the session toward the target behavior; however, there may be lengthy episodes of wandering when helper does not attempt to redirect.
Helper exerts influence on the session and generally does not miss opportunities to direct the person toward the target behavior or referral question.

## Slide 45
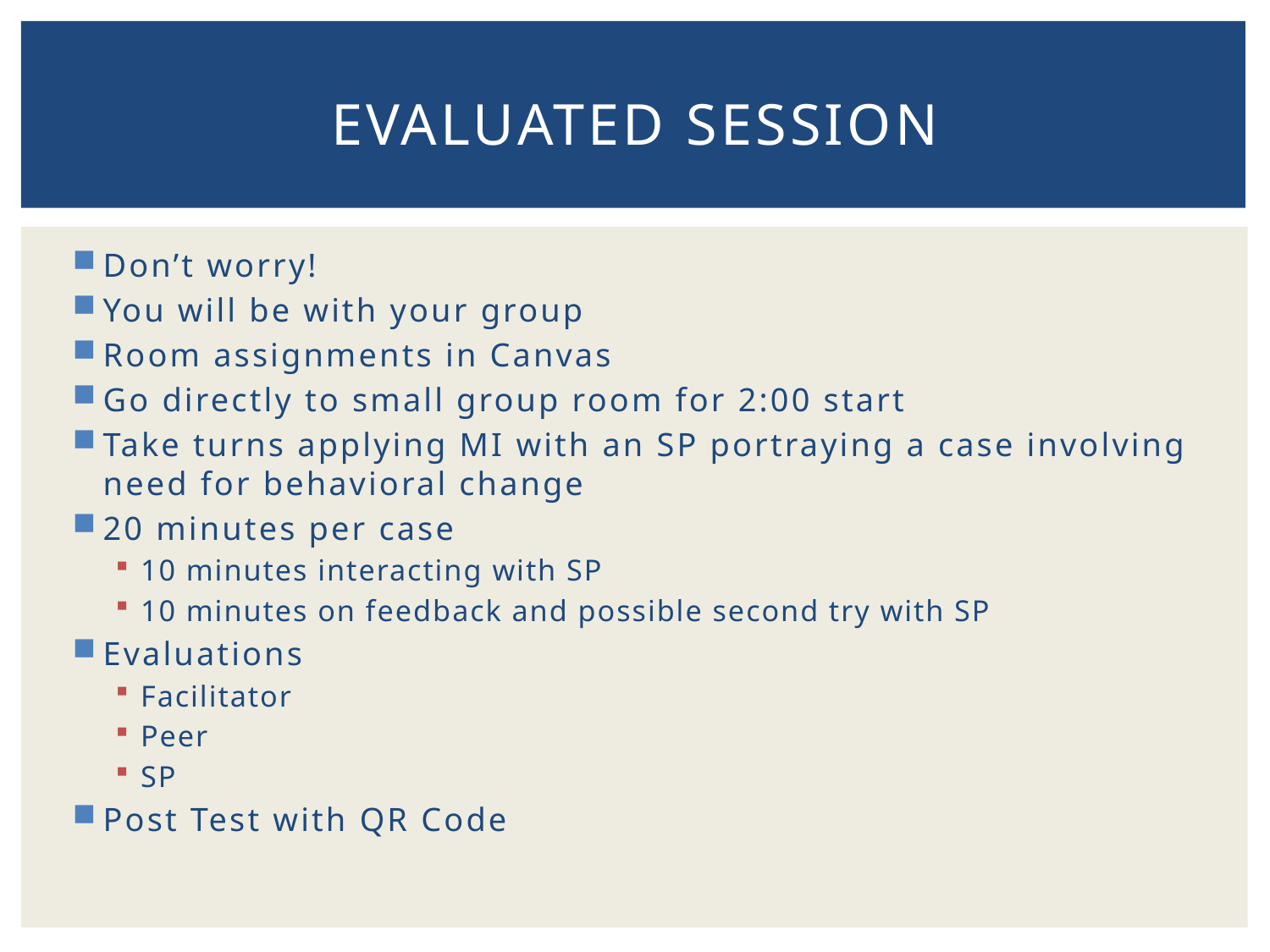

# Evaluated Session
Don’t worry!
You will be with your group
Room assignments in Canvas
Go directly to small group room for 2:00 start
Take turns applying MI with an SP portraying a case involving need for behavioral change
20 minutes per case
10 minutes interacting with SP
10 minutes on feedback and possible second try with SP
Evaluations
Facilitator
Peer
SP
Post Test with QR Code

## Slide 46
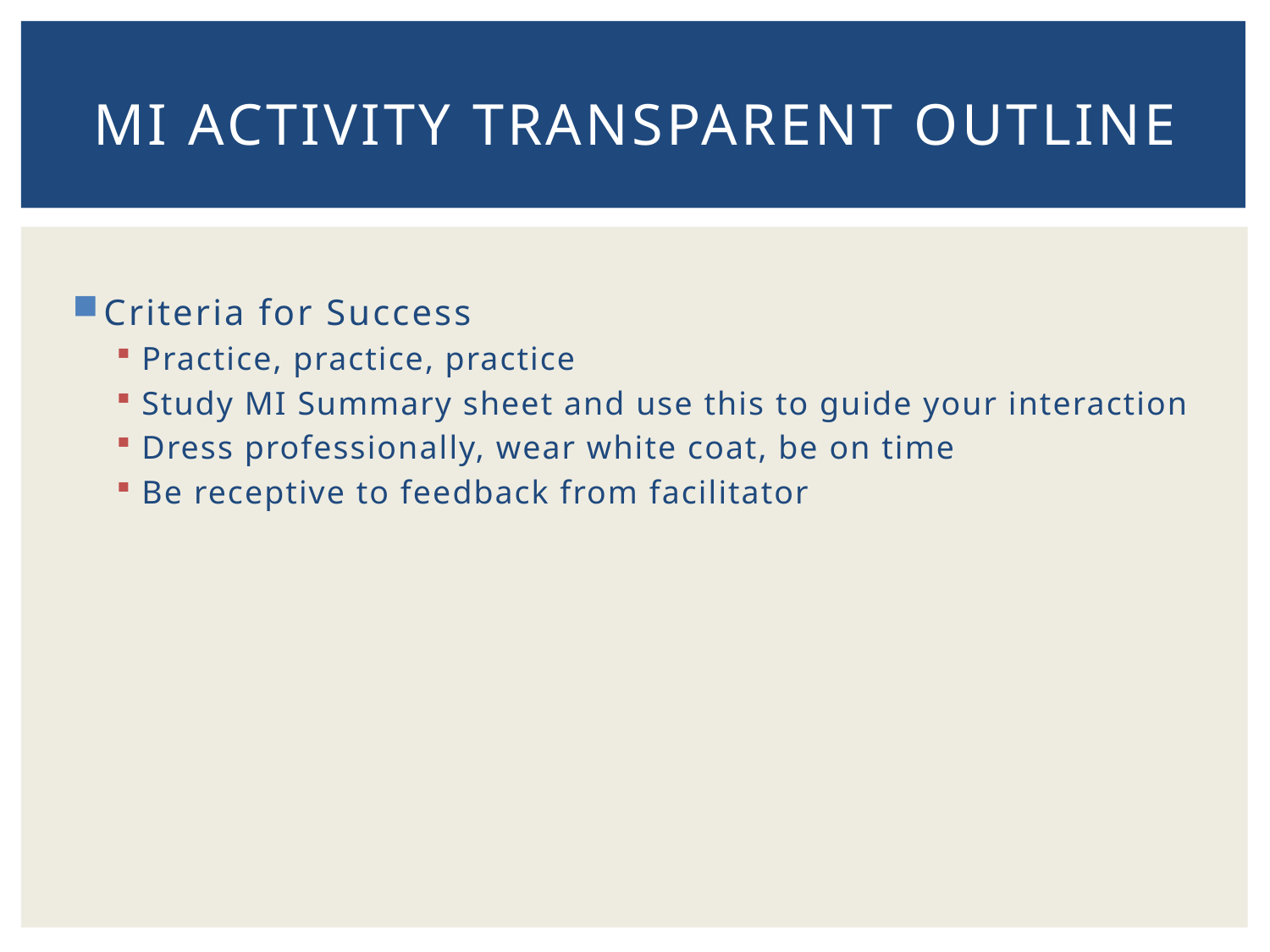

# MI Activity Transparent Outline
Criteria for Success
Practice, practice, practice
Study MI Summary sheet and use this to guide your interaction
Dress professionally, wear white coat, be on time
Be receptive to feedback from facilitator

## Slide 47
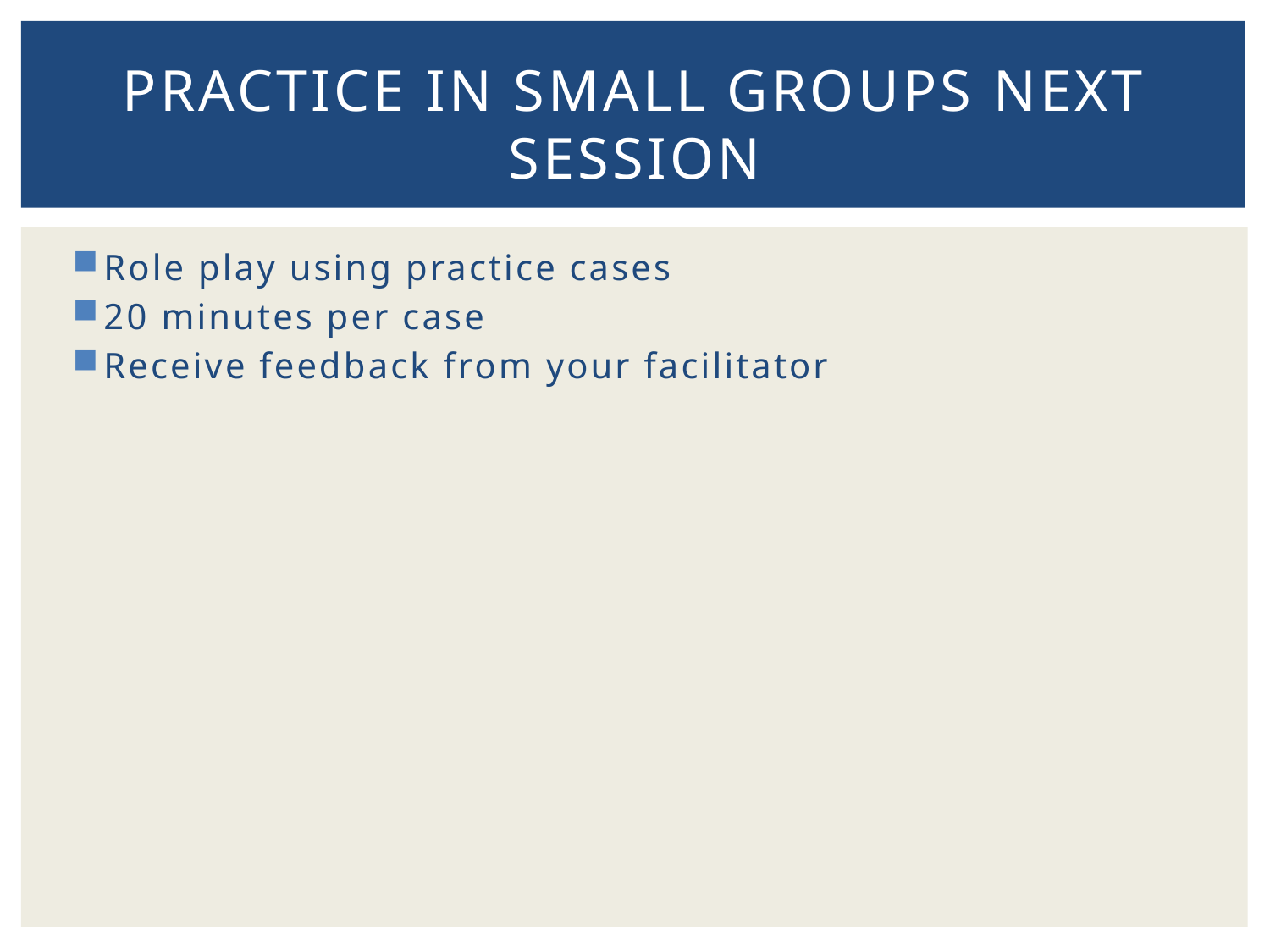

# Practice in small groups next session
Role play using practice cases
20 minutes per case
Receive feedback from your facilitator

## Slide 48
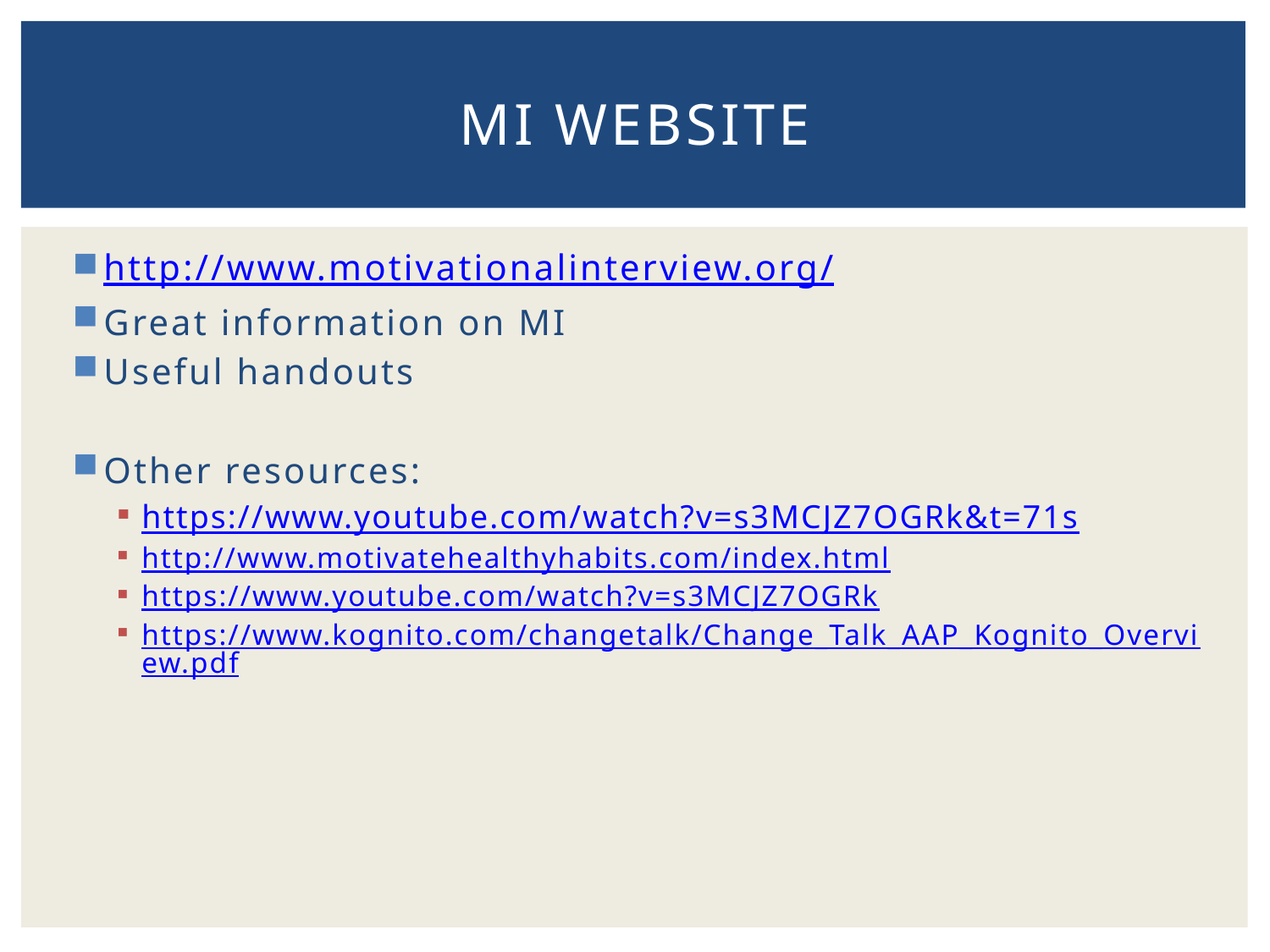

# MI Website
http://www.motivationalinterview.org/
Great information on MI
Useful handouts
Other resources:
https://www.youtube.com/watch?v=s3MCJZ7OGRk&t=71s
http://www.motivatehealthyhabits.com/index.html
https://www.youtube.com/watch?v=s3MCJZ7OGRk
https://www.kognito.com/changetalk/Change_Talk_AAP_Kognito_Overview.pdf

## Slide 49
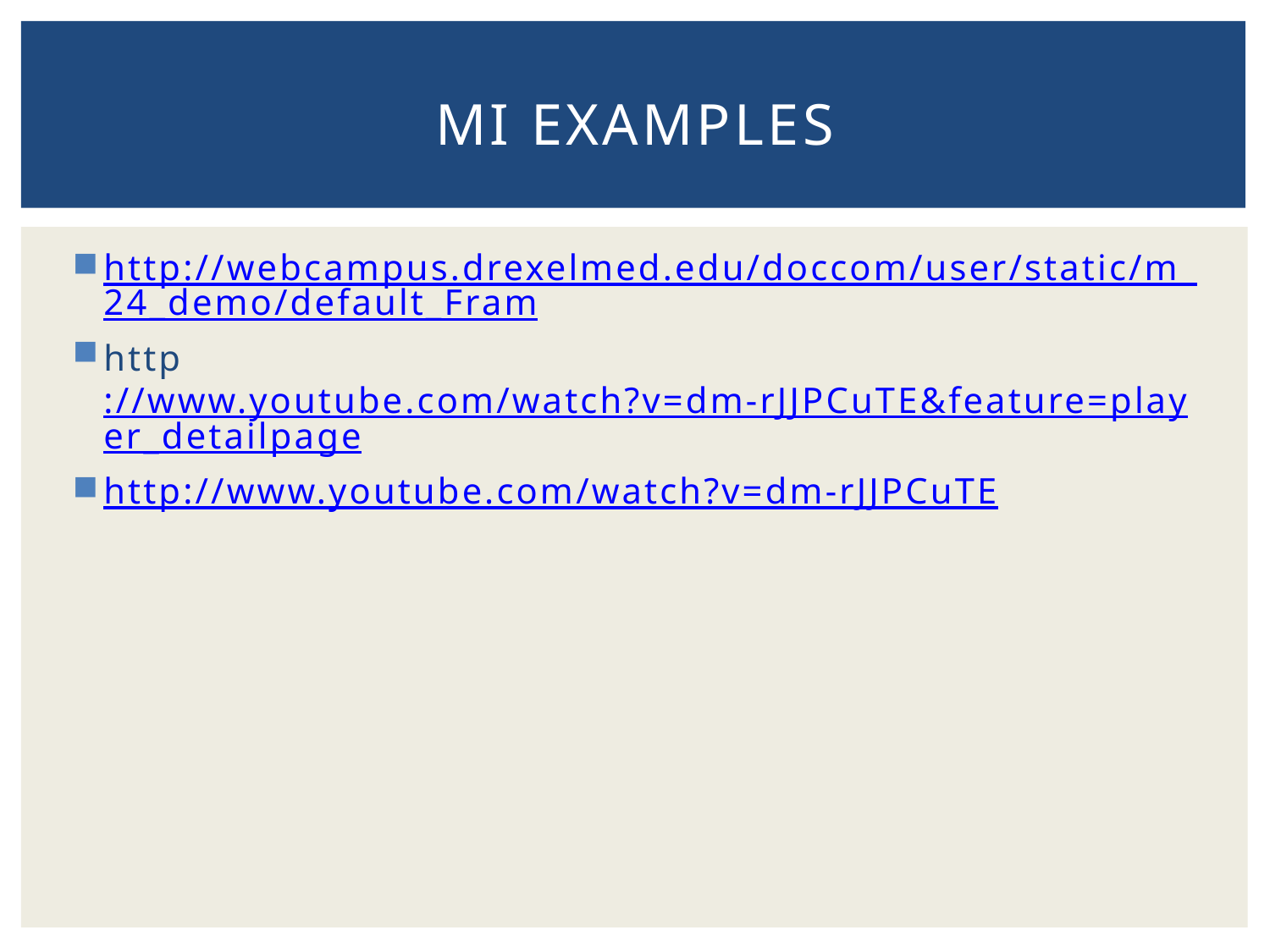

# MI examples
http://webcampus.drexelmed.edu/doccom/user/static/m_24_demo/default_Fram
http://www.youtube.com/watch?v=dm-rJJPCuTE&feature=player_detailpage
http://www.youtube.com/watch?v=dm-rJJPCuTE

## Slide 50
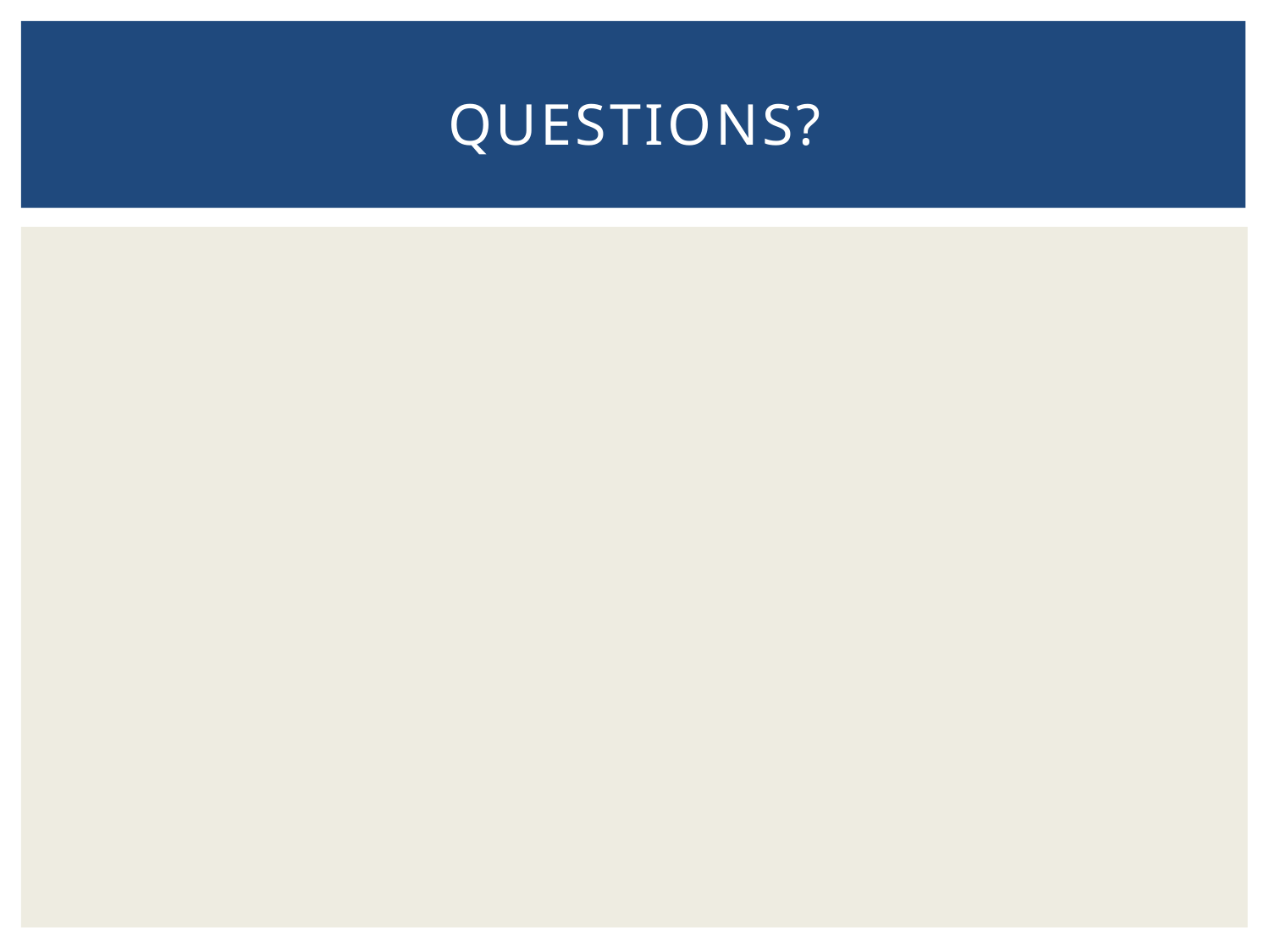

# Questions?
