# Supplementary material for: Motivational Interviewing Training: A Case-Based Curriculum for Preclinical Medical Students
Source: MedEdPORTAL. 2021 Feb 12;17:11104. doi: 10.15766/mep_2374-8265.11104 (PMC7880250; doi:10.15766/mep_2374-8265.11104)
Supplement: Supplementary file 1 — Presurvey.docxMI Presentation.pptxMI Demonstration Script.docxTransparent Outline for MI Activity.docxMICA Evaluation Tool.docPractice Cases.docxMI Summary Sheet.docxEvaluated Cases.docxOARS Tracking Sheet.docChange Talk Tracking Sheet.docMI Evaluated Session Sample Schedule.xlsxActing Patient Experience Scale.docxPostsurvey.docxFacilitator Guide.docx [file mep_2374-8265.11104-s001.zip › C. MI Demonstration Script.docx]

**MI Demonstration Script**

**Case**

Jenny O is a 55-year-old overweight patient with hypertension, hyperlipidemia, and knee arthritis, seeing you for a return visit. At the last visit (6 months ago) you prescribed a beta blocker and a statin, and suggested she start exercising. Today, she tells you that she has not been taking her meds because she “feels fine.” She says she can’t exercise because she is too busy caring for her grandkids and because of her knee and back pain. She wants you to “just fix it” so she can be healthy again.

**Script**

Doctor: What do you see as your major health issue right now?

Jenny: Well, Doc, it’s like you keep telling me, I’ve got to get my blood pressure and cholesterol under control, and I need to start exercising so I can lose some weight. It’s just really hard to do all of that.

Doctor: What are your goals with your health right now?

Jenny: I really don’t like taking those medication for my blood pressure and cholesterol. I want to get off those medications. And I’m sick of having knee pain. I can’t even play tennis anymore because it hurts so bad.

Doctor: What is your understanding about the way in which your diet and weight affect your blood pressure, cholesterol, and pain?

Jenny: I’m not totally sure, but I guess they’re probably related.

Doctor: Is it alright if I tell you about the connections between your diet and weight and your health?

Jenny: Sure.

Doctor: Great, thank you. Unhealthy foods, such as processed foods, can increase your blood pressure as well as your cholesterol, and they can cause weight gain. And, the more weight your knees have to carry around, the more pain that can cause in your knees. Now that you are aware of those connections, where does that leave you?

Jenny: So, it sounds like I need to have a healthier diet, so that I can lower my blood pressure and cholesterol. And, if I lose weight, it might make me have less pain, so maybe then I could return to tennis.

Doctor: What are the good things about your diet right now?

Jenny: Well, the processed food is easy to make, and eating really helps me to relax.

Doctor: What are the less good things about your diet right now?

Jenny: Well, I guess it makes me gain weight, which is bad for my knee pain, and it is not so good for my blood pressure or cholesterol.

Doctor: Is there anything that worries you about your diet?

Jenny: Yeah, my dad died in his 50’s of heart disease, and I don’t want to end up like him. I really want to be there for my kids and grandkids.

Doctor: What could happen if you continued to eat the way you are eating now?

Jenny: I could keep on gaining weight, which could limit my physical activities. And, I would need to stay on those medications I hate taking. And I definitely don’t want to die like my dad.

Doctor: Earlier you told me that your health goals are to not have to take those medications and to return to playing tennis. How does continuing to eat your current diet fit in with the goals you have for your health?

Jenny: Well, I guess it doesn’t.

Doctor: What would be the good things about changing your diet so that you are eating more healthy?

Jenny: I could quit those awful medications, and I could get back to tennis. And, I would have a better chance of being around for my kids and being able to watch my grandkids grow up.

Doctor: On a scale from 1 to 10, with 1 being not important at all and 10 being of the utmost importance, how important is it for you right now to modify your diet?

Jenny: I’d say a 7.

Doctor: Why a 7 and not a 4?

Jenny: Because I really love my family, I really want to get back to playing tennis, and I hate taking those medications.

Doctor: On a scale from 1 to 10, with 1 being not confident at all and 10 being the most confident, how confident are you that you can change your diet?

Jenny: Probably a 6.

Doctor: Why did you choose 6 and not 3?

Jenny: I have been able to eat healthier in the past, and I even lost 20 pounds once.

Doctor: What strategies have worked for you in the past to lose weight?

Jenny: That time I lost 20 pounds was because I went on a low-carb diet with my husband.

Doctor: What change strategies do you think will work for you now?

Jenny: I guess I could do that low-carb diet again, and maybe I could get my husband on board. He could stand to lose a few pounds too!

Doctor: What would you be willing to try?

Jenny: I’m willing to go on that diet.

Doctor: What do you intend to do when you leave here today?

Jenny: When I get home, I’m going to start that low-carb diet, and I’ll talk my husband into joining me.

Doctor: That is fantastic! In my experience, patients do better when they set goals that are specific, measurable, achievable, relevant, and time bound. How can you do this?

Jenny: I can use the same low-carb diet that worked last time and I can promise to do it for at least two months. Can I come back then to get weighed?

Doctor: That sounds great. And we can do some lab work then too, so you can see how the diet is affecting your blood pressure and cholesterol. Do you foresee any potential barriers getting in your way of this plan?

Jenny: The only thing is if I get really anxious. That is really why I crave the carbs. Maybe I need to start meditating again.

Doctor: Jenny, that is a great plan. I’m right behind you and happy to help however I can. I look forward to seeing you back in 2 months.

Jenny: Thank you, Doctor. See you soon.
