# Supplementary material for: Motivational Interviewing Training: A Case-Based Curriculum for Preclinical Medical Students
Source: MedEdPORTAL. 2021 Feb 12;17:11104. doi: 10.15766/mep_2374-8265.11104 (PMC7880250; doi:10.15766/mep_2374-8265.11104)

**Transparent Outline for Motivational Interviewing Activity**

Note: This should be tailored to your institution

**Assignment Name:** Motivational Interviewing Activity

**Purpose:** A large portion of patient presenting complaints in primary care involve medical problems that are caused or exacerbated by lifestyle issues, such as diet, exercise, smoking, and substance use, as well as patient adherence. The purpose of this activity is to allow students to demonstrate the motivational interviewing skills and techniques they have learned during the Toolkit lecture. This activity also introduces students to a common form of evaluation they will experience in medical school in which they interact with standardized patients. This will ultimately prepare them to complete the USMLE Step II clinical skills exam that occurs after the 3^rd^ year of medical school. Additionally, the practice of interviewing a standardized patient in a group setting and getting feedback from a facilitator will train students for rounding with preceptors/attendings in the future.

**Skills:** The purpose of this assignment is to help you practice the following skills that are essential to your success as a clinician (adapted from UNR Med Medical Education Program Objectives):

- **Interpersonal and Communication Skills**: *Demonstrate interpersonal and communication skills that result in the effective exchange of information and collaboration with patients, their families, and health professionals*

1. Communicate effectively with patients, families, and the public, as appropriate, across a broad range of socioeconomic and cultural backgrounds

6. Demonstrate insight and understanding about emotions and human responses to emotions that allow one to develop and manage interpersonal interactions

7. Demonstrate insight and understanding about emotions and human responses to emotions that allow one to develop and manage interpersonal interactions

- **Professionalism**: *Demonstrate a commitment to carrying out professional responsibilities and an adherence to ethical principles*

1. Demonstrate compassion, integrity, and respect for others
2. Demonstrate responsiveness to patient needs that supersedes self-interest
3. Demonstrate respect for patient privacy and autonomy
4. Demonstrate accountability to patients, society, and the profession
5. Demonstrate sensitivity and responsiveness to a diverse patient population, including but not limited to diversity in gender, age, culture, race, religion, disabilities, and sexual orientation

- **Personal and Professional Development**: *Demonstrate the qualities required to sustain lifelong personal and professional growth*

4. Practice flexibility and maturity in adjusting to change with the capacity to alter one's behavior

- **Patient Care**: *Provide patient-centered care that is compassionate, appropriate, and effective for the treatment of health problems and the promotion of health*

5. Make informed decisions about diagnostic and therapeutic interventions based on patient information and preferences, up-to-date scientific evidence, and clinical judgment

6. Develop and carry out patient management plans

7. Counsel and educate patients and their families to empower them to participate in their care and enable shared decision-making

9. Provide health care services to patients, families, and communities aimed at preventing health problems or maintaining health

**Knowledge for Practice**: *Demonstrate knowledge of established and evolving biomedical, clinical, epidemiological and social-behavioral sciences, as well as the application of this knowledge to patient care*

1. Demonstrate an investigatory and analytic approach to clinical situations
2. Apply established and emerging bio-physical scientific principles fundamental to health care for patients and populations

5. Apply principles of social-behavioral sciences to provision of patient care, including assessment of the impact of psychosocial and cultural influences on health, disease, care seeking, care compliance, and barriers to and attitudes toward care

**Task:** Following a practice session, the MI activity involves a 5-10 minute interaction with a Standardized Patient who presents with a medical problem involving the need for behavioral change. Students should demonstrate use of MI skills and techniques to move patients through the Stages of Change model, such as interviewing the patient to learn what the behavioral problem is and how it relates to their medical/health goals and values, developing discrepancy between the patient’s health behavior and their desired goals, avoiding the righting reflex, expressing empathy, rolling with resistance, and supporting patient self-efficacy while honoring patient autonomy. Students should demonstrate use of open-ended questions to evoke patient’s reasons for change, methods to modify their problematic lifestyle behaviors, and confidence regarding change while demonstrating use of affirmations, reflections, and summaries. If needed, change rulers can be used to elicit talk about patient’s reasons for change and confidence in making the change. If they provide information to patients, they should use the ask-provide-ask technique. If patient indicates a readiness for change, students can help patients to develop a plan for change by sharpening the focus of the patient-centered plan and setting smart goals. After students are finished with the patient interaction, they will receive feedback from their facilitator and peers.

**Criteria for Success:** Students who traditionally do well on this encounter are those who have used time outside of class to practice the skills and techniques involved in MI. We suggest reviewing all class material and then working with your classmates to practice. Cases for the evaluated encounter will be just like those used during the practice session. Be sure to demonstrate the components of the task, as described above and summarized in your MI Summary Sheet. Successful students should also demonstrate receptivity to feedback received from their facilitator and peers. Students will also need to ensure you dress professionally, wear their white coat, and be on time to the assigned time slot.

**Advice from a Previous Student**: *“Motivational Interviewing is a skill that is taught in the first two years of medical school and will be continued to be used throughout your clinical years and on into practice. The training can be nerve wracking and uncomfortable, despite all efforts taken to make it low stakes and straight forward. However stressful they find it, I encourage students to take full advantage of this opportunity to learn motivational interviewing. It may seem silly and “not worth the time spent” but having gone through clerkships now, I can say that this skill set has come up in every rotation I’ve been on and several attendings have commented on how much they appreciate that our students are already familiar with this method. I have used motivational interviewing during end of life care discussions, smoking and alcohol cessation talks, encouraging vaccinations, and consenting for surgery and have had it mentioned in my clerkship evaluations. I remember going through the MI trainings and feeling hugely uncomfortable with the idea of role playing through the script but that was still very valuable looking back. I learned quickly in med school how much of medicine is learning the “script.” Just like students have had to learn the “script” for invasive physical exam maneuvers, it’s important to practice what to say in motivational interviewing. Take the opportunity provided here to learn how to do this and it will serve you well going forward.”*

The author developed an earlier version of this template at the University of Illinois, Urbana-Champaign.

1 Winkelmes, Mary-Ann. “Transparency in Teaching: Faculty Share Data and Improve Students’ Learning.” *Liberal Education* 99,2 (Spring 2013); Winkelmes et al, “A Teaching Intervention that Increases Underserved College Students’ Success.” *Peer Review* 18,1/2 (Winter/Spring 2016).


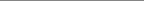

Supplement: Supplementary file 1 — Presurvey.docxMI Presentation.pptxMI Demonstration Script.docxTransparent Outline for MI Activity.docxMICA Evaluation Tool.docPractice Cases.docxMI Summary Sheet.docxEvaluated Cases.docxOARS Tracking Sheet.docChange Talk Tracking Sheet.docMI Evaluated Session Sample Schedule.xlsxActing Patient Experience Scale.docxPostsurvey.docxFacilitator Guide.docx [file mep_2374-8265.11104-s001.zip › D. Transparent Outline for MI Activity.docx]
