# Supplementary material for: Motivational Interviewing Training: A Case-Based Curriculum for Preclinical Medical Students
Source: MedEdPORTAL. 2021 Feb 12;17:11104. doi: 10.15766/mep_2374-8265.11104 (PMC7880250; doi:10.15766/mep_2374-8265.11104)
Supplement: Supplementary file 1 — Presurvey.docxMI Presentation.pptxMI Demonstration Script.docxTransparent Outline for MI Activity.docxMICA Evaluation Tool.docPractice Cases.docxMI Summary Sheet.docxEvaluated Cases.docxOARS Tracking Sheet.docChange Talk Tracking Sheet.docMI Evaluated Session Sample Schedule.xlsxActing Patient Experience Scale.docxPostsurvey.docxFacilitator Guide.docx [file mep_2374-8265.11104-s001.zip › F. Practice Cases.docx]

**Missing Clinic Appointments**

Presentation to Student:

Kristin/Chris Wolff is a 50 year old postal carrier with past medical history of poorly controlled diabetes and chronic diabetic foot ulcer who you are seeing in primary care clinic for a follow up visit. (S)he has been referred to the Limb Preservation Clinic, however has no-showed two appointments with them. Their office has called to tell you that if (s)he no-shows another appointment, (s)he will be let go as a patient. You are concerned that without proper wound care, (s)he might ultimately require an amputation.

Information for the Standardized Patient:

50 years old

History: due to diabetic nerve disease, you have no pain or symptoms otherwise regarding your wound. There is no sensation in your foot, so there is no pain. You have not noticed any drainage or changes to the wound, but admit that you don’t really check it that often – only about once a week when you remember. You have had no other symptoms associated with this wound and are feeling fine otherwise. You check your blood sugar only about twice a week, and it’s usually around 250.

You have been no-showing your Limb Preservation Clinic appointments since you feel well and your wound doesn’t bother you.

Your brother, who also has diabetes, lost three fingers due to diabetic ulcers, and now has a hard time with his daily activities. You see how he suffered from his wound and struggles now with his amputations, and don’t want to get amputation yourself. However, getting to wound clinic doesn’t feel like a priority because you feel well now.

ROS: otherwise negative
PMH: Poorly controlled diabetes, chronic diabetic right foot ulcer

Family History: both parents with diabetes, brother with diabetes who also had three fingers on right hand amputated due to wound

Social History: no tobacco, alcohol or illegal drug use. You deliver mail on foot and you enjoy your job. You need to be able to walk in order to do your job.

Meds: Metformin twice daily, insulin 20 units daily

MI Information

- What are the patient’s life goals and values, especially with respect to the target behavior and overall health? Family? Work? Finances? Patient wants to be able to continue to deliver mail on foot, because they love their job. Patient also saw the suffering of his/her brother and doesn’t want to end up like him.
- How does continuation of the target behavior fit in or conflict with these goals? Getting an amputation would disrupt the patient’s ability to work and would cause the suffering patient has seen his/her brother go through.
- What are the good things about the target behavior? How does it benefit you? Not going to the Limb Preservation Clinic appointments allows the patient to not have think about the threat of amputation. Patient doesn’t like to be reminded of the seriousness of his/her diabetes and the potential consequences of the disease, like amputation.
- What are the less good things about the target behavior? What are you afraid might happen if you keep engaging in the target behavior? Patient is afraid of losing his/her limb, which would mean losing the job he/she loves.
- What would be the advantages of making the behavior change? What are the best reasons to change? Patient really enjoys being able to work as a postal carrier and needs to be able to walk in order to continue this job.
- Explore ways to make the change (pros and cons of various methods, what has worked in the past). Patient could put a picture of a walking postal carrier in his/her wallet/home/car to as a reminder of how much he/she loves the job and how important it is to keep clinic appointments so he/she can continue to work. Or, patient could enlist the help of her/his brother for encouragement to go to clinic appointments. Or, patient could ask for clinic reminders, put it on their calendar, etc.
- Importance and confidence of changing the target behavior:
  - How important is it for you to change?
    - Why is that number that high and not lower? 8, not lower because she/he can’t imagine not working as a postal carrier and truly doesn’t want to suffer the way her/his brother did.
    - How could you move that number higher? Patient could spend more time with her/his brother or look at a picture of a walking postal carrier as a reminder of how important that job is.
  - How confident are you that you can change?
    - Why is that number that high and not lower? 4, not lower because when patient first got diagnosed with diabetes he/she was really good at going to appointments, managing diet, and testing sugars.
    - What would make you more confident in your ability to change? Having brother’s help, getting reminders, remembering that patient needs to have both limbs to do the job they love.
- Ask for a decision: Patient will ask his/her brother for support, will ask for time off work to attend the appointment, will ask for clinic reminders.
- Set SMART (specific, meaningful, assessable, realistic, timed) goals. What are the first steps? Make another appointment, put the appointment in patient’s calendar, call brother to ask for support.
- What do you intend to do? Call the clinic to make an appointment and call the brother to ask for help.

**Not wearing seat belt**

Presentation to Student:

Rich/Rita Allen is a 45 year old patient who you have been seeing in clinic for years. (S)he is otherwise healthy, but on a new health questionnaire you have started using in your clinic, (s)he indicated that (s)he does not wear a seatbelt. You are concerned about this health risk. The patient is in a hurry to finish the appointment because it’s his/her nephew’s 8^th^ birthday (who patient is like a parent to), and he/she plans on taking him to his favorite restaurant in town. The nephew is excited about his birthday, since he is now old enough to not need a car seat.

Information for the Standardized Patient:

45 years old

History: Otherwise healthy patient with no medical problems. You indicated on the clinic health questionnaire completed just prior to your annual check up with your primary care provider you know very well that you do not regularly use a seat belt. There is no particular reason for it, you never have worn a seat belt and you’ve been “just fine”. You drive mainly on local/residential rounds around town, and aren’t usually on the freeway, so you don’t see a need for one. You have had only a minor fender-bender a few years ago during which you weren’t injured, so that reinforced your belief that seat belts aren’t necessary for just driving around town.

ROS: negative

PMH: none

Fam Hx: none

Meds: none

Social History: no tobacco, alcohol or illegal drug use

No medications or allergies

MI Information

- What are the patient’s life goals and values, especially with respect to the target behavior and overall health? Family? Work? Finances? Patient wants to continue to stay safe and wants to get his/her nephew to dinner. Patient wants to be a good role-model to his/her nephew since he/she is like a parent (mother/father figure) to them.
- How does continuation of the target behavior fit in or conflict with these goals? Not wearing a seat belt puts patient at risk for continuing to stay healthy. Not wearing a seat belt makes patient a poor role model for the nephew.
- What are the good things about the target behavior? How does it benefit you? It is nice not to have to worry about safety and seat belts. Patient feels invincible and wants to keep believing this.
- What are the less good things about the target behavior? What are you afraid might happen if you keep engaging in the target behavior? Patient is afraid of being a poor role model for the nephew. Patient adores the nephew and wants him to be safe by wearing a seat belt, but feels it would be hypocritical if he/she didn’t wear a seat belt while asking the nephew to wear one.
- What would be the advantages of making the behavior change? What are the best reasons to change? Keeping the nephew safe and being a good role model for the nephew.
- Explore ways to make the change (pros and cons of various methods, what has worked in the past). Having a reminder in the car, like a picture of the nephew.
- Importance and confidence of changing the target behavior:
  - How important is it for you to change? 10
  - Why is that number that high and not lower? Not lower because patient loves his/her nephew
    - How could you move that number higher? Already as high as it can go.
  - How confident are you that you can change? 5
    - Why is that number that high and not lower? Not lower because you used to wear your seat belt when you first started to drive.
    - What would make you more confident in your ability to change? Reminders
- Ask for a decision. Patient will put a picture of his/her nephew in the car as a reminder to use seat belts.
- Set SMART (specific, meaningful, assessable, realistic, timed) goals. What are the first steps? Take a picture of the nephew tonight at dinner and print it out to put in the car.
- What do you intend to do? Take a picture and print it out, then tape it to the steering wheel.

**Not getting infected tooth pulled**

Presentation to Student:

David/Diana Thompson is a 40 year old patient you are seeing back in the emergency department for the second visit in a week for tooth pain. On last visit, you diagnosed him/her with an infected tooth and referred him/her to the dentist for tooth extraction. The patient is back with worse pain and a fever, after failing to schedule a dental appointment. You are concerned that if the tooth doesn’t get extracted the patient might get a serious infection and need surgery.

Information for the Standardized Patient:

40 years old

History: You have returned for a second visit to the ER with complaints of progressive achy tooth pain, as high as 7/10, currently 3/10, and have now had fever to 101 at home. Your tooth hurts on your right lower jaw, and you haven’t been able to eat much because of the pain. You acknowledge that the doctor referred you to a dentist, but you were too scared of the dentist to make the appointment. You’ve had bad experiences with dentists in the past and figured the pain would go away, which it hasn’t. It’s been about 15 years since you last saw a dentist, and that was for a cavity. You are worried that if you tooth gets worse, you’ll get a serious infection and need surgery, which you heard happening to one of your friends’ coworkers.

ROS: fever to 101 last night, decreased oral intake due to pain

PMH: no other medical problems

Family history: none

Social history: no tobacco, alcohol or illegal drug use

No medications or allergies

MI Information

- What are the patient’s life goals and values, especially with respect to the target behavior and overall health? Family? Work? Finances? Patient’s goal is to avoid surgery, which would be even worse than seeing a dentist.
- How does continuation of the target behavior fit in or conflict with these goals? Not seeing a dentist to get the infected tooth pulled puts you at risk for a more serious infection and need for surgery.
- What are the good things about the target behavior? How does it benefit you? Avoiding the dentist allows negative reinforcement of patient’s fear of dentists.
- What are the less good things about the target behavior? What are you afraid might happen if you keep engaging in the target behavior? Patient is afraid of needing a surgery.
- What would be the advantages of making the behavior change? What are the best reasons to change? Being healthy and being able to avoid surgery.
- Explore ways to make the change (pros and cons of various methods, what has worked in the past). Asking for laughing gas, taking a trusted friend with you to the dentist for emotional support, etc.
- Importance and confidence of changing the target behavior:
  - How important is it for you to change? 5
    - Why is that number that high and not lower? Not lower because patient really doesn’t want surgery.
    - How could you move that number higher? Thinking about how getting a surgery would be worse than going to a dentist.
  - How confident are you that you can change? 6
    - Why is that number that high and not lower? You are also afraid of the doctor but you are able to push yourself to go by taking a trusted friend with you to the appointment.
    - What would make you more confident in your ability to change? Getting a friend to accompany you to the appointment.
- Ask for a decision. Patient will call a friend and ask them to go to the dentist appointment with him/her.
- Set SMART (specific, meaningful, assessable, realistic, timed) goals. What are the first steps? Call the friend, make another dentist appointment.
- What do you intend to do? Make another dentist appointment and call a friend for help.

**HIV medication compliance**

Information for the student:

Wade/Wendy Thompson is a 55 year old male/female returning to clinic for routine lab follow up visit. He/she has a history of HIV, diagnosed about 10 years ago. Overall, he/she has had variable compliance with medications. When he/she doesn’t take his/her medications for HIV, he/she often requires hospitalization for infections, such as pneumonia. Patient is afraid of losing his/her job due to these hospitalizations. Today, his/her CD4 count is low and his/her viral load is high (these labs have worsened since his/her last visit) and he/she admits to not taking his/her medications recently.

Information for the SP:

You are a 55 year old patient with HIV. You were diagnosed about 10 years ago and you have had times when you have taken your medications as directed, and times when you stop taking them. You love your work at a local bookstore and when you are feeling well, it is tough to remember the daily medication. When asked about your hospitalizations, you have had pneumonia multiple times, always when you stopped your HIV medications, and the recovery process is difficult to regain your strength. You have had the discussion with your provider that when your CD4 count is low and viral load is high, you are at risk for more infections. You are agreeable to finding ways to improve remembering your daily medicine, even when you are feeling well. Your boss is frustrated by your frequent use of sick leave and you are now out of sick leave. Your boss has threatened to fire you if you need any more time off work. If you lose your job you will also lose your insurance, and you can’t afford to do that.

PMH: You do not have any additional medical history

Medication: Atripla 1 tablet daily

No allergies, surgeries or family history

Social history: nonsmoker, no alcohol intake, no drug use, work in a bookstore, monogamous relationship with partner for the past 15 years

MI Information

- What are the patient’s life goals and values, especially with respect to the target behavior and overall health? Family? Work? Finances? You love your job at the bookstore and need to continue to work so you can keep your insurance.
- How does continuation of the target behavior fit in or conflict with these goals? If you don’t take your HIV meds you might need to be hospitalized again, which would threaten your job and insurance.
- What are the good things about the target behavior? How does it benefit you? Taking HIV meds is a reminder that you are sick, and not taking them allows you to avoid remembering you are sick even when you aren’t feeling sick.
- What are the less good things about the target behavior? What are you afraid might happen if you keep engaging in the target behavior? You are afraid of another hospitalization, which would threaten your job and insurance.
- What would be the advantages of making the behavior change? What are the best reasons to change? Staying out of the hospital, being able to continue working, keeping your much needed insurance.
- Explore ways to make the change (pros and cons of various methods, what has worked in the past). You could put reminders in your phone, or download an app that sends you reminders, use pill boxes, pair pill-taking with other well-established behaviors like brushing teeth, etc., put reminders all over the place, enlist your partner’s support to help you remember to take your pills, etc.
- Importance and confidence of changing the target behavior:
  - How important is it for you to change? 7
    - Why is that number that high and not lower? You really love your job and you really hate the hospital.
    - How could you move that number higher? Take that horrible picture of yourself from your last hospitalization and put it next to your medications.
  - How confident are you that you can change? 5
    - Why is that number that high and not lower? You remember to take your meds when you are ill, so you know you can do it.
    - What would make you more confident in your ability to change? Employing some of the strategies for remembering to take your meds, like apps or text reminders on your phone.
- Ask for a decision. You will look for a reminder app.
- Set SMART (specific, meaningful, assessable, realistic, timed) goals. What are the first steps? You will find and download an app in the next week, and you will ask your partner for support.
- What do you intend to do? Look for an app and talk with your partner.

**Smoking while pregnant**

Presentation to Student:

Julie Sussman is a 35 year old patient you are seeing for a routine prenatal visit. She is currently 12 weeks pregnant and is feeling well and having no pregnancy-related concerns. She is, however, still smoking. You know her from her last pregnancy 5 years prior, during which she was able to successfully quit smoking. She is considering quitting, but uses smoking as her way to “manage” the stress of trying to balance work and increasingly frequent ER visits for her older son due to asthma.

Information for the Standardized Patient:

History: You are a 35 year old patient who is 12 weeks pregnant with your 2^nd^ child. Your pregnancy is going well, only some mild nausea that has been getting better every day. You eat a healthy diet, avoid alcohol, take pre-natal vitamins and try to stay active as you are very interested in the health of your unborn baby. However, you do continue to smoke. You successfully quit smoking during your last pregnancy about 5 years ago, with the “great” support of your family and friends. About 2 years ago, however, you started smoking again. You are considering quitting now, but you feel as though you need to smoke to relieve the stress of trying to balance your work and having to take your 5 year old son to the ER frequently due to “bad” asthma.

ROS: otherwise negative
PMH: G2P1 at 12 weeks gestation

Family History: sister is a former smoker, successfully quit “cold turkey” 10 years ago

Social History: you smoke about 10-15 cigarettes a day, more so when stressed from work, which seems to then always be followed by an ER visit due to an “asthma attack” of your 5 year old son. You currently work at a bank, and generally enjoy your work. However, you are worried that you might lose your job if you miss many more days due to your son’s illnesses.

Meds: prenatal vitamin

MI Information

- What are the patient’s life goals and values, especially with respect to the target behavior and overall health? Family? Work? Finances? You are interested in the health of your unborn baby as well as your 5 year old son. You also need to keep your very stressful job and are looking for ways to manage stress and create a better work-life balance.
- How does continuation of the target behavior fit in or conflict with these goals? Smoking helps you manage your stress but you acknowledge it is causing harm to your son and your unborn baby.
- What are the good things about the target behavior? How does it benefit you? Smoking helps you manage your stress.
- What are the less good things about the target behavior? What are you afraid might happen if you keep engaging in the target behavior? You are afraid of additional asthma attacks in your son, which you acknowledge can be exacerbated by second-hand smoke, and you hate to see him suffer. You also worry about losing your job if you have to miss any more work due to your son’s ER visits and illness. You also worry about how smoking could be affecting your pregnancy.
- What would be the advantages of making the behavior change? What are the best reasons to change? The health of your kids and not having to miss more work.
- Explore ways to make the change (pros and cons of various methods, what has worked in the past). You quit when you were pregnant with your first son, and you did this by chewing gum and cinnamon sticks instead of smoking. You went to yoga and practiced mindfulness meditation to manage your stress but back then it was easier to do this because you didn’t have kids. You need to find a gym with daycare.
- Importance and confidence of changing the target behavior:
  - How important is it for you to change? 10
    - Why is that number that high and not lower? You love your kids and don’t want to jeopardize their health, and you need to keep your job.
    - How could you move that number higher? Already the highest.
  - How confident are you that you can change? 8
    - Why is that number that high and not lower? You successfully quit before, and your sister quit, so you know you can do it.
    - What would make you more confident in your ability to change? If you found a better way to manage your stress, such as yoga and meditation, and if you found daycare so you could go to the gym.
- Ask for a decision. You will look for gyms that offer yoga and daycare, and you will look for a mindfulness meditation book/tape/app. You will set a quit date and buy gum and cinnamon sticks.
- Set SMART (specific, meaningful, assessable, realistic, timed) goals. What are the first steps? You will ask your friends about gyms with daycare and you will also research online. You will look for mindfulness meditation apps, books, and CD’s.
- What do you intend to do? You intend to set a quit date and explore other ways to manage your stress.

**Not getting recommended colonoscopy**

Presentation to Student:

Marie Brandt is a 52 year old patient you are seeing in the internal medicine clinic for a routine visit. She is feeling well and has no acute complaints. You see on chart review that she is overdue for her screening colonoscopy. She has a family history of colon cancer, so you are very concerned about her getting this done. She knows her risk of cancer is higher, but she is avoiding the procedure because she “doesn’t like the idea of it,” and after watching her mom get a partial colectomy, she is afraid of what they might find.

Information for the Standardized Patient:

History: You are a 52 year old patient with no medical problems being seen in clinic on a routine follow up. Your doctor saw in your chart that you have yet to get your screening colonoscopy completed. You have been putting this off for a few reasons. You generally “don’t like the idea of it,” meaning the sensitive nature of the procedure “kind of grosses me out.” You have a friend who had a colonoscopy last year, and had horror stories about drinking the prep and “all the diarrhea” they had. In addition, your mom was diagnosed with colon cancer and had to get part of her colon removed, and you are afraid of what they might find, and you needing to get surgery. You admit, however, that your mom’s cancer was late in getting diagnosed, and you remember the doctors telling her that it could have been caught earlier with a colonoscopy and she could have possibly avoided surgery.

ROS: otherwise negative
PMH: none

Family History: mom diagnosed with colon cancer at 65 after complaining of abdominal pain and having a CT scan done. She needed to get a partial colectomy as part of her treatment.

Social History: no tobacco, alcohol or drug use.

Meds: daily multivitamin

MI Information

- What are the patient’s life goals and values, especially with respect to the target behavior and overall health? Family? Work? Finances? Patient wants to be healthy and avoid getting colon cancer like her mother.
- How does continuation of the target behavior fit in or conflict with these goals? The longer you wait the more complicated the treatment might be if you do have cancer.
- What are the good things about the target behavior? How does it benefit you? Avoiding screening allows you to avoid the diarrhea from the prep and also avoid the sensitive nature of the test. Avoidance also allows you to not have to worry about having cancer (negative reinforcement of the fear in the short run).
- What are the less good things about the target behavior? What are you afraid might happen if you keep engaging in the target behavior? You are afraid you might end up like your mother, who had to get surgery because her colon cancer was not caught earlier.
- What would be the advantages of making the behavior change? What are the best reasons to change? Getting the screening and either learning you don’t have colon cancer (a huge relief), or catching it early and possibly getting to avoid surgery as a treatment.
- Explore ways to make the change (pros and cons of various methods, what has worked in the past). Weigh out the short term cost of a sensitive procedure versus the long term gain of either learning that you are cancer free or that you have caught it early.
- Importance and confidence of changing the target behavior:
  - How important is it for you to change? 6
    - Why is that number that high and not lower? You really don’t want to have surgery.
    - How could you move that number higher? Weigh out the pros and cons of getting screened, talk with your mother about her cancer and surgery.
  - How confident are you that you can change? 4
    - Why is that number that high and not lower? You have done difficult things in the past, and you went through labor that was fairly sensitive and a bit messy.
    - What would make you more confident in your ability to change? Maybe having a trusted friend or family member present at the appointment, or having a medication that sedates you during the procedure.
- Ask for a decision. You will get a colonoscopy.
- Set SMART (specific, meaningful, assessable, realistic, timed) goals. What are the first steps? You will make a pros and cons list, you will talk with your mom, and look into getting an appointment in the next week.
- What do you intend to do? Get started on the goals above.

**Compliance with a Walker**

Presentation to Student:

Milton (Mildred) Jones is a 75 year old male/female brought to the ER by his/her daughter, due to a ground level fall at home. He/she has been seen multiple times this year for falling episodes and had an extensive workups. He/she has worked with physical therapy and has been advised to use a walker at home for safety and fall prevention. He/she does not have any fractures or injuries from his/her fall.

Information for the Standardized Patient:

You are a 75 year old patient, brought to the ER by your daughter due to a falling episode at home. You have fallen at home about 5 times this year. You have not had any fractures, but the falls are becoming more frequent. Though your daughter lives right next door to you, you feel very independent. You love taking care of your grandkids and socializing with your friends. You recall having sessions with physical therapy and the strong recommendation to use a walker. You have not used your walker but you have it at home. You feel that the walker makes you lose your independence and makes you feel “old.” Following discussion about the risks of falling and possible complications, you are motivated to begin to use the walker for your safety. You have a friend who fell, broke her hip, and ended up having to use a wheelchair, and you don’t want that to happen to you.

PMH: frequent falls

Meds, none. Allergies, none. Family history, none.

Social history: retired accountant, living next door to daughter, non-smoker, no alcohol intake, exercise on a stationary bike daily for 20 minutes, many hobbies during the day including gardening and spending time with friends, taking care of your grandkids.

MI Information

- What are the patient’s life goals and values, especially with respect to the target behavior and overall health? Family? Work? Finances? You are fiercely independent and have trouble accepting the aging process. You love your daughter and grandkids and want to be able to continue providing care for them. You also love socializing with your friends, gardening, and your other hobbies, and you want to be able to continue these.
- How does continuation of the target behavior fit in or conflict with these goals? It allows you to feel more independent and avoid the reality of your aging, but it conflicts with your goals around family, friends, and recreation because if you break bones in a bad fall you may not be able to continue with these valued directions.
- What are the good things about the target behavior? How does it benefit you? Not using the walker allows you to feel independent and young.
- What are the less good things about the target behavior? What are you afraid might happen if you keep engaging in the target behavior? You are afraid of a bad fall, breaking bones, and ending up in a wheelchair, which would really limit your independence.
- What would be the advantages of making the behavior change? What are the best reasons to change? Your grandkids are the best reason to change, and begin able to continue to care for them. You also value your health, mobility, friends, and hobbies.
- Explore ways to make the change (pros and cons of various methods, what has worked in the past). Realizing that true independence comes from using the walker and avoiding a wheelchair or nursing home. It might help to have notes around the house to remind you to use your walker. Maybe you could have an extra walker in the car for use when you’re out. Maybe you could consider other assistive devices such as a cane or having railings in the home.
- Importance and confidence of changing the target behavior:
  - How important is it for you to change? 4/10
    - Why is that number that high and not lower? You really care about your grandkids but you are ambivalent because you are a little vain (you don’t want others to think you are old) and fiercely independent so you don’t like to use your walker.
    - How could you move that number higher? Maybe keep a picture of your grandkids handy to remind yourself of your values. Maybe visiting a nursing home to create relativity (it is not great to use your walker but you’re happy to have some mobility).
  - How confident are you that you can change? 6/10
    - Why is that number that high and not lower? If you decide to use the walker, it won’t be that hard to remember to use it regularly.
    - What would make you more confident in your ability to change? If you realized that people won’t think you are old for using your walker, if you realized that using the walker will actually help you keep your independence and not lose it.
- Ask for a decision. You decide to use your walker, for the sake of your grandkids.
- Set SMART (specific, meaningful, assessable, realistic, timed) goals. What are the first steps? You will put notes around the house and ask your daughter and grandkids to remind you to use it. You will purchase a second one to keep in your car.

**Lack of Exercise**

Presentation for the student:

Michael Acres is a 33 year old male presenting to the primary care clinic today for evaluation of “being tired all the time.” He has had a thorough workup, which has all been negative. He denies depression. He consumes a healthy diet but does not exercise at all, something that the endocrinologist you referred him to strongly recommended he try to increase his energy. He is a free-lance writer but has not written anything in some time, and spends most of his day “getting caught up on his rest.”

Vitals are normal.

Information for the SP:

You are a 33 years old patient, presenting to urgent care for answers about why you are tired all of the time and have gained 10 lbs. in the past 3 months. You have had a workup and have been recommended in the past to increase your activity level. You currently do not exercise. You also feel that you are gaining weight and your confidence and motivation to go and do things are being replaced with wanting to stay in bed. You have modified your diet and are trying to make healthier food choices, but this has not improved your energy level. You would like to have more energy and when you learn that an exercise regimen may boost your energy level, you are very open to trying that. You do not have other medical problems and you do not take any medications.

PMH: none, no medications, no allergies

Family history: none

Social history: denies alcohol, no smoking, not exercising, working on healthy diet, living alone. You have access to a gym in your apartment complex. You are not currently working.

MI Information

- What are the patient’s life goals and values, especially with respect to the target behavior and overall health? Family? Work? Finances? You would love to have more energy so you could get back to your writing and artwork (pottery). You need to publish something soon so you can pay your rent.
- How does continuation of the target behavior fit in or conflict with these goals? Lying in bed or lounging on the couch make you more tired, and they sap your motivation to do any creative activity. You don’t understand that exercise will improve your energy so you need your doctor to explain this to you.
- What are the good things about the target behavior? How does it benefit you? Lying in bed is so easy, and you don’t have to deal with your writer’s block.
- What are the less good things about the target behavior? What are you afraid might happen if you keep engaging in the target behavior? You don’t like your lack of energy and you are afraid you’ll keep gaining weight. You miss writing and your art work. You are worried you won’t be able to pay the rent if you don’t publish something soon.
- What would be the advantages of making the behavior change? What are the best reasons to change? More energy would allow you to write and work on your pottery. You might stop gaining weight and may even lose weight.
- Explore ways to make the change (pros and cons of various methods, what has worked in the past): get an exercise buddy, use the apartment gym, start slow and work up, walk, exercise videos, etc.
- Importance and confidence of changing the target behavior:
  - How important is it for you to change? 4/10
    - Why is that number that high and not lower? You miss your creative outlets and you don’t want to keep gaining weight.
    - How could you move that number higher? You could buy some exercise clothes and put them on each morning, to increase your motivation to go exercise.
  - How confident are you that you can change? 6/10
    - Why is that number that high and not lower? You used to exercise when in college, and you remember having more energy then.
    - What would make you more confident in your ability to change? Having an exercise buddy would really help.
- Ask for a decision: You will get some exercise clothes, you will look for an exercise buddy, you will buy and try an exercise video.
- Set SMART (specific, meaningful, assessable, realistic, timed) goals. What are the first steps? You will try to exercise for at least 30 minutes 3 times a week to start.
- What do you intend to do? Take the steps above.
