# Supplementary material for: Motivational Interviewing Training: A Case-Based Curriculum for Preclinical Medical Students
Source: MedEdPORTAL. 2021 Feb 12;17:11104. doi: 10.15766/mep_2374-8265.11104 (PMC7880250; doi:10.15766/mep_2374-8265.11104)
Supplement: Supplementary file 1 — Presurvey.docxMI Presentation.pptxMI Demonstration Script.docxTransparent Outline for MI Activity.docxMICA Evaluation Tool.docPractice Cases.docxMI Summary Sheet.docxEvaluated Cases.docxOARS Tracking Sheet.docChange Talk Tracking Sheet.docMI Evaluated Session Sample Schedule.xlsxActing Patient Experience Scale.docxPostsurvey.docxFacilitator Guide.docx [file mep_2374-8265.11104-s001.zip › G. MI Summary Sheet.docx]

**Motivational Interviewing Summary**

General Principles

- Resist the “righting reflex”
- Express empathy
- Develop discrepancy
- Roll with resistance
- Support self-efficacy (past successes)
- Avoid argumentation

Specific MI Strategies and Skills

- Ask open-ended questions
  - What are the positive and less good things about (your behavior)?
  - What will you lose if you give up (your behavior)?
  - What are you afraid could happen if you keep engaging in (your behavior)?
- Listen reflectively (paraphrase, hypothesize)
- Affirm (appreciate)
- Summarize
- Elicit self-motivational statements
- Ask-provide-ask
  - Can we talk about (your behavior)? – Provide information – Ask what they think about what you just said
- Assess readiness to change
- Elicit change talk
- Sharpen the focus
  - Break behavior to change down into small components
  - Write down the regimen
- SMART goals

Other Good Questions To Ask in MI

- Can we talk about your (your behavior)?
- What are your goals for your health? Family? Work? Finances?
  - What would you like your life to be like 5 years from now?
- How does continuation of (your behavior) fit in with these values?
- What would be the good things about quitting (your behavior)?
- What worries you about (your behavior)? What problems have you had so far?
- What do you think will happen if you don’t quit (your behavior)?
- How important is it for you to change (your behavior)?
  - Why is the number that high and not lower?
- How confident are you that you can change (your behavior)?
  - Why is the number that high and not lower?
- What change strategies do you think would work for you?
- What has worked for you in the past to change behaviors?
- What would you be willing to try?
- What do you intend to do?
