# Supplementary material for: Motivational Interviewing Training: A Case-Based Curriculum for Preclinical Medical Students
Source: MedEdPORTAL. 2021 Feb 12;17:11104. doi: 10.15766/mep_2374-8265.11104 (PMC7880250; doi:10.15766/mep_2374-8265.11104)
Supplement: Supplementary file 1 — Presurvey.docxMI Presentation.pptxMI Demonstration Script.docxTransparent Outline for MI Activity.docxMICA Evaluation Tool.docPractice Cases.docxMI Summary Sheet.docxEvaluated Cases.docxOARS Tracking Sheet.docChange Talk Tracking Sheet.docMI Evaluated Session Sample Schedule.xlsxActing Patient Experience Scale.docxPostsurvey.docxFacilitator Guide.docx [file mep_2374-8265.11104-s001.zip › H. Evaluated Cases.docx]

**Evaluated Cases**

Note – Age and gender of the SP can be changed to match availability in your SP pool, unless the age/gender are relevant to the case.

**Smoking Cessation Case**

Presentation to Student:

John Smith, a 60-year-old male, with severe COPD and ongoing nicotine dependence, presents to the clinic for an acute care visit. He is having more difficulty with breathing over the past week, even though he just completed a course of steroids and antibiotics for a COPD exacerbation at the end of last month.

Vitals normal, oxygen saturation is low on room air and patient has wheezing on exam.

Information for the Standardized Patient:

65 years old

History: Feeling difficulty with breathing, especially with any exertion, such as walking up stairs or walking to do errands. Difficult to catch breath, no chest pain or tightness. No fevers or any other symptoms, but you feel similarly to last month when you required a course of steroids and antibiotics. You did feel better after the course of treatment, but feel that the same symptoms are returning again. You are very frustrated as this happens over and over again, and these episodes seem to be happening more frequently.

You have been smoking since age 15 years, at least one pack of cigarettes daily. You are beginning to get serious about smoking cessation because these exacerbations of COPD have been difficult to overcome. However, you have a very stressful job and smoking helps you to relax.

ROS: negative for all systems reviewed

PMH: no other medical problems

Family history: none

Social history: smoking since age 15, 1 pack per day, no alcohol or drug use, living alone, working at a very stressful job as an accountant

No medications or allergies

Information for SP to respond to MI questions:

- Life goals and values: I’d like to have better health, be able to do my daily activities without getting so tired, not be so stressed out all the time.
- How does continuation of the target behavior fit in or conflict with these goals? It conflicts with my values, but I really like smoking because it helps me relax.
- What are the good things about smoking: It helps me calm down when I’m stressed out, and it helps me manage my boredom since I live alone.
- What are the bad things about smoking: I’m getting really frustrated with these exacerbations and it is getting harder to do daily activities without getting winded. I’m too young to have COPD and if I get worse I might need to hire someone to help me with my daily activities. It is frustrating to have to keep going to the doctor about this.
- What would be the advantages of making the behavior change? What are the best reasons to change? I wouldn’t have these episodes and exacerbations, I wouldn’t feel so winded when I do my daily activities, I’d have more energy and I’d feel better. My health would be better overall.
- Explore ways to make the change (pros and cons of various methods, what has worked in the past): Patches, Chantix, using a self-help book, calling a smoking cessation hotline, using online resources, going to a stop smoking group. You tried quitting cold turkey 4-5 times but that only lasted a few days or a couple of weeks at best. You really had trouble managing the cravings and ended up relapsing each time.
- Importance and confidence of changing the target behavior:
  - How important is it for you to change? 7/10
    - Why is that number that high and not lower? I’m just so sick and tired of being out of breath and having these episodes!
    - How could you move that number higher? If there were another way for me to deal with my stress and boredom, I think I’d say 10/10.
  - How confident are you that you can change? 6/10
    - Why is that number that high and not lower? I’ve been through some really difficult things in my life, and I know if I stick my mind to it I can do it.
    - What would make you more confident in your ability to change? I think the cold turkey approach didn’t work because my cravings got the better of me. Maybe if I had some help, like a patch and some information, I’d feel more confident.
- Ask for a decision: I’m going to look into different ways to quit, like patches and medication and information.
- Set SMART (specific, meaningful, assessable, realistic, timed) goals. What are the first steps? I need to find some websites to get information about ways to quit smoking, and I can ask the doctor for recommendations. I will talk with my friends about their advice for dealing with boredom and stress. I would like to set a follow up appointment to meet in a month to discuss quit aids and activities I have decided on trying~~.~~
- What do you intend to do? I will do this in the next month.

**Alcohol Abuse Case**

Presentation to the Student:

Elle Frank is a 50-year-old female, presenting to your clinic for a routine follow-up visit. She has been continuing to consume alcohol on a daily basis. Repeat lab work prior to the visit reveals that markers of alcohol related liver damage are elevated slightly from prior laboratory data.

For the SP:

History: You are a 50-year-old patient. You have been drinking about a six-pack of beer on a daily basis. On days when you are feeling more stress, you are also consuming about 2 or 3 mixed drinks in addition, happening about 1-2 times per week. You do not have history of drug use and you quit smoking 10 years ago. You are currently unemployed after losing your job for having alcohol on your breath repeatedly, have one DUI from last New Year’s Eve, and you notice mild withdrawal symptoms (trembling, headaches) when you stop drinking alcohol. You started to drink heavily because that is what everyone did together after work, but now you drink to deal with your boredom and depression. You have a support network of family members and friends, who are reminding you and urging you to stop drinking alcohol also. You feel that drinking alcohol has become an important part of your day, and following discussion with your doctor, you would like to find the motivation to stop drinking to be able to find work again. You are becoming concerned about your “liver tests” that have been worsening since your last visit 2 months ago.

PMH: high blood pressure, taking a blood pressure medication (amlodipine) daily at home, no allergies

Family history: Father died of alcoholic cirrhosis

Social history (see above)

Information for SP to respond to MI questions:

- What are the patient’s life goals and values, especially with respect to the target behavior and overall health? Family? Work? Finances? You are concerned about your liver tests and what that might mean for your health, you don’t want to turn out like your alcoholic father, you don’t want to get a second DUI because that would mean a mandatory 30 days in jail, and you really want to return to work.
- How does continuation of the target behavior fit in or conflict with these goals? Continuing to drink conflicts with all of these goals.
- What are the good things about the target behavior? How does it benefit you? It used to help you socialize after work, manage the stress of the day, and fit in with friends when you were working. Now it helps you to manage your boredom and depression since you are out of work.
- What are the less good things about the target behavior? What are you afraid might happen if you keep engaging in the target behavior? You really don’t want to turn out like your alcoholic father and die of cirrhosis, you don’t want another DUI and end up in jail, and you don’t want to lose another job because of your drinking. You also realize that drinking makes your depression worse in the long run, even though it helps you in the short run.
- What would be the advantages of making the behavior change? What are the best reasons to change? You would have a better chance at getting and keeping a job, which would help with your boredom and depression, you wouldn’t have to worry about getting another DUI, your health would improve, and you would be less likely to turn out like your dad.
- Explore ways to make the change (pros and cons of various methods, what has worked in the past): You went to one AA meeting ~~but you were unsure about the whole “God” part of AA,~~  and you might be willing to try it again. You can’t afford therapy or medications to help curb cravings. Once when you had a non-drinking girlfriend (or boyfriend) you quit drinking through her (his) support and spending time with her (him).
- Importance and confidence of changing the target behavior:
  - How important is it for you to change? 6/10
    - Why is that number that high and not lower? You’re so tired of feeing this way, you really want to work because that would make so much of your problems better, and you don’t want to further damage your liver.
    - How could you move that number higher? If you better understood what effect alcohol is having on your liver and moods.
  - How confident are you that you can change? 4/10
    - Why is that number that high and not lower? You were able to quit drinking before when you were with a nice partner, so you know you can do it.
    - What would make you more confident in your ability to change? If you had better support, friends who don’t drink, had medications for your depression, had help finding work, and tried AA again.
- Ask for a decision: You agree to try an antidepressant and to go to several different AA meetings.
- Set SMART (specific, meaningful, assessable, realistic, timed) goals. What are the first steps? You will go to 5 different AA meetings in the next week and try socializing with AA members who do not drink. You will start a trial of antidepressants and follow up with your doctor in 3 weeks.
- What do you intend to do? Try AA and antidepressants.

**Noncompliance with Medication Regimen Case**

Presentation for the Student:

Roberta Stanley is a 67-year-old female presenting to the clinic today for a new patient visit. She has prior history of hypertension, but has not seen a physician in about 2 years. Her very close friend recently had a large stroke at the age of 68 years old, and remains in a rehab facility.

Patient’s blood pressure today is 178/92 and has not been taking blood pressure medication regularly.

Information for the SP:

You are a 67-year-old patient and are coming to the clinic for follow-up. You have not seen a doctor in a couple of years and have not been taking any blood pressure medications. You were taking Lisinopril (40 mg daily) previously for your blood pressure control, but you were feeling well and stopped taking the medicine when life “became busy.” You still work, helping a friend in an office setting. You do not have any other known medical problems, and you do not have any issues today during clinic (no headaches, chest pain, fevers, etc). When asked, you are a bit concerned because your friend of 30 years just had a large stroke and though she is in a rehab facility, she has quite severe deficits. You are willing to consider taking Blood pressure medications regularly, even though you feel no symptoms without the medications, when you learn that high blood pressure is a risk factor for stroke.

PMH: high blood pressure, previously prescribed Lisinopril 40 mg daily, not taking

Family history: unknown, adopted

Social history: you have never smoked, no alcohol intake, live with spouse, working in office setting

Allergies, none

Information for SP to respond to MI questions:

- What are the patient’s life goals and values, especially with respect to the target behavior and overall health? Family? Work? Finances? Patient wants to avoid having a stroke like her friend. She wants to control her blood pressure and continue to work and enjoy her family.
- How does continuation of the target behavior fit in or conflict with these goals? Noncompliance with blood pressure medications will put you at risk for having a stroke.
- What are the good things about the target behavior? How does it benefit you? Not taking meds for hypertension allows you to believe that you are healthy and don’t need to take medications (taking the medicine is a reminder that you have HTN), you are “busy” and find it hard to fit taking medications into your schedule.
- What are the less good things about the target behavior? What are you afraid might happen if you keep engaging in the target behavior? You are very afraid of ending up like your friend with the stroke. You do not want to have the severe deficits that have resulted from your friend’s stroke.
- What would be the advantages of making the behavior change? What are the best reasons to change? You can manage your hypertension and avoid having a stroke. You can be around for your spouse/family and be able to continue working.
- Explore ways to make the change (pros and cons of various methods, what has worked in the past): You could try a pill box, setting a reminder on your phone, putting up reminder notes, pairing pill taking with another regular behavior like brushing your teeth, taking your blood pressure regularly to remind you of your HTN, etc.
- Importance and confidence of changing the target behavior:
  - How important is it for you to change? 9/10
    - Why is that number that high and not lower? You really don’t want to have a stroke like your friend.
    - How could you move that number higher? Maybe keep a picture of your friend by my pill bottle, to remind you of what could happen if you don’t take my medications.
  - How confident are you that you can change? 5/10
    - Why is that number that high and not lower? I think the strategies we discussed will help.
    - What would make you more confident in your ability to change? Maybe get your husband involved in reminding you? Get a blood pressure cuff at home to take your own pressures regularly.
- Ask for a decision: You will try a pill box with calendar reminders and getting your husband to help remind you.
- Set SMART (specific, meaningful, assessable, realistic, timed) goals. What are the first steps? You will leave the office and buy a pill box and home cuff, you will set calendar reminders in your phone, and ask your husband to help you remember.
- What do you intend to do? Take the above steps.

**Sodium and fluid intake Case**

Dolores Case is a 50-year-old female presenting to the clinic for a hospital follow-up visit. She has a known history of congestive heart failure with an Ejection fraction of 25% and takes her medications as directed. She is feeling discouraged because she has been hospitalized 4 times this year for fluid overload, and is so tired of being in the hospital. During each hospitalization she is reminded and counseled regarding the importance of limiting salt and watching fluid intake closely to prevent re-hospitalization due to fluid overload. She is able to restrict fluids but is struggling with limiting sodium. Vitals are stable.

Information for the SP:

You are a 50-year-old patient with a history of congestive heart failure. You were diagnosed about 6 years ago, and the heart failure is due to alcohol use, which you quit at the time the heart failure was diagnosed. You have maintained your follow up visits and take all of your medications as directed. You do not miss doses of your medications. You had a defibrillator placed 5 years ago also, and this has not had any issues. You do not smoke. You have been told by the hospital doctors to monitor your salt and fluid intake, but you rely on TV dinners most nights also. You are compliant with fluid restrictions. After a discussion about monitoring of salt intake, you are motivated to hear that this factor may greatly reduce hospitalizations due to your heart failure.

PMH: CHF with ejection fraction of 25%, AICD placed 5 years ago

Medications: (should be handed in a small list) Lasix 20 mg daily, Lisinopril 20 mg daily, Coreg 6.25 mg twice daily

No allergies, no medication problems or recent changes, taking meds as directed

Family history: none

Social history: history of alcohol intake, quit completely 6 years ago, no history of drug use, lives with spouse, continue to work at office supply store

Information for SP to respond to MI questions:

- What are the patient’s life goals and values, especially with respect to the target behavior and overall health? Family? Work? Finances? You are very tired of being hospitalized for fluid overload and would like to avoid future hospitalizations, as they interfere with your work and you are afraid of losing the job you love and that pays the bills. You have been compliant with all other treatment recommendations and truly value your health, but you don’t understand how excessive salt contributes to your CHF exacerbations.
- How does continuation of the target behavior fit in or conflict with these goals? Excessive salt and fluid intake conflict with all of the above goals, but eating TV dinners is easy because you and your spouse don’t cook. You are also worried about your spouse’s salt intake, as s/he has hypertension.
- What are the good things about the target behavior? How does it benefit you? It is easy to make TV dinners since you and your spouse don’t cook.
- What are the less good things about the target behavior? What are you afraid might happen if you keep engaging in the target behavior? You are afraid of continued hospitalizations and worsening of your CHF. You are afraid of losing your job if you have to take much more sick leave. You also worry about your spouse consuming so much salt.
- What would be the advantages of making the behavior change? What are the best reasons to change? You would have improved health and lower your chance of losing your job. You would avoid future hospitalizations for CHF exacerbations.
- Explore ways to make the change (pros and cons of various methods, what has worked in the past): You could take a cooking class with your spouse and try making home cooked meals at least a few times a week. You could track your salt intake. Your spouse helped you to quit drinking and be compliant with your medications, so he/she could help you with these modifications.
- Importance and confidence of changing the target behavior:
  - How important is it for you to change? 8/10
    - Why is that number that high and not lower? You really hate the hospital and if you lost your job, you would not be able to pay your bills. You also worry about your health and that of your spouse.
    - How could you move that number higher? You could remind yourself of your hospitalizations each time you contemplate having salt or TV dinners.
  - How confident are you that you can change? 7/10
    - Why is that number that high and not lower? You quit drinking when you were diagnosed with heart failure and you are complaint with medications, so you know you can make changes.
    - What would make you more confident in your ability to change? Knowing how to cook, so you don’t eat TV dinners with so much salt.
- Ask for a decision: You will look into cooking classes and talk to your spouse about it, you will get an app that lets you track how much sodium you have consumed.
- Set SMART (specific, meaningful, assessable, realistic, timed) goals. What are the first steps? You know of a cooking school so you will go there this week and look into classes; you will download the sodium tracker app and start using it this week.

**Compliance with anticonvulsants Case**

Presentation to student:

Frances Peters is a 44-year-old female with history of seizure disorder, diagnosed over 20 years ago after a TBI. Patient is brought to ER after witnessed seizure episode at home by her friend. Patient’s vitals are stable, but her Dilantin level is 0.0 as checked in the Emergency room. When patient recovers from seizure episode and has been given her dosage of Dilantin, she is concerned when she discovers her license will be revoked until her seizures are in control, as she uses her car frequently to drive to and from school and work.

Information for the SP:

You are a 44-year-old patient, with a long history of seizure disorder since a brain injury many years ago. You have had seizures off and on for about 20 years, some years only one seizure, some years as many as 4 to 5. You forget to take your medications on occasion, and find it difficult to fill the prescription for your Dilantin every month without forgetting. You are very concerned that your license has been taken away by the ER physician, but you understand that is the policy to prevent risk to yourself and others while driving. Once you learn that you may reestablish and maintain your license if your seizures are controlled, you focus on ways to increase your compliance with the medication.

PMH: TBI 20 years ago after falling from a ladder

Seizure disorder, well controlled on Dilantin 300 mg daily

No allergies, no family history

Social history: nonsmoker, no alcohol intake ever, you are going back to school taking evening classes and working during the day as a waiter/waitress, you have a spouse and 2 young kids

Information for SP to respond to MI questions:

- What are the patient’s life goals and values, especially with respect to the target behavior and overall health? Family? Work? Finances? You would like to stop having seizures, be healthy for your spouse and kids, get your license back and drive again so you can get to work and school, and get a degree so you don’t have to work as a waiter/waitress anymore.
- How does continuation of the target behavior fit in or conflict with these goals? Noncompliance conflicts with your value of controlling your seizures and getting your license back. It could also be dangerous if you had a serious seizure and you don’t want to worry your family.
- What are the good things about the target behavior? How does it benefit you? Your seizures are so rare that it is difficult to remember to take your medications. You don’t like to take your medications because it reminds you that you have seizure disorder, and you have already suffered so much with your TBI.
- What are the less good things about the target behavior? What are you afraid might happen if you keep engaging in the target behavior? You are afraid you won’t be able to get your license back, you don’t want to worry your family, you don’t want to have more seizures.
- What would be the advantages of making the behavior change? What are the best reasons to change? Your health, your license back, you not worrying your family.
- Explore ways to make the change (pros and cons of various methods, what has worked in the past): Get prescriptions mailed to you, get a pill box, put your license next to your pills (to give you motivation to get your license back), set your phone to remind you to take your medications, get your spouse to help you remember to take your medications, pair pill taking with another established behavior (like brushing teeth).
- Importance and confidence of changing the target behavior:
  - How important is it for you to change? 9/10
    - Why is that number that high and not lower? You really need your license back so you can get to school and work, you value your health and don’t want to worry your family
    - How could you move that number higher? Get some support to deal with the reality of your seizure disorder diagnosis, since you don’t like to remember that you have it when you take your medications. Perhaps a support group?
  - How confident are you that you can change? 3/10
    - Why is that number that high and not lower? Your spouse is helpful in reminding you of things, but it is hard to remember to take a medication when you have such few seizures a year.
    - What would make you more confident in your ability to change? If the doctor talked to your spouse to get him/her on board in helping you take your medications.
- Ask for a decision: You will take your medications as prescribed.
- Set SMART (specific, meaningful, assessable, realistic, timed) goals. What are the first steps? You will go with your spouse for an appointment with your outpatient doctor tomorrow, you will get a pill box and set reminders on your phone, you will get autofill prescriptions or set renewal with other monthly activities like paying a bill.

**Diabetic and Noncompliant with Insulin**

Presentation to the Student:

Mark Mitchell is a 45-year-old male presenting to your clinic today for a follow-up visit for diabetes. He is worried about his diabetic ulcer today. He has been playing basketball with his 10 year old son, and lately has not been able to participate because of an ulcer that has developed on his foot. When reviewing his blood sugar log, his blood sugars are high throughout the day, and his last HgA1c was 10.5 (this indicates that his daily blood glucose readings average 250. His goal HgA1c is <7). He takes his insulin “when he can remember to take it,” which is not every day.

Information for the SP:

You are a 45-year-old patient, who has diabetes. You do not take your insulin regularly, and have had poorly controlled diabetes for years. You take your insulin only when you remember, but it is difficult to remember to take it every day since you do not feel different or badly when you do not take your insulin. You have been frustrated lately because you like to play basketball with your 10 year old son, and haven’t been able to play basketball because you have developed an ulcer on your right foot (as a result of poorly controlled diabetes).

Medical history: diabetes, diagnosed 4 years ago

Medications: Lantus insulin daily 20 units at night (you are taking this only a few times weekly) and Lisinopril 10 mg daily (blood pressure medicine you were told to take because of the diabetes)

Allergies: none

Social history: you do not smoke, drink alcohol or use any drugs

Family history: father with diabetes

Information for SP to respond to MI questions:

- What are the patient’s life goals and values, especially with respect to the target behavior and overall health? Family? Work? Finances? You want to be able to play basketball with your son again, and you want to get rid of the foot ulcer. You really love your son and you love to play basketball.
- How does continuation of the target behavior fit in or conflict with these goals? Noncompliance with insulin can lead to complications like your foot ulcer, but you will need the doctor to help you make that connection and to describe other possible complications. You cannot play basketball with your foot ulcer. You are also not taking your blood pressure medication as prescribed, and this could also result in complications.
- What are the good things about the target behavior? How does it benefit you? It is easy to “forget” to take your medications. At night you are very tired and sleepy from your day, so you tend to fall asleep while watching TV, and don’t take any medications before you fall asleep. Also, since you don’t “feel” your high sugars or blood pressure, there are no symptoms/reminders for you to take your medications. You like to think of yourself as “young and healthy,” and taking medications interferes with this way of looking at yourself.
- What are the less good things about the target behavior? What are you afraid might happen if you keep engaging in the target behavior? You are afraid you might not be able to keep playing basketball with your son. Once the doctor describes possible complications from nonadherence with diabetes, you are afraid of those.
- What would be the advantages of making the behavior change? What are the best reasons to change? Your son, and ability to continue playing basketball with him; improved health.
- Explore ways to make the change (pros and cons of various methods, what has worked in the past): Maybe you could take the medication at a different time of the day, or at least before you fall asleep watching TV. Maybe you could challenge your thoughts that people who take medications are not young or healthy. You could try using reminders, pill boxes, etc. You could test your sugars regularly to remind yourself of possible complications (since you don’t “feel” the high sugar levels).
- Importance and confidence of changing the target behavior:
  - How important is it for you to change? 6/10
    - Why is that number that high and not lower? You love your son and love to play basketball with him. You fear other complications like an amputation.
    - How could you move that number higher? Maybe put a picture of your son or a basketball on the pill bottle.
  - How confident are you that you can change? 5/10
    - Why is that number that high and not lower? You exercise even when you are tired, so you believe you could take medications even if you are tired at night.
    - What would make you more confident in your ability to change? Understanding the links between noncompliance and complications. Perhaps your son could help you to remember to take your medications.
- Ask for a decision: You will take your insulin and blood pressure medications.
- Set SMART (specific, meaningful, assessable, realistic, timed) goals. What are the first steps? You will get a pill box. You will ask your son to agree not to play basketball with you unless you have taken your medications.

**Pregnant with Noncompliance of Prenatal Care** (childbearing aged female SP only)

Information for the student:

Brenda Smithson is a 34-year-old pregnant female who presents to you, the family practitioner, for scheduled well-child check for her healthy 2-year-old daughter, Ellie. During this visit, she mentions to you that she is pregnant again. During conversation, she also mentions that she has not been as good at going to prenatal care OB appointments or taking her prenatal vitamins, since this is her second pregnancy, she gets busy with her toddler, and she finds herself running out of time and energy in the day.

Information for the SP:

You are a pregnant patient, bringing your 2-year-old girl, Ellie, in for her routine visit to your family doctor. You mention to the doctor that you haven’t been as vigilant about going to prenatal visits or taking your prenatal vitamins because you are busy with your daughter. When your family doctor discusses the importance of prenatal care and taking the prenatal vitamins to ensure that the pregnancy is healthy, just as it was with Ellie, you are motivated to attend appointments and begin taking the medication every day just as you did in the previous pregnancy.

Medical history: none

Medications and allergies: occasional prenatal vitamin, otherwise no other medications

Family history: none

Social history: nonsmoker, no alcohol intake, no drug use, currently working at home taking care of your toddler, no regular exercise

Information for SP to respond to MI questions:

- What are the patient’s life goals and values, especially with respect to the target behavior and overall health? Family? Work? Finances? You really love your daughter, but would like to have another healthy baby.
- How does continuation of the target behavior fit in or conflict with these goals? You need education that failure to comply with prenatal care, including visits and vitamins, could lead to problems with your pregnancy and the baby.
- What are the good things about the target behavior? How does it benefit you? It is a hassle to worry about prenatal care, and you would rather spend time with Ellie. You feel that since the first pregnancy was fine, this one should be fine too.
- What are the less good things about the target behavior? What are you afraid might happen if you keep engaging in the target behavior? Once the doctor explains possible complications from nonadherance, you are afraid of having those complications in your current pregnancy.
- What would be the advantages of making the behavior change? What are the best reasons to change? You want another healthy baby.
- Explore ways to make the change (pros and cons of various methods, what has worked in the past): You could get a babysitter for your toddler so you can come to appointments, or you might be able to bring her. You could swap babysitting services with a friend so you can attend appointments with your OB. To remember to take the vitamins, you could use a pill box, pair pill taking with another established behavior like teeth brushing, set a reminder on your phone, etc.
- Importance and confidence of changing the target behavior:
  - How important is it for you to change? 10/10
    - Why is that number that high and not lower? You don’t want to be responsible for having a baby with problems that could have been prevented.
    - How could you move that number higher? Can’t move it any higher, but further education about prenatal care and vitamin benefits could help you understand their importance.
  - How confident are you that you can change? 7/10
    - Why is that number that high and not lower? You really want a healthy baby, and you have been able to cut out alcohol during this pregnancy.
    - What would make you more confident in your ability to change? Having a plan, getting education about prenatal vitamins, setting up reminders.
- Ask for a decision: You will start taking your prenatal vitamins daily and set up OB appointments.
- Set SMART (specific, meaningful, assessable, realistic, timed) goals. What are the first steps? You will get a pill box, set reminders on your phone, call your OB to make appointments for the next few months.

**Recurrent STD with Continued Unprotected Sex**

(Female SP only)

Introduction for the Student:

Bridget Cooper is a 50-year-old heterosexual female presenting to the gynecologist for STD screening. She has had prior sexually transmitted infection screenings in the past and despite being counseled about safe sex practices, has not been using condoms with her partners. She has had 2 partners in the past year and denies any symptoms. She had a partial hysterectomy due to fibroids 12 years ago and is up to date on her PAP smear, colonoscopy and mammogram (all normal). Vital signs are normal. Additionally, she recently had a friend diagnosed with HIV, and therefore would like to have an HIV test also.

Information for the Standardized Patient:

You are a healthy female patient, presenting to the gynecologist for sexually transmitted infection testing. You have had a hysterectomy because of “fibroids” (no cancer) about 12 years ago, so you have not had a menstrual period since that time. You are up to date on your PAP smears and other health screenings, such as mammograms and colonoscopies. You and your gynecologist have had multiple discussions about safe sex practices, but at this time you continue to not use condoms for protection. You do not have any symptoms and have had 2 sexual partners in the past year since your last screening. Prior to that, you cannot remember the number of sexual partners you have had exactly. You are especially concerned today because your friend was diagnosed this year with HIV. You would like to have an HIV test also. At the health department, once you have a discussion with your provider about the importance of safe sex practices to prevent STIs and HIV, you are welcoming the change to your lifestyle.

Medical history: none

Medications/Allergies none

Family history: none

Social history: you do not smoke, no alcohol use, no drug use, sexually active with one partner now, male, and one partner prior to that for approximately 6 months. You cannot remember how many partners you have had exactly and your last STI screening was approx. one year ago.

Information for SP to respond to MI questions: (FEMALE)

- What are the patient’s life goals and values, especially with respect to the target behavior and overall health? Family? Work? Finances? You do not want to get HIV like your friend, and you didn’t think you could get it since you are heterosexual.
- How does continuation of the target behavior fit in or conflict with these goals? Unprotected sex could lead to STI’s like HIV.
- What are the good things about the target behavior? How does it benefit you? You love the spontaneity of sex, and you worry that using a condom would ruin the moment for you and your partner. You also worry that your partner will not want to continue having sex with you if you talk about using condoms. You don’t worry so much about SDI’s that can be treated easily like chlamydia, but you really don’t want HIV.
- What are the less good things about the target behavior? What are you afraid might happen if you keep engaging in the target behavior? For the past 12 years, pregnancy has not been a concern, but you worry about HIV and other STIs.
- What would be the advantages of making the behavior change? What are the best reasons to change? You would avoid STI’s like HIV.
- Explore ways to make the change (pros and cons of various methods, what has worked in the past): You could have a discussion with your partner about using condoms to prevent STIs and how to incorporate this into your sexual activity.
- Importance and confidence of changing the target behavior:
  - How important is it for you to change? 9/10
    - Why is that number that high and not lower? You don’t want HIV, and you now realize that you still need to worry about getting it even though you are heterosexual.
    - How could you move that number higher? Watching your friend go through treatment for his HIV will motivate you further, you believe.
  - How confident are you that you can change? 7/10
    - Why is that number that high and not lower? You have used condoms in the past.
    - What would make you more confident in your ability to change? Changing your beliefs/attitudes about condom use, incorporating it into foreplay, finding other ways to have spontaneity in sex.
- Ask for a decision: you will use condoms now.
- Set SMART (specific, meaningful, assessable, realistic, timed) goals. What are the first steps? You will buy condoms and have them available to use.

**Postoperative Patient Not Following Postoperative Instructions**

Presentation to the Student:

James Thompson is a 42-year-old male presenting to postoperative 2-week follow up appointment for a recent hernia repair (hysterectomy) surgery. He is a weightlifter and was lifting weights this past weekend with friends, as part of his exercise routine. The postoperative instructions specified no lifting over 15 pounds for 6 weeks duration.

Information for the SP:

You are a 42-year-old patient with recent surgery about 1 week ago (male = hernia repair, female = hysterectomy). You were told not to lift any objects over 15 pounds, but your friends invited you to work out and you lifted weights with them last weekend. Now, you are concerned about the surgical area during your postoperative visit. When the surgeon talks with you about the importance of the lifting restriction to protect your fresh surgical area, you are very motivated to comply with the postoperative instructions, as you would not like to have a second operation.

Medical history: none

Medications/Allergies: none

Family history: none

Social history: you quit smoking 10 years ago, you don’t drink alcohol or use drugs. You work as a sales representative and you can’t wait to return to this job once your wounds heal.

Information for SP to respond to MI questions:

- What are the patient’s life goals and values, especially with respect to the target behavior and overall health? Family? Work? Finances? You want to continue working at the job you love, heal well from your surgery, and be healthy. You really want to avoid a second surgery.
- How does continuation of the target behavior fit in or conflict with these goals? Once you are educated by the doctor about the dangers of lifting so early after your surgery, you realize that lifting weights is in conflict with your goals of healing well, being healthy, and returning to work.
- What are the good things about the target behavior? How does it benefit you? You think of yourself as strong and healthy, and you really like to work out with your friends because you are a social person.
- What are the less good things about the target behavior? What are you afraid might happen if you keep engaging in the target behavior? You don’t want to damage the surgical area and/or have a second surgery, and you don’t want to jeopardize your return to work or your general health.
- What would be the advantages of making the behavior change? What are the best reasons to change? Advantages are proper healing after your surgery and more assurance that you can avoid another surgery and return to work.
- Explore ways to make the change (pros and cons of various methods, what has worked in the past): You can learn to say no to lifting weights with friends in the next five weeks and you can find other ways to be social with friends.
- Importance and confidence of changing the target behavior:
  - How important is it for you to change? 9/10
    - Why is that number that high and not lower? You don’t want a second surgery and you want to return to work.
    - How could you move that number higher? You could learn more about complications of lifting.
  - How confident are you that you can change? 7/10
    - Why is that number that high and not lower? You really like to be social with your friends, but you also realize there are other ways to be social (like by going for a walk or out to dinner with friends).
    - What would make you more confident in your ability to change? You were able to quit smoking 10 years ago; you feel that you could find other ways to be social with your friends.
- Ask for a decision: you will not do any heavy lifting for the next 5 weeks.
- Set SMART (specific, meaningful, assessable, realistic, timed) goals. What are the first steps? You will tell your friends that you cannot lift heavy objects for the next 5 weeks.

**Pre-diabetic Who Needs to Lower Carbs**

Presentation to the student

Alexa D’Angelo is a 38-year-old healthy patient here for yearly follow-up in the office. Patient does not have any medical history and has expressed to you previously that she would like to avoid taking medications. Her mother has a history of type II insulin dependent diabetes and has had a below the knee amputation and requires dialysis three days per week. Patient’s vital signs are stable, but her blood work shows a fasting glucose of 170 (indicating “pre-diabetes”, which means she is at elevated risk for developing diabetes).

Information for the SP:

You are a patient returning to the clinic for a yearly follow up visit. You do not have any medical problems at this time, but your mother is a diabetic with multiple diabetic complications. Your blood sugar is high on your fasting lab work done prior to coming in. You do not have a special diet at this time, you eat a variety of foods, including candy occasionally, and you love to eat Italian food like pasta because you are Italian. After learning that you may have diabetes or prediabetes and further testing is necessary, you are open to making lifestyle changes, such as diet and exercise due to your mother’s complications resulting from diabetes.

PMH: none, no medications or allergies.

Family history: mother with type II insulin dependent diabetes with complications of amputation and kidney damage requiring dialysis three times per week.

Social history: you do not smoke, no alcohol intake, no drug use. You are a school teacher.   You don’t exercise regularly.  You have a partner you hope to marry soon.

Information for SP to respond to MI questions:

- What are the patient’s life goals and values, especially with respect to the target behavior and overall health?  Family? Work?  Finances?  You don’t want to end up like your mother, with her amputations and dialysis.  You want to get married to your partner and hopefully have kids soon (you’d like to be active with your kids).
- How does continuation of the target behavior fit in or conflict with these goals?  Continuing to eat pasta and candy, and not exercising, will raise your blood sugar and could get you diabetes, but you need the doctor to explain this to you.  You don’t understand the connection between your eating, lack of exercise, blood sugar, and mother’s health until the doctor explains it to you.
- What are the good things about the target behavior?  How does it benefit you?  You love to cook and you feel comfortable eating pasta because it reminds you of your homeland.  You eat candy with your school kids often and you feel your shared love of candy makes you popular with your kids.  You don’t exercise because it is so hard to find the time.
- What are the less good things about the target behavior?  What are you afraid might happen if you keep engaging in the target behavior?  You don’t want to gain weight and you don’t want to develop diabetes or have complications of diabetes.
- What would be the advantages of making the behavior change?  What are the best reasons to change?  Not ending up like your mother.
- Explore ways to make the change (pros and cons of various methods, what has worked in the past): You could get sugar-free candy, you could explore other cuisines with fewer carbohydrates, you could restrict your carbs, you could get your partner on board with helping you change, you could pair up with your mother in eating a diabetic diet.  To begin exercising you could identify another teacher as an exercise buddy and walk the track at lunch, or you could organize an exercise club with the kids and work out with them (before or after school, or at lunch).
- Importance and confidence of changing the target behavior:
  - How important is it for you to change? 4/10 (low because you really love pasta and candy, and you don’t understand the connection with diabetes and your mom’s complications)
    - Why is that number that high and not lower? You want to be healthy, you want to get married and have kids
    - How could you move that number higher? Understanding the connections between your eating and diabetes.  Perhaps making some lifestyle changes with your mother (you would do it for your mother even if you struggled to do it for yourself).
  - How confident are you that you can change? 5/10
    - Why is that number that high and not lower? Because you really love your mother, and you really don’t want the complications she has had from diabetes.
- What would make you more confident in your ability to change? Understanding the connections between eating/exercise and diabetes, understanding the complications that can arise from uncontrolled sugars, exploring diabetic diets, working on your dietary goals with your mother.
- Ask for a decision: You will consider changing your diet and starting to exercise.
- Set SMART (specific, meaningful, assessable, realistic, timed) goals.  What are the first steps? You will talk with your mom about making dietary changes together, you will buy and try sugar free candy, you will learn about healthy eating for diabetics, and you will ask a colleague at work to walk with you at lunch.
