# Supplementary material for: Motivational Interviewing Training: A Case-Based Curriculum for Preclinical Medical Students
Source: MedEdPORTAL. 2021 Feb 12;17:11104. doi: 10.15766/mep_2374-8265.11104 (PMC7880250; doi:10.15766/mep_2374-8265.11104)
Supplement: Supplementary file 1 — Presurvey.docxMI Presentation.pptxMI Demonstration Script.docxTransparent Outline for MI Activity.docxMICA Evaluation Tool.docPractice Cases.docxMI Summary Sheet.docxEvaluated Cases.docxOARS Tracking Sheet.docChange Talk Tracking Sheet.docMI Evaluated Session Sample Schedule.xlsxActing Patient Experience Scale.docxPostsurvey.docxFacilitator Guide.docx [file mep_2374-8265.11104-s001.zip › L. Acting Patient Experience Scale.docx]

**Acting Patient Experience (APEx) Scale:**

Your responses to these scaled feedback questions below are designed to support the medical student’s self-reflection while they are trying to apply motivational conversation spirit, skills and strategies to an actual conversation about change.

**|-----------------------|----------------------|---------------------|-----------------------|**

**1 2 3 4 5**

**Not at all Moderately Very much**

| **ITEM** | **RATING (1-5)** |
| --- | --- |
| The student listened and understood what mattered to me. |  |
| The student and I decided together what was important to talk about. |  |
| The student asked me about my reasons and confidence for making changes. |  |
| The student understood and affirmed my strengths. |  |
| The student and I worked together for a plan that suits my goals and preferences. |  |
| The student asked permission before offering me information or suggestions. |  |

For further information about this scale or incorporating into mirrored feedback options, please contact Ali Hall:

ali@alihalltraining.com
