# Supplementary material for: Motivational Interviewing Training: A Case-Based Curriculum for Preclinical Medical Students
Source: MedEdPORTAL. 2021 Feb 12;17:11104. doi: 10.15766/mep_2374-8265.11104 (PMC7880250; doi:10.15766/mep_2374-8265.11104)
Supplement: Supplementary file 1 — Presurvey.docxMI Presentation.pptxMI Demonstration Script.docxTransparent Outline for MI Activity.docxMICA Evaluation Tool.docPractice Cases.docxMI Summary Sheet.docxEvaluated Cases.docxOARS Tracking Sheet.docChange Talk Tracking Sheet.docMI Evaluated Session Sample Schedule.xlsxActing Patient Experience Scale.docxPostsurvey.docxFacilitator Guide.docx [file mep_2374-8265.11104-s001.zip › N. Facilitator Guide.docx]

**Facilitator Guide**

**Motivational Interviewing Training: A Case-Based Curriculum for Pre-Clinical Medical Students**

Background

- With the rise of chronic medical problems involving lifestyle behaviors and the benefits of patient involvement in preventative care, medical students need to learn how to help patients change health risk behaviors and improve patient involvement in order to improve health outcomes. Motivational interviewing is a patient-centered therapeutic approach that is effective in the treatment of lifestyle behaviors and diseases.
- This training package includes a 2-hour didactic session, a 3-hour facilitated practice session using role-plays, and a facilitated 3-hour evaluated session using Standardized Patients (SPs).
- Objectives - By the end of this activity, learners will be able to:
  - Explain why physicians should be proficient in helping patients change lifestyle behaviors
  - Describe the stages of change, and how Motivational Interviewing (MI) can help move patients through these stages
  - Summarize the research base for use of MI in healthcare settings
  - Describe the spirit, general principles, tools, and core skills of MI
  - Apply MI skills to a wide variety of target lifestyle behaviors

Recommended PowerPoint Presentation Timing (2 hours overall)

- 10 minutes: Pre-survey (Appendix A)
- 90 minutes: MI skills with embedded student practice and debrief (Appendix B slides 3-36)
- 10 minutes: MI Demonstration (Appendix B slides 37-38, Appendix C)
- 10 minutes: Prepare for practice and evaluated sessions (Appendix B slides 39-48)

Plan for Practice Session

- The practice session is meant to allow the students the opportunity to practice using the MI skills they have learned in lecture and to get feedback from facilitators on the extent to which their skills match MI philosophy and strategy.
- Materials needed in packet:
  - Names of students in the small group (with class photos, if possible)
  - Two sets of copies of the practice cases (Appendix F) – one for the facilitator to keep and one set to pass out to students.
  - MI Summary Sheet (Appendix G, students are allowed to use this during the session)
- After the facilitator and students introduce themselves to one another, the facilitator should hand out one case to each student. The student who receives the case is the patient in that case.
- Ask students to pair up with one another. The student holding the case is the patient and the other student is the doctor in the interaction.
- The facilitator should read out load for the whole group the first paragraph of the case titled “Presentation to Student.” Student pairs should act out the case one by one (in front of the whole group), and the student acting as the doctor should get feedback from the facilitator, then apply the corrective feedback (once you have offered feedback, we hope that you will allow students an opportunity to try the interaction again).
- After the first student is finished acting the part of the doctor, trade such that the other partner is now the doctor and proceed with that case. Repeat with the remaining pairs, until each student has had an opportunity to role play as a doctor.
- In a 3-hour session with 8 students, each case should get about 20 minutes (including feedback).
- Collect the cases at the end (to preserve the ability to use them again the following year).
- Note: the practice session has no evaluative component associated with it.

Plan for Evaluated Session

- The evaluated session is intended to be a summative assessment of students’ MI skills, so facilitators will fill out the MICA Evaluation Tool (Appendix E) for each student. The order of students being evaluated should be predetermined and based on alphabetical order of student names, and students should be pre-assigned to a case, as indicated by the sample Evaluated Session Schedule (Appendix K). In addition, the session offers students the chance to give one another feedback using the Change Talk and OARS Tracking Sheets. Student observers should be pre-assigned to fill out either OARS or Change Talk Observer Tracking Sheets for one another (Appendices I and J, respectively), such that for each student playing the role of the physician, there is one other student filling out the OARS Observer Tracking Sheet and another student filling out the Change Talk Observer Tracking Sheet. These tracking sheets are meant to engage observers and offer them a framework in which to offer feedback to their peers.
- Materials needed in packet:
  - Session Schedule Grid (Appendix K), with students preassigned to cases.
  - Names of students in the small group (with class photos, if possible).
  - MI Summary Sheet (Appendix G)
  - MICA Evaluation Tool (Appendix E, one copy for each student)
  - Change Talk Tracking Sheet (Appendix J, one copy for each student)
  - OARS Tracking Sheet (Appendix I, one copy for each student)
  - Evaluated Cases (Appendix H, one set for facilitator)
- Students and the facilitator stay in the same room, and the SPs rotate from room to room, acting out their case, according to the Session Schedule Grid (Appendix K). Each student will take turns interacting with one SP, and when it is a student’s turn, they should move to the head of the table. The facilitator can sit closest to the head of the table, so they can observe the student well. The SP can be invited to sit opposite the facilitator and at the other side of the table closest to the head.
- Flow/timing of the evaluated session:
  - Each case has 20 minutes. The timing is tight so the facilitator MUST keep the timing on track. If one room is running late, it will cause a ripple effect to make all the other rooms late, since they share SPs. The facilitator may want to assign timing to another student in the room, so that the facilitator can focus on the interaction and evaluation.
  - Read the “Presentation to Student” on the Evaluated Case assigned prior to the SP walking into the room so that the student is familiar with the presenting issue, and ensure that the student understands what the target behavior is. The point of these cases is not for the student to figure out the diagnosis, but to demonstrate their MI skills.
  - Of the 20 minutes, the first 5-10 minutes should be spent with the student interviewing the SP, uninterrupted.
  - The next 5-10 minutes are for feedback from the facilitator, SP, and other students using the OARS and Change Talk Tracking Sheets, as well as the student re-doing any parts of the case needed given your corrective feedback.
  - 15 minutes into the case (with 5 minutes left), the SP’s will leave to fill out their evaluations (Acting Patient Experiences Scale, Appendix L) and prepare to enter the next room, and you can use the last 5 minutes for any additional feedback to the student along with a summary. SP evaluations should be collected by the SP Coordinator and given to the course director. Course directors may or may not choose to incorporate these evaluations into the student grades.
- Once all students have had a turn with the SP, the facilitator should share the Post-Survey (Appendix M) with the students and ask them to complete it prior to leaving. Surveys can be completed using a QR code, a link, or via paper copies.
- Facilitators should be sure to complete all student evaluations using the MICA Evaluation Tool (Appendix E) and turn these in to the course directors, along with the OARS and Change Talk Tracking Sheets (Appendices I and J) collected from the students.

Alternative Modes of Delivery

- Cases are interchangeable between practice and evaluated sessions.
- Course directors can skip the evaluated session and only do practice cases using role playing. This would be a good choice if SPs are not available.
- Course directors can choose to incorporate the cases into didactics, using role playing with students, if they are limited on time or availability of facilitators or SPs.
- Rather than going through the cases in a small group format, with all students observing one another’s cases, course directors could choose to have students come in two at a time for the practice cases (in order to role play) and one at a time in order to have the evaluated SP interaction. If this alternative is selected, students may lose out on opportunities to learn from watching one another’s cases and hearing the facilitator offer feedback to other students.

Facilitator Requirements and Role

- One facilitator is needed per small group.
- The facilitator must be familiar with MI, with knowledge of MI principles, skills, and techniques, and should have experience using MI with patients in clinical settings. Facilitators can be physicians or other health care professionals, such as Physician Assistants, Nurse Practitioners, Nurses, or mental health professionals.
- Facilitators should be able to provide feedback on students’ use of MI principles, skills, and techniques, including corrective feedback. Facilitators can model use of MI in role plays and with SPs, and then encourage students to practice the skill modeled.
- Facilitators should be able to encourage all students to participate, such as in offering one another feedback on MI performance.
- Facilitators should read all MI materials in this package prior to the group sessions.

Training/Issues for Standardized Patients

- Gender and age can be changed in most cases based on the SP pool available, except for in cases where the gender and/or age are specific to the presenting problem, as in the case of the pregnant patient who is not compliant with prenatal care.
- SPs can attend the PowerPoint presentation on MI included in this package (Appendix A) along with the students, so that they can understand the spirit of MI and the techniques the students will be using with them. You can also record this presentation and make it available for SPs to watch on their own. Familiarity with MI will allow SPs to give feedback to students using MI language, such as “I felt you supported my autonomy as a patient,” “I noticed that you used open ended questions to elicit change talk,” or “you offered a lot of advice without asking me if that would be alright or asking me afterwards where that advice left me in terms of my confidence in changing.” SPs should also use the Acting Patient Experiences Scale (Appendix L) to guide their feedback.
- SPs should reward students with appropriate information when asked open-ended questions or other questions/techniques that are consistent with the spirit of motivational interviewing (i.e., asking about goals, pros and cons of behavior, asking SPs what they are willing to do, what they think will work, previous successes, etc.)
- SPs should become more resistant (i.e., more difficult to work with, less open to change, “yeah, but …”) when students engage in behavior that is not consistent with motivational interviewing, such as giving advice without asking permission first.
- After the encounter, SPs should use the Acting Patient Experiences Scale to guide their feedback to students. Specifically, they should offer feedback about the extent to which they felt the student elicited their thoughts and views about the importance of change and ways to change, health goals and plans for change, and the extent to which the SP felt understood, respected, and like an equal teammate with the provider.

Tips for Success

- Course Directors should work closely with the SP coordinator to ensure that SPs are trained properly, adhere to the case scripts, and give feedback with fidelity to MI.
- Course Directors and their assistants should organize the packets with cases and evaluation tools pre-assigned to students, and names pre-populated when possible, so that the facilitator knows who is evaluating who on which case. Materials should be organized in order of the cases, so the facilitator doesn’t need to waste time looking through paperwork.
- Facilitators should read all materials prior to the sessions, as the timing goes very quickly and requires familiarity with the materials in order to stay on track with time.
- Facilitators *must* stay on track with time. If possible, overhead announcements can be used to let them know how much time is left in each session. Alternatively, students can be assigned to help facilitators keep track of time.
- Course directors may want to meet with facilitators prior to the practice and especially the evaluated session, in order to go over materials, timing, and evaluations. Facilitators can be asked to come one hour early to practice and evaluated sessions for this orientation.
- Facilitators should create a positive learning climate for students, such that they have a good experience with MI and gain confidence in their ability to use the skill sets. They should encourage students to use the MI Summary Sheet (Appendix G) if needed.
