# Supplementary material for: Motivational Interviewing Training: A Case-Based Curriculum for Preclinical Medical Students
Source: MedEdPORTAL. 2021 Feb 12;17:11104. doi: 10.15766/mep_2374-8265.11104 (PMC7880250; doi:10.15766/mep_2374-8265.11104)
Supplement: Supplementary file 1 — Presurvey.docxMI Presentation.pptxMI Demonstration Script.docxTransparent Outline for MI Activity.docxMICA Evaluation Tool.docPractice Cases.docxMI Summary Sheet.docxEvaluated Cases.docxOARS Tracking Sheet.docChange Talk Tracking Sheet.docMI Evaluated Session Sample Schedule.xlsxActing Patient Experience Scale.docxPostsurvey.docxFacilitator Guide.docx [file mep_2374-8265.11104-s001.zip › _Educational_Summary_Report_11104.pdf]

MedEdPORTAL® The AAMC Journal of Teaching and Learning Resources

Original Publication Open Access

Motivational Interviewing Training: A Case-Based Curriculum for Preclinical Medical Students

N. Nicole Jacobs, PhD*, Lisa Calvo, MD, Aaron Dieringer, MD, MPH, Ali Hall, JD, Reka Danko, MD

*Corresponding Author: nnjacobs@med.unr.edu

Abstract

Introduction: With the rise of chronic medical problems involving lifestyle behaviors and the benefits of patient involvement in preventative care, medical students need to learn how to help patients change health risk behaviors and improve patient involvement in order to improve health outcomes. Motivational interviewing (MI) is a patient-centered therapeutic approach that is effective in the treatment of lifestyle behaviors and diseases. **Methods:** This 2-hour didactic training session, along with a 3-hour case-based practice session involving role-plays and a 3-hour evaluated session utilizing standardized patients, was delivered to 68 preclinical medical students. Knowledge, attitudes, and self-efficacy were evaluated via pre- and posttraining surveys, and satisfaction with the training was assessed upon completion. **Results:** Students who completed both pre- and postsurveys ($n = 48$) showed a statistically significant improvement in knowledge of MI ($t = -29.73$, $df = 47$, $p < .001$), attitudes regarding implementing MI in health care settings ($t = -3.04$, $df = 47$, $p < .005$), and self-efficacy ($t = -10.699$, $df = 47$, $p < .001$) in talking with patients about behavior change. Students were also highly satisfied with the MI training package (M of 4.4, $SD = 0.6$, out of 5.0). **Discussion:** A training package to teach preclinical medical students about MI was effective in helping students learn the knowledge and skills necessary to deliver MI in a broad range of clinical cases.

Keywords
Motivational Interviewing, Communication Skills, Preventive Medicine

Educational Objectives

By the end of this activity, learners will be able to:

1. Explain why physicians should be proficient in helping patients change lifestyle behaviors.
2. Describe the stages of behavior change, and how motivational interviewing (MI) can help move patients through these stages.
3. Summarize the research base for use of MI in health care settings.
4. Describe the spirit, general principles, tools, and core skills of MI.
5. Apply MI skills to a wide variety of target lifestyle behaviors.

Citation:
Jacobs NN, Calvo L, Dieringer A, Hall A, Danko R. Motivational interviewing training: a case-based curriculum for preclinical medical students. *MedEdPORTAL*. 2021;17:11104.
https://doi.org/10.15766/mep_2374-8265.11104

Introduction

Chronic medical problems, such as diabetes, hypertension, and obesity, are widespread and on the rise. Prevention and treatment of these chronic medical problems are more successful with patient engagement, adherence to treatment recommendations, and lifestyle changes, such as healthier diet and exercise. Four modifiable health risk behaviors, lack of physical activity, poor nutrition, tobacco use, and excessive alcohol consumption, have been identified as causing or exacerbating much of the morbidity and mortality related to chronic disease in the developed world.[1] Other behavioral factors, such as lack of treatment adherence, have also been identified as a major contributor to health problems, with up to 50% of patients with chronic disorders adhering poorly to treatment or medication regiments.[2]

A modern textbook on behavioral sciences for medical students stated that the expanding role of physicians is not just to diagnose and treat chronic illnesses, but to partner with patients in helping them make lifestyle changes to improve their health.[3] The authors discussed the importance of doctors helping patients to change their beliefs, behaviors, and sociocultural practices.

Copyright © 2021 Jacobs et al. This is an open-access publication distributed under the terms of the Creative Commons Attribution license.

Unfortunately, with a lack of training and reflective practice in this skill set, physicians-in-training often fail to take advantage of opportunities to help patients change.

Motivational interviewing (MI) is an evidence-based, patient-centered, collaborative conversation style and therapeutic approach designed to strengthen a patient's own motivations and commitment to change. MI was originally developed in the field of alcohol and substance use[4,5] but was quickly adopted by health professions,[6] where it has come to be known as an intervention strategy that is useful in the treatment of lifestyle behaviors and diseases. MI has developed a strong evidence base for helping patients to modify lifestyle behaviors such as diet, exercise, substance use, and treatment adherence, and in improving patient outcomes with such chronic health problems as diabetes, cardiovascular disease, hypertension, asthma, and HIV.[7-11] Because of these beneficial outcomes, and because MI has been shown to increase patient satisfaction,[12] some professional organizations such as the American College of Obstetricians and Gynecologists have encouraged the use of MI to elicit patient behavioral change and have recommended that MI principles be incorporated into training of medical students and physicians.[13]

Many health professions programs have successfully taught MI to health care professionals and learners.[14-24] Meta-analyses of MI training for health care practitioners[25] found generally positive outcomes for such programs. One meta-analysis[10] looking at the impact of physicians using MI showed an effect in 83% of studies, which was even higher than the rate of effectiveness of psychologists (79%). This meta-analysis also showed an effect in 64% of studies where the MI encounter with a patient was less than 20 minutes, indicating feasibility of use of MI in busy clinical settings.

However, as MI is a complex skill set, recommendations for teaching have been set forth by its developers. Previous research[26] has recommended eight stages for learning MI, and this framework was used to develop the training described in this report: (1) learning the spirit of MI, including a therapeutic style that emphasizes collaboration, evocation, and patient autonomy; (2) developing patient-centered therapeutic skills such as the ability to ask open-ended questions, affirm the patient's experiences, and reflect and summarize the patient's responses; (3) recognizing and reinforcing patient talk related to change (i.e., desire, ability, reasons, need, or commitment to change); (4) eliciting and strengthening change talk in patients; (5) rolling with patient resistance to change; (6) helping the patient to develop a change plan; (7) consolidating the patient's commitment to the change plan; and (8) switching between MI and other therapeutic styles. All training programs reviewed in the above-referenced meta-analysis included stages one through seven, but not eight.[25]

Health care professionals need to acquire knowledge about how to help patients make lifestyle changes and confidence in their ability to do so, as well as an attitude that implementing these skills is possible in primary care settings. The training described in this study was designed to introduce preclinical medical students to MI, as well as offer them formative practice cases in which they can apply their knowledge and skills via role-plays. It also included a summative evaluation component to be used with standardized patients (SPs) for course directors to assess students' MI skills. The cases targeted a broad variety of lifestyle behaviors typically seen in clinical settings, such as lack of adherence to treatment regimens or medication, excessive alcohol use, smoking, poor diet, lack of exercise, and unsafe behaviors (i.e., not wearing seat belts, unprotected sex), so that medical students new to learning about MI can see how it can be applied to a wide range of problematic lifestyle behaviors.

A literature review of *MedEdPORTAL* revealed several curricula to teach patient-centered MI skills to medical learners, and these curricula varied in intended audience, focus, intensity, and whether they were case-based or used SPs. Most curricula were intended for residents[14,16,23,27] or primary care providers,[18] but two were targeted toward first- or second-year medical students.[15,20] Most of the educational packages had a limited health behavior focus, such as on smoking cessation,[16,27] substance use,[14,23] weight loss,[15,20] or issues specific to female military veterans.[18] One training was intended for medical advisors to use with medical learners in order to increase their motivation to learn so they could enhance their career development.[21] The length and intensity of trainings varied widely, from a 1-hour refresher course[23] to a 4-week (16 hour) curriculum.[14] Only some of the MI curricula included cases to role-play[15] or to use with SPs.[18,20,27] If the curricula did include cases, there were no more than four included in the publication. Our curriculum fills a gap in the *MedEdPORTAL* literature in that it was intended for preclinical medical students who were new to learning about MI and utilized seven of eight components in the MI training framework suggested by previous research.[26] Because research[6] has emphasized that the complex clinical skill set in MI takes time to master, our didactic session was targeted to 2 hours. The learning was augmented by two additional 3-hour case-based sessions focused on experiential learning using role-plays with practice cases (first 3-hour session) and skills practice

Copyright © 2021 Jacobs et al. This is an open-access publication distributed under the terms of the Creative Commons Attribution license.

with SPs in evaluated cases (second 3-hour session), as it has been noted that modern learning theories stress the value of reflective practice and experiential learning.[28] The cases were intended to have a wide medical focus in order to demonstrate how broadly MI can be used in health care settings. This unique combination of introductory level, but thorough, MI curricular materials combined with a case-based formative practice session and skills-based SP evaluated (summative) session with a broad medical focus for preclinical medical students fills a gap in the literature and can be easily adopted into a clinical skills course.

Methods

We developed a 2-hour didactic session to introduce MI skills to first-year medical students in a large-group setting at the University of Nevada, Reno School of Medicine (UNR Med) in the context of the Practice of Medicine (POM) block, a clinical skills course. We followed this up with a 3-hour small-group session where the students could apply MI skills using role-plays involving practice cases, and receive feedback on their use of MI from clinical facilitators. Finally, students were evaluated on their MI skills in another 3-hour small-group setting using SPs who acted out cases. Students were asked to complete a presurvey immediately prior to the first large-group session and a postsurvey immediately following the final evaluated small-group session.

Training Session

Sixty-eight first-year medical students attended a required 2-hour educational session in a large classroom setting to learn about MI in health care settings. The educational session began with an anonymous presurvey (Appendix A) to assess learners' knowledge, self-efficacy, and attitudes about implementing MI in a medical setting. Consistent with previous recommendations[26] for MI training, the presentation (Appendix B) included content on stages of behavior change, general principles of MI, MI strategies, core MI skills, eliciting and reinforcing change talk, and helping the patient develop a change plan. Opportunities for think-pair-share activities with mini case examples were embedded throughout the training. A live demonstration of MI (Appendix C), with the course directors role-playing the part of a patient and a physician, was then completed in front of the class. The session ended with an explanation of the practice and evaluated small-group sessions to be conducted over the subsequent 2 weeks, including a transparent activity outline document that explained the rationale for the MI sessions (Appendix D) and an explanation of the grading tool and its components (Appendix E). The recommended session order and timeline were as follows:

- 10 minutes: presurvey (Appendix A).
- 90 minutes: MI skills with embedded student practice and debrief (Appendix B, slides 3-36).
- 10 minutes: MI demonstration (Appendix C and Appendix B, slides 37-38).
- 10 minutes: prepare for practice and evaluated sessions (Appendix B, slides 39-48).

Practice (Formative) Small-Group Session

Since the POM block meets once a week, the week after the training session students met again, this time in small-group format (8 students per group, 9 groups total) in standard small-group rooms (not patient rooms) for 3 hours total. The small-group practice sessions were facilitated by a clinical attending familiar with MI, such as a primary care provider or mental health specialist at UNR Med. Eight practice cases (Appendix F) were handed out to students such that each student received one case. Students were asked to play the part of the patient in the case they received, and they paired up with another student who was asked to play the part of the physician. Students were asked not to show one another their cases. Students took turns acting out each case in front of the whole group, such that each case took about 20 minutes.

Students were then provided with the MI summary sheet (Appendix G) and allowed to use this sheet for both the practice and evaluated sessions. Students were encouraged to offer feedback to one another on how they used MI skills well and how they could improve. Facilitators also gave feedback to the students role-playing the physician, focusing on the quality of how students applied the MI spirit, general principles, core skills, and tools of MI.

The practice session was meant to allow students an opportunity to implement MI skills in cases involving the need for behavior change in the context of a clinical visit, and for students to get feedback from one another and from a facilitator. In an effort to lower anxiety about the evaluated session, the practice session was also meant to offer students a chance to work with cases that were written exactly like their evaluated cases in the upcoming session. In fact, the practice and evaluated cases offered in this training package can be used interchangeably. Students were asked to practice in a group setting rather than individually so that they could learn from one another's cases and from the feedback that the facilitator provided not only to them, but to their classmates. However, this activity could be modified such that students only participate in a 40-minute block as dyads, with each student role-playing a case and receiving feedback from a facilitator for 20 minutes each.

Copyright © 2021 Jacobs et al. This is an open-access publication distributed under the terms of the Creative Commons Attribution license.

Evaluated (Summative) Small-Group Session

The week following the practice session, students participated in the evaluated session with SPs. Appendix H contained the evaluated cases. The evaluated sessions occurred using the same small groups and rooms as were used for the practice sessions, with the same facilitators. The order of students being evaluated was predetermined and based on alphabetical order of student names, and students were preassigned to a case. Student observers were also preassigned to fill out either open-ended questions, affirmations, reflections, summaries (OARS), or change talk observer tracking sheets for one another (Appendices I and J, respectively), such that for each student playing the role of the physician, there was one other student filling out the OARS observer tracking sheet and another student filling out the change talk observer tracking sheet. These tracking sheets were meant to engage observers and offer them a framework in which to offer feedback to their peers. Students and the facilitator remained in the same small group room but SPs moved from room to room portraying the same case (see Appendix K for sample schedule). Each student took turns playing the part of the physician with an SP and this occurred in front of the entire small group, so that students could learn from one another's cases and feedback received. However, this activity could be modified such that students come in, one by one, for their case only. The case introduction was read to the student prior to the SP entering the room, to orient them to the details of the case. Each case took 20 minutes, broken down as follows:

- 5-10 minutes: SP acted out the case and student demonstrated MI skills.
- 5-10 minutes: SP provided feedback to the student (guided by the acting patient experience scale, Appendix L), other students offered feedback (guided by the OARS and change talk observer tracking sheets, Appendices I and J), and the facilitator offered feedback (guided by the MI competency assessment evaluation tool, Appendix E) and allowed the student to try again with the SP, implementing feedback.
- Last 5 minutes: SP left the room and prepared to enter another student room while feedback continued for the student playing the role of the physician.

At the end of the evaluated session, students were asked to take a postsurvey (Appendix M). This postsurvey contained the same questions as the presurvey to assess knowledge, self-efficacy, and attitudes about using MI, but also contained questions to assess learners' satisfaction with the MI training package.

Scenario Development and SP Training

Practice (Appendix F) and evaluated (Appendix H) case scenarios were written and edited by all authors, including one clinical psychologist with expertise in MI, (N. Nicole Jacobs, PhD), one consultant who is a member of the Motivational Interviewing Network of Trainers (Ali Hall, JD), two internists (Lisa Calvo, MD and Reka Danko, MD), and one family medicine physician (Aaron Dieringer, MD, MPH). All scenarios were based upon cases seen by the authors and were designed to cover a wide range of clinical scenarios in which patient behavioral/lifestyle change was necessary to improve their medical health. Cases involved a broad range of problems such as missing clinic appointments, lack of treatment/medication adherence, not wearing a seat belt, smoking, excessive drinking, lack of exercise, poor diet, lack of prenatal care, and unprotected sex. Standard medical information was not provided in the cases because the focus of all encounters was on behavioral issues. All cases started with a paragraph titled "Presentation to Student," designed to be read to the student prior to the case, in order to orient students to the behavioral problem needing to be addressed during the encounter. Cases also included information for the SP, including basic medical information and answers to MI-based questions (such as those found in the MI summary sheet) the students would likely ask. SPs were trained for these cases by listening to the same presentation on MI that the students received (Appendix B), in order to become familiar with MI philosophy, spirit, techniques, and skills. SPs also worked with our SP educator to rehearse the cases and ensure fidelity to the scripts. Training took 2 hours for experienced SPs and 3 hours for newer SPs. It is important to note that age and gender of SPs written into the cases can be flexible to meet the needs of available SPs, unless these factors are central to the presentation or otherwise designated in the cases.

Grading and Feedback

Four evaluation tools were implemented, each filled out by the different stakeholders involved in this activity. First, facilitators used the MI competency assessment tool (Appendix E, developed by Ali Hall, JD, and available in the public domain on the Motivational Interviewing Network of Trainers website)[29] to evaluate students. This tool provided behaviorally anchored scales in each of five domains: supporting autonomy and activation, guiding, expressing empathy, partnering, and evoking. There were five behaviorally anchored scoring indicators within each of the five scales, for a maximum of 25 points. Space was added for facilitators to comment on students' strengths and areas of improvement. This scale was used to grade students on the activity and was also intended to guide

Copyright © 2021 Jacobs et al. This is an open-access publication distributed under the terms of the Creative Commons Attribution license.

facilitator feedback to students. Second, students evaluated one another using the OARS and change talk observer tracking worksheets (Appendices I and J, available in the public domain on the Motivational Interviewing Network of Trainers website[30]) which involved tally marks for each behavior observed as well as additional comments for use of MI skills. The OARS sheet allowed observers to track use of open-ended questions, affirmations, reflections, and summaries. The change talk sheet allowed tracking of different types of change talk. Third, SPs used the acting patient experience scale (Appendix L, developed by Ali Hall, JD) to evaluate students using a 5-point Likert-type scale addressing various domains thought to be important in a patient's experience of engaging in MI. Finally, students themselves were asked to provide feedback to course directors on their experience with the MI modules and satisfaction with their training. The satisfaction questions were embedded into the postsurvey (Appendix M) that students took immediately following the evaluated session.

Facilitator Guide

A facilitator guide (Appendix N) was provided to course directors and all small-group facilitators. This guide contained background information on MI, recommended timing of the PowerPoint, instructions for the practice and evaluated MI sessions, alternative methods of delivering the content, requirements and roles of the facilitator, notes on training of SPs, and tips for ensuring success.

Evaluation

The study was approved by the Institutional Review Board at the University of Nevada, Reno as exempt. The impact of the MI training package was evaluated through use of pre- and posttests given to students via Qualtrics surveys. Both before and after the training, participants were given a questionnaire that was designed to measure their knowledge, self-efficacy, and attitudes regarding implementation of MI (Appendices A and M). A questionnaire designed to measure satisfaction was also given to participants following the training (Appendix M). The surveys (pre and post) were developed by the authors of this study and satisfaction questions were piloted with a previous cohort of medical students. We did not collect demographic information for this study, as we wanted to ensure student anonymity. However, students did create an individual nonidentifiable ID so that pre- and postsurveys could be matched and within-subject analyses could be conducted.

Only students who completed both pre- and posttests were included in the within-subjects analyses. Knowledge was assessed using six questions (questions 1-6). Self-efficacy was measured as the average response of six questions (questions 7-12) with original responses measured on a 5-point Likert scale (5 = *strongly agree*, 4 = *agree*, 3 = *neutral*, 2 = *disagree*, 1 = *strongly disagree*). Attitude was measured using three questions with the same 5-point Likert scale (questions 13-15). Satisfaction was measured using eight questions (Appendix M, questions 16-23) with the same 5-point Likert scale. Summary statistics for binary endpoints (knowledge) were presented as percentages, while Likert scale data were presented as $M \pm SD$. Changes from pre- to posttraining in knowledge, self-efficacy, and attitude were tested using a paired t test. We used R Studio version 1.2.5033 (R Foundation for Statistical Computing) for statistical analyses. In all cases, significance was assessed at $p = .05$.

Results

Of the 68 first-year students enrolled in the course, 48 completed both the pre- and posttest. Prior to the training, the mean knowledge test score was 4 ($SD = 1.4$) out of 11 (36%; Figure). Following the training, participants' knowledge test scores significantly increased by 59% ($M = 10.4$, $SD = 1.1$) to an average of 95% ($t = -29.73$, $df = 47$, $p < .001$; Figure). Before training, the mean self-efficacy score was 3.3, $SD = 0.6$ (Figure). Following the training, participants' self-efficacy scores significantly increased by 29% to $M = 4.2$, $SD = 0.5$ ($t = -10.699$, $df = 47$, $p < .001$; Figure) and above the threshold of agreement, or the point on the Likert scale where the agreement statement start (4). Prior to training, the mean attitude score was 4.1, $SD = 0.5$ (Figure). Following the training, participants' attitude scores significantly increased by 8% ($M = 4.4$, $SD = 0.5$; $t = -3.04$, $df = 47$, $p < .005$; Figure). Finally, following the training, the mean score of eight questions assessing satisfaction was 4.4 ($SD = 0.6$).

Discussion

A 2-hour training session coupled with a 3-hour case-based role-play practice session and a 3-hour evaluated session with SPs was developed to teach and assess MI knowledge and skills for preclinical medical students. The scope of behavioral problems to which MI was applied was broad. This type of training for premedical students, with such a broad focus, fills an important gap in the literature and allows other training programs to utilize an introductory-level educational package to teach and assess the increasingly important skill of working with patients on behavioral change in order to improve health outcomes. Surveys comparing pretraining and posttraining data in premedical students showed a statistically significant improvement in participants' knowledge of MI, attitudes about use of MI in health care settings, and self-efficacy in working

Copyright © 2021 Jacobs et al. This is an open-access publication distributed under the terms of the Creative Commons Attribution license.

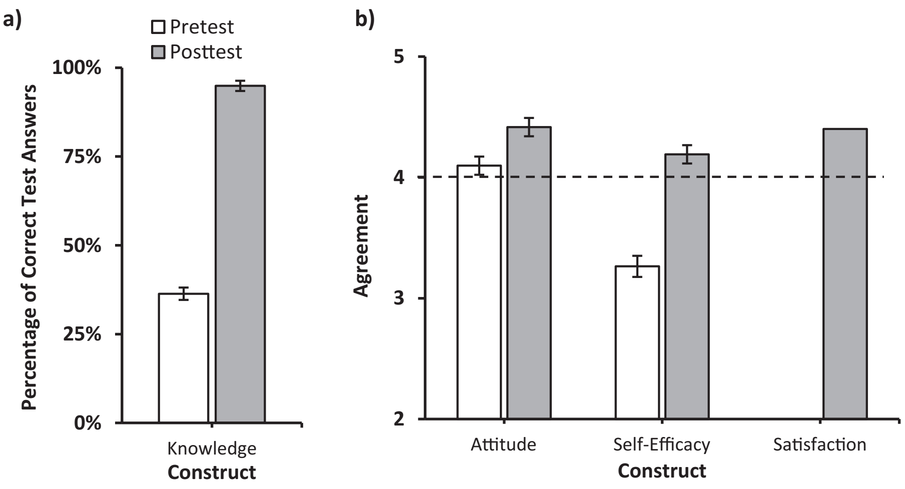

Figure. Mean values and standard error bars for overall attitude, self-efficacy, satisfaction, and knowledge scores, from first-year medical students ($n = 48$) before (pretest) and after (posttest) the motivational interview training. a) Knowledge was scored based on the percentage of correct test answers out of a possible total of 11. b) Attitude, self-efficacy, and satisfaction were scored based on the average rating of each question on a 5-point scale (1 = *strongly disagree*, 5 = *strongly agree*). The dotted line represents the threshold of agreement, the point at which agreement statements start (4).

with patients on lifestyle changes, as well as very high levels of satisfaction with the training program.

Despite promising results, our study had several limitations. First, it was conducted at one school, so our outcomes may not generalize to other teaching institutions. Second, it is unknown whether the gains in knowledge and skills seen in our training session will translate into real world clinical experiences with patients, and whether these will result in improved health outcomes for patients. Third, since we had no follow-up assessments, it is not known whether gains seen with the posttraining assessment continued over time. Finally, although this training package was meant to be of benefit to other health professions learners, such as nurses and physician assistant students, our data come from medical students only thus far and do not allow for these generalizations.

Although our study had several limitations, there were many strengths of the approach we utilized. First, the PowerPoint presentation with facilitation notes made it easy for a teacher with limited MI background to implement the training. The training included a script for the teacher to demonstrate MI skills to the class. Second, the package included a summary handout for medical students, which they could use as they advance through their training. Third, the cases were broad in their inclusion of types of scenarios commonly encountered in clinical practice and demonstrated the wide applicability of MI to a variety of health problems. The cases were also flexible such that programs that do not have SPs can utilize the cases in role-play format. The eight practice and 12 evaluated cases can be used interchangeably, for a total of 20 cases. Fourth, the training included a practice session, which allowed for students to receive guided feedback from facilitators. Furthermore, the group nature of the practice and evaluated sessions allowed for students to learn from their own performance and that of all other students in the group. Students also strengthened their knowledge and skills when they offered feedback to one another. Fifth, the design of the sessions to limit patient interactions to 10-15 minutes can demonstrate to students the great impact that MI can have in a short period of time, and can increase their confidence that MI can be successfully employed in health care settings in order to improve patient outcomes. Finally, the activity was used to both teach and evaluate student outcomes, and included assessments of both knowledge and skills.

As we implemented this activity, we faced several challenges and learned many lessons to address these issues. The first challenge involved getting student buy-in such that they would enthusiastically engage in the activities. Some medical students felt that it is a doctor's job to simply diagnose and come up with a treatment plan, and they have less value for the role of doctors in motivating patient change. To address this challenge, we implemented a transparent outline (Appendix D) to connect the activity with student's future practice as a physician and to their medical education program objectives. We also included a testimonial from a previous student on how important they found MI to be in their preceptorships and clerkships. We also tried to create a low pressure (students were able to bring in the MI summary sheet to the evaluated session), low stakes (overall pass/fail grade), and positive learning environment where

Copyright © 2021 Jacobs et al. This is an open-access publication distributed under the terms of the Creative Commons Attribution license.

students were praised for proper use of MI skills and encouraged to continue to practice. A second challenge was getting sufficient faculty skilled enough in MI to serve as small-group facilitators. In order to develop a pool of trained faculty, we offered MI training sessions (including continuing medical education credits) to our clinicians. Further, to ensure that we had enough faculty willing to commit the time, we learned to recruit 3-6 months in advance, before clinical schedules were filled. Finally, we faced some challenges with SPs not giving feedback to students that was rooted in MI principles and not showing fidelity to the case scripts. We addressed these challenges by inviting the SPs to the MI PowerPoint presentation that was given to students (Appendix A) so that they could give feedback that reinforced the spirit, principles, and skills of MI (see training/issues for standardized patients in the facilitator's guide, Appendix N). The authors also met with the SP educator directly after the evaluated session to give specific feedback on how SPs portrayed each case, and she immediately met with the SPs to share this feedback.

When the COVID-19 pandemic hit and our courses were required to be taught online instead of in person, we learned that these modules could easily be delivered in an online format, with very minor modifications. Our school of nursing adapted all materials and delivered all sessions online. They used Zoom or Teams to deliver the content, using breakout sessions for the practice and evaluated sessions. All cases were slightly changed to occur in the context of a telemedicine visit. Evaluation forms were translated into fillable PDF forms or surveys. Moving forward, we would like to collect data on these online administrations to see if results are comparable to the in-person training.

In the future, we plan to offer this training to learners in our school of social work, clinical psychology students, as well as students in our physician assistant studies program. Additionally, this activity could serve as an excellent interprofessional learning activity, where students from different health profession disciplines learn together. We would also like to partner with other institutions to evaluate outcomes elsewhere and to increase generalizability of our outcomes. Since the ultimate goal of MI was to improve patient outcomes, it would be wise to follow our learners into their clinical years to see if their skills are maintained over time and with real patients, and to see if their MI skills have any impact on health outcomes of their patients.

Appendices

A. Presurvey.docx
B. MI Presentation.pptx
C. MI Demonstration Script.docx
D. Transparent Outline for MI Activity.docx
E. MICA Evaluation Tool.doc
F. Practice Cases.docx
G. MI Summary Sheet.docx
H. Evaluated Cases.docx
I. OARS Tracking Sheet.doc
J. Change Talk Tracking Sheet.doc
K. MI Evaluated Session Sample Schedule.xlsx
L. Acting Patient Experience Scale.docx
M. Postsurvey.docx
N. Facilitator Guide.docx

All appendices are peer reviewed as integral parts of the Original Publication.

N. Nicole Jacobs, PhD: Associate Professor, Department of Psychiatry and Behavioral Sciences, and Associate Dean of Diversity and Inclusion, University of Nevada, Reno School of Medicine; ORCID: https://orcid.org/0000-0003-2697-3911

Lisa Calvo, MD: Associate Professor, Department of Internal Medicine, and Associate Dean of Medical Education, University of Nevada, Reno School of Medicine.

Aaron Dieringer, MD, MPH: Assistant Professor, Department of Family and Community Medicine, University of Nevada, Reno School of Medicine.

Ali Hall, JD: Trainer and Consultant, Motivational Interviewing Network of Trainers (MINT).

Reka Danko, MD: Clinical Assistant Professor, Department of Internal Medicine, University of Nevada, Reno School of Medicine.

Disclosures
None to report.

Funding/Support
None to report.

Ethical Approval
The University of Nevada, Reno Institutional Review Board approved this study.

References

1. Mokdad AH, Marks JS, Stroup DF, Gerberding JL. Actual causes of death in the United States, 2000. *JAMA*.

Copyright © 2021 Jacobs et al. This is an open-access publication distributed under the terms of the Creative Commons Attribution license.

2004;291(10):1238-1245. https://doi.org/10.1001/jama.291.10.1238

2. Haskard Zolnierek KB, DiMatteo MR. Physician communication and patient adherence to treatment: a meta-analysis. *Med Care*. 2009;47(8):826-834. https://doi.org/10.1097/MLR.0b013e31819a5acc
3. Sahler OJZ, Carr JE, Frank JB, Nunes JV. *The Behavioral Sciences and Health Care*. 4th ed. Hogrefe; 2018.
4. Miller WR. Motivational interviewing with problem drinkers. *Behav Cogn Psychother*. 1983;11(2):147-172. https://doi.org/10.1017/S0141347300006583
5. Miller WR, Rollnick S. *Motivational Interviewing: Preparing People to Change Addictive Behaviors*. Guilford; 1991.
6. Rollnick S, Miller WR, Butler C, Aloia MS. Motivational interviewing in health care: helping patients change behavior. 2008;5(3):203. https://doi.org/10.1080/15412550802093108
7. Hettema J, Steele J, Miller WR. Motivational interviewing. *Annu Rev Clin Psychol*. 2005;1:91-111. https://doi.org/10.1146/annurev.clinpsy.1.102803.143833
8. Lundahl B, Moleni T, Burke BL, et al. Motivational interviewing in medical care settings: a systematic review and meta-analysis of randomized controlled trials. *Patient Educ Couns*. 2013;93(2):157-168. https://doi.org/10.1016/j.pec.2013.07.012
9. Lundahl B, Burke BL. The effectiveness and applicability of motivational interviewing: a practice-friendly review of four meta-analyses. *J Clin Psychol*. 2009;65(11):1232-1245. https://doi.org/10.1002/jclp.20638
10. Rubak S, Sandbæk A, Lauritzen T, Christensen B. Motivational interviewing: a systematic review and meta-analysis. *Br J Gen Pract*. 2005;55(513):305-312.
11. VanBuskirk KA, Wetherell JL. Motivational interviewing with primary care populations: a systematic review and meta-analysis. *J Behav Med*. 2014;37(4):768-780. https://doi.org/10.1007/s10865-013-9527-4
12. Barzilai DA, Goodwin MA, Zyzanski SJ, Stange KC. Does health habit counseling affect patient satisfaction? *Prev Med*. 2001;33(6):595-599. https://doi.org/10.1006/pmed.2001.0931
13. Committee on Health Care for Underserved Women. Motivational interviewing: a tool for behavioral change. *Obstet Gynegol*. 2009;113:243-246.
14. Azari S, Ratanawongsa N, Hettema J, et al. A skills-based curriculum for teaching motivational interviewing-enhanced screening, brief intervention, and referral to treatment (SBIRT) to medical residents. *MedEdPORTAL*. 2015;11:10080. https://doi.org/10.15766/mep_2374-8265.10080
15. Billich W, Bussel I, Felgner J, Berger D, Narasimhan S, Lazarus C. Small changes counseling. *MedEdPORTAL*. 2014;10:9702. https://doi.org/10.15766/mep_2374-8265.9702
16. Boykan R, Blair R, Baldelli P, Owens S. Using motivational interviewing to address tobacco cessation: two standardized patient cases for pediatric residents. *MedEdPORTAL*. 2019; 15:10807. https://doi.org/10.15766/mep_2374-8265.10807
17. Boykan R, Gorzkowski J, Marbin J, Winickoff J. Motivational interviewing: a high-yield interactive session for medical trainees and professionals to help tobacco users quit. *MedEdPORTAL*. 2019;15:10831. https://doi.org/10.15766/mep_2374-8265.10831
18. Day H, Scott R, Fulmer V, et al. Women's health issues in military veterans: standardized patient cases in motivational interviewing, a case materials guide. *MedEdPORTAL*. 2013;9:9539. https://doi.org/10.15766/mep_2374-8265.9539
19. Hartlieb KB, Engle B, Obeso V, Pedoussaut MA, Merlo LJ, Brown DR. Advanced patient-centered communication for health behavior change: motivational interviewing workshops for medical learners. *MedEdPORTAL*. 2016;12:10455. https://doi.org/10.15766/mep_2374-8265.10455
20. Kolasa K, Craven K, Byrd J, Merricks P. Helping your patient change: a patient-centered behavioral counseling presentation for second-year medical students. *MedEdPORTAL*. 2015;11: 10166. https://doi.org/10.15766/mep_2374-8265.10166
21. Parekh K, Benningfield M, Burrows H, et al. Motivational advising workshop: utilizing motivational interviewing theory to facilitate and engage intrinsic motivation to change learners' behavior. *MedEdPORTAL*. 2018;14:10751. https://doi.org/10.15766/mep_2374-8265.10751
22. Stoffers PJ, Hatler C. Increasing nurse confidence in patient teaching using motivational interviewing. *J Nurses Prof Dev*. 2017;33(4):189-195. https://doi.org/10.1097/NND.0000000000000370
23. Tobey M, Marcovitz D, Aragam G. A 1-hour session to refresh motivational interviewing skills for internal medicine residents using peer interviews. *MedEdPORTAL*. 2018;14:10679. https://doi.org/10.15766/mep_2374-8265.10679
24. Windt J, Windt A, Davis J, Petrella R, Khan K. Can a 3-hour educational workshop and the provision of practical tools encourage family physicians to prescribe physical activity as medicine? A pre-post study. *BMJ Open*. 2015;5(7):e007920. https://doi.org/10.1136/bmjopen-2015-007920
25. Söderlund LL, Madson MB, Rubak S, Nilsen P. A systematic review of motivational interviewing training for general health care practitioners. *Patient Educ Couns*. 2011;84(1):16-26. https://doi.org/10.1016/j.pec.2010.06.025
26. Miller WR, Moyers TB. Eight stages in learning motivational interviewing. *J Teach Addict*. 2006;5(1):3-17. https://doi.org/10.1300/J188v05n01_02
27. Boykan R, Messina CR, Blair R. Motivational interviewing to improve Quitline referrals—a resident education initiative. *Am Acad Pediatr*. 2018;142:463. https://doi.org/10.1542/peds.142.1_MeetingAbstract.463
28. Nutley SM, Walter I, Davies HTO. *Using Evidence: How Research can Inform Public Services*. Bristol University Press; 2007.

Copyright © 2021 Jacobs et al. This is an open-access publication distributed under the terms of the Creative Commons Attribution license.

29. *Motivational Interviewing Competency Assessment*. Accessed June 1, 2020. http://micacoding.com

30. *Motivational Interviewing Training New Trainers Manual*. *Motivational Interviewing Network of Trainers*. Updated September 2014. Accessed June 1, 2020. https://motivationalinterviewing.org/sites/default/files/tnt_manual_2014_d10_20150205.pdf

Received: June 7, 2020
Accepted: November 21, 2020
Published: February 12, 2021

Copyright © 2021 Jacobs et al. This is an open-access publication distributed under the terms of the Creative Commons Attribution license.
