# Supplementary material for: Motivational Interviewing Training: A Case-Based Curriculum for Preclinical Medical Students
Source: MedEdPORTAL. 2021 Feb 12;17:11104. doi: 10.15766/mep_2374-8265.11104 (PMC7880250; doi:10.15766/mep_2374-8265.11104)
Supplement: Supplementary file 1 — Presurvey.docxMI Presentation.pptxMI Demonstration Script.docxTransparent Outline for MI Activity.docxMICA Evaluation Tool.docPractice Cases.docxMI Summary Sheet.docxEvaluated Cases.docxOARS Tracking Sheet.docChange Talk Tracking Sheet.docMI Evaluated Session Sample Schedule.xlsxActing Patient Experience Scale.docxPostsurvey.docxFacilitator Guide.docx [file mep_2374-8265.11104-s001.zip › E. MICA Evaluation Tool.doc]

**Motivational Interviewing Competency Assessment**

**Scoring Indicators**

**1**	Fundamentally inconsistent with Motivational Interviewing. Absence of MI Intentions and skills. Missing most elements of MI, and the conversation being coded has no Indicators representing a patient-centered approach.

**2**	Generally inconsistent with Motivational Interviewing. Attempts toward MI are missing the underlying Intentions and skills. May naturally, intentionally, or unintentionally hit elements of MI, yet the conversation coded does not represent a patient-centered approach.

**3**	Consistencies and inconsistencies with notable attempts to align with MI Intentions and skills at a ‘do no harm’ level. Naturally, intentionally, or unintentionally hits elements of MI, yet the conversation being coded represents a patient-centered approach.

**4**	Competent Motivational Interviewing. Primarily consistent with MI Intentions and skills. Intentionally and purposefully focuses on elements of MI, and the conversation coded represents a patient-centered approach.

**5**	Proficient Motivational Interviewing. Adept and consistent with MI Intentions and skills. Deftly orchestrates elements of MI, and the conversation coded embodies a patient-centered approach.

**Scale Definitions**

**Supporting Autonomy & Activation**

This scale is intended to measure the extent to which the MI practitioner encourages and supports the patient’s autonomy and freedom to choose, as well as empowering, addressing, and affirming the patient’s self-efficacy and personal agency. The practitioner works from an assumption that individuals have an innate desire and capacity for evolution and growth. The practitioner operates from a strength-based approach that elucidates and supports the actuation of the patient’s goals, values, and choices.

**Guiding**

This measures the practitioner Intention to navigate the conversation towards the goal of the referral, presenting problem, target behavior, or topic of concern. The patient is the captain whose ship may be stuck, off course, struggling to maintain course, or have no specified course. The patient provides information, feedback and insights while the practitioner helps determine, chart, correct, and maintain a clear course by skillfully navigating the conversation towards a path of insight/solution/resolution.

**Expressing Empathy**

This measures the practitioner’s Intention to actively listen without judgment; grasp the patient’s thoughts, feelings, experiences, and perspective; and, to convey that understanding to the patient. This includes reflective listening, validating the patient’s reality, and all of the efforts the practitioner makes to understand the patient’s inner experience and effectively communicate that to the patient.

**Partnering**

This scale measures the extent to which the practitioner fosters a collaborative process with the patient as two equal partners who are working towards the patient’s goals. There is a shared balance of power, wherein the patient is the acknowledged expert regarding his life. The MI practitioner provides relevant and appropriately timed observations, knowledge, insights, and expertise that supports and advances patient outcomes.

**Evoking**

This measures the extent to which the practitioner elicits the patient’s perspective on his own thoughts, barriers, knowledge, feelings, ideas, motivators, goals, values, and solutions regarding the target behavior and change. The practitioner operates both from a place of genuine curiosity and from a belief that the motivation for change and the ability to change exists within the patient and focuses efforts to skillfully elicit, explore, and expand those patient perspectives.

**Motivational Interviewing Competency Assessment**

| **SUPPORTING AUTONOMY & ACTIVATION** | ○ **1** | ○ **2** | ○ **3** | ○ **4** | ○ **5** |
| --- | --- | --- | --- | --- | --- |
|  | Primary focus is on the practitioner or agency goals/values for the interaction with no interest in perception of patient choice/control. | Cursory or paternalistic focus on patient goals/values and superficial attention to patient choice/control. | Appropriate efforts to focus on patient goals/values with attempts to acknowledge patient choice/control. | Successfully focuses on patient goals/values and self-efficacy while fostering informed choice/control. | Skillfully empowers patient through focus on values/goals and markedly enhances patient sense of choice/control. |
|  | **Examples/Rationale for Rating:** | | | | |

| **GUIDING** | ○ **1** | ○ **2** | ○ **3** | ○ **4** | ○ **5** |
| --- | --- | --- | --- | --- | --- |
|  | Primarily directing, controlling, or mandating the patient goals. | Primarily wandering around, following, or subtly directing patient goals. | Attempts a shared approach to the patient’s goals with instances of wandering, following or directing. | Successfully identifies a shared approach to the patient’s goals. | Skillfully delineates a shared approach to patient’s goals. |
|  | **Examples/Rationale for Rating:** | | | | |

| **EXPRESSING EMPATHY** | ○ **1** | ○ **2** | ○ **3** | ○ **4** | ○ **5** |
| --- | --- | --- | --- | --- | --- |
|  | Dismisses, ignores, or has little interest in patient perspective, feelings or experiences. | Random, sporadic, or tepid efforts to understand patient perspective. | Appropriate efforts to understand patient perspective with moderate success. | Successfully communicates an understanding of patient perspective. | Skillfully conveys a multifaceted understanding of patient’s perspective. |
|  | **Examples/Rationale for Rating:** | | | | |

| **PARTNERING** | ○ **1** | ○ **2** | ○ **3** | ○ **4** | ○ **5** |
| --- | --- | --- | --- | --- | --- |
|  | Exerts the expert role by defining the patient problem and prescribing/developing the goals and/or plan. | Leans toward expert role and consistently misses or unaware of opportunities to collaborate. | Appropriate efforts to collaborate. | Successfully fosters collaboration and power sharing. | Skillfully collaborates and empowers patient as the lead. |
|  | **Examples/Rationale for Rating:** | | | | |

| **EVOKING** | ○ **1** | ○ **2** | ○ **3** | ○ **4** | ○ **5** |
| --- | --- | --- | --- | --- | --- |
|  | Absence of curiosity and exploration of patient perspective. | Minimal curiosity and incomplete efforts to elicit patient perspective. | Appropriate efforts to elicit and explore patient perspective. | Successfully elicits and explores patient perspective. | Skillfully elicits, explores, and expands patient perspective. |
|  | **Examples/Rationale for Rating:** | | | | |

**OVERALL STRENGTHS**

**AREAS FOR IMPROVEMENT**

Overall Grade: _____Fail	_____ Pass
