# Supplementary material for: Motivational Interviewing Training: A Case-Based Curriculum for Preclinical Medical Students
Source: MedEdPORTAL. 2021 Feb 12;17:11104. doi: 10.15766/mep_2374-8265.11104 (PMC7880250; doi:10.15766/mep_2374-8265.11104)
Supplement: Supplementary file 1 — Presurvey.docxMI Presentation.pptxMI Demonstration Script.docxTransparent Outline for MI Activity.docxMICA Evaluation Tool.docPractice Cases.docxMI Summary Sheet.docxEvaluated Cases.docxOARS Tracking Sheet.docChange Talk Tracking Sheet.docMI Evaluated Session Sample Schedule.xlsxActing Patient Experience Scale.docxPostsurvey.docxFacilitator Guide.docx [file mep_2374-8265.11104-s001.zip › I. OARS Tracking Sheet.doc]

**Observer Tracking Worksheet: OARS**

| **Strategy** | **Count (Tally)** | **Comments (e.g. elicited change talk, enhanced confidence/readiness, created resistance etc.)** |
| --- | --- | --- |
| Open Questions |  |  |
| Closed Questions |  |  |
| Affirm |  |  |
| Reflection |  |  |
| Summary |  |  |
| Additional comments regarding overall use of OARS: | | |
