# Supplementary material for: Motivational Interviewing Training: A Case-Based Curriculum for Preclinical Medical Students
Source: MedEdPORTAL. 2021 Feb 12;17:11104. doi: 10.15766/mep_2374-8265.11104 (PMC7880250; doi:10.15766/mep_2374-8265.11104)
Supplement: Supplementary file 1 — Presurvey.docxMI Presentation.pptxMI Demonstration Script.docxTransparent Outline for MI Activity.docxMICA Evaluation Tool.docPractice Cases.docxMI Summary Sheet.docxEvaluated Cases.docxOARS Tracking Sheet.docChange Talk Tracking Sheet.docMI Evaluated Session Sample Schedule.xlsxActing Patient Experience Scale.docxPostsurvey.docxFacilitator Guide.docx [file mep_2374-8265.11104-s001.zip › J. Change Talk Tracking Sheet.doc]

Observer Tracking Worksheet: Change Talk

| **Change Talk** | **Tally** | Comment (how did the therapist elicit the change talk?) |
| --- | --- | --- |
| **DESIRE**  Intention to change  I want to/should |  |  |
| **ABILITY**  Optimism for change  I can |  |  |
| **REASON**  problem recognition/ awareness of advantages of change  I know I have problem…. |  |  |
| **NEED**  Concern about problem  If I don’t…… |  |  |
| **COMMITMENT**  I will/am going to |  |  |
